# 1-Titanacyclobuta-2,3-diene – an elusive four-membered cyclic allene†
†Electronic supplementary information (ESI) available: Experimental, spectroscopic, crystallographic and computational details (PDF); *xyz* coordinates. CCDC 1897219 and 1897220. For ESI and crystallographic data in CIF or other electronic format see DOI: 10.1039/c9sc01002e


**DOI:** 10.1039/c9sc01002e

**Published:** 2019-04-23

**Authors:** Fabian Reiß, Melanie Reiß, Jonas Bresien, Anke Spannenberg, Haijun Jiao, Wolfgang Baumann, Perdita Arndt, Torsten Beweries

**Affiliations:** a Leibniz-Institut für Katalyse e.V. an der Universität Rostock , Albert-Einstein-Str. 29a , 18059 Rostock , Germany . Email: fabian.reiss@catalysis.de ; Email: torsten.beweries@catalysis.de; b Abteilung für Anorganische Chemie , Institut für Chemie , Universität Rostock , Albert-Einstein-Straße 3a , D-18059 Rostock , Germany

## Abstract

The synthesis of an unusual 1-metalla-2,3-cyclobutadiene complex [*rac*-(ebthi)Ti(Me_3_SiC_3_SiMe_3_)] (*rac*-ebthi = *rac*-1,2-ethylene-1,1′-bis(η^5^-tetrahydroindenyl)), a formal metallacyclic analogue of a non-existent four-membered 1,2-cyclobutadiene, is described. By variation of the cyclopentadienyl ligand of the titanocene precursor it was possible to stabilise this highly exotic compound which selectively reacts with ketones and aldehydes to yield enynes by oxygen transfer to titanium. Analysis of the bonding and electronic structure of the metallacycle shows that the complex is best described as an unusual antiferromagnetically coupled biradicaloid system, possessing a formal Ti(iii) centre coordinated with a monoanionic radical ligand.

## Abstract 2

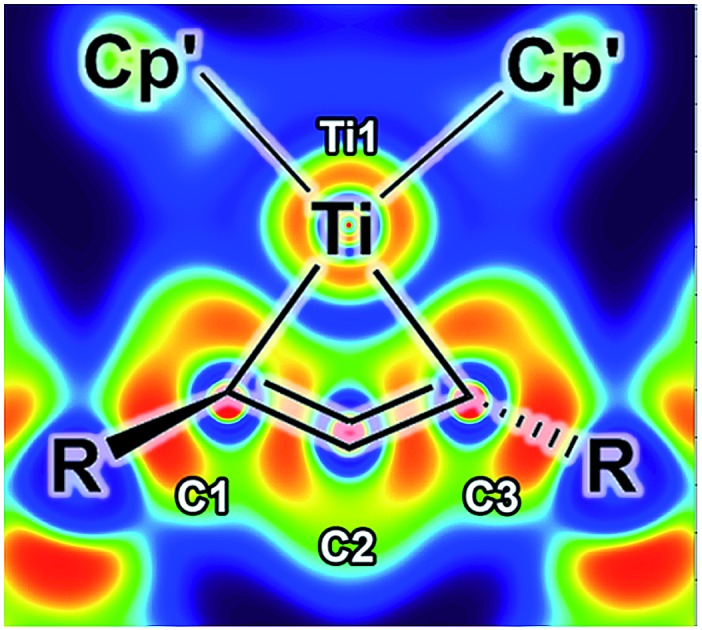
The synthesis and characterisation of a 1-titanacyclobuta-2,3-diene complex, an organometallic analog of elusive 1,2-cyclobutadiene, is presented.

## Introduction

**Fig. 1:**
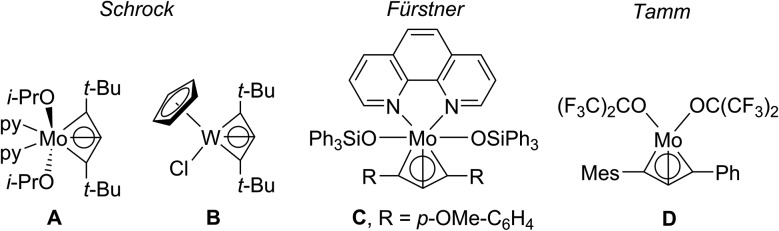
Structurally characterised 1-metallacyclobuta-2,3-diene complexes. Bonding situations in the metallacycle are depicted as shown in the literature.

Driven by the “desire to make the molecule that violates the norm”^1^ chemists have always looked into possibilities to stabilise exotic molecules and explore the reasons for their existence and reactivities. In this respect, a commonly used approach is the incorporation of unsaturation into cyclic structures that increases the ring strain and therefore in principle decreases the likeliness of its existence.^2^ For a long time, organometallic chemists have investigated metallacycles, both, with respect to their unusual coordination environments as well as potential applications in stoichiometric^3^,^4^ and catalytic transformations.^4^,^5^ In the past, a number of five-membered metallacycles based on early and late transition metals have been reported that possess rather unusual structural motifs, including Rosenthal's 1-metallacyclopenta-2,3,4-trienes,^6^ Suzuki's 1-metallacyclopent-3-ynes,^7^ Erker's 1-metallacyclopenta-3,4-dienes^8^ (all based on group 4 metals) or Xi's metalloles (Rh, Pd, Pt).^9^ Intuitively, the synthesis of four-membered and highly unsaturated metallacycles should be even more challenging and to date a common approach to stabilise such structures is the incorporation of heteroatoms into the ring system.^10^ For example, the parent 1,2-cyclobutadiene has a calculated strain energy of 74.9 kcal mol^–1^,^11^ thus reflecting the instability and the difficulty in isolating a molecule of this type. The chemistry of 1-metallacyclobuta-2,3-dienes has been investigated in the past as such structures play a role for catalyst deactivation in alkyne metathesis. Schrock reported on two structurally characterised examples of “all-C-deprotiometallacyclobutadienes” [Mo{C_3_-(*t*Bu)_2_}{OCH(CF_3_)_2_}_2_(py)_2_] (**A**)^12^ and [CpW{C_3_(*t*Bu)_2_}-Cl] (**B**)^13^ (Cp = η^5^-cyclopentadienyl) that were formed during metathesis of terminal alkynes (Fig. 1). Later, Fürstner (**C**) and – very recently Tamm (**D**) – observed the formation of 1-molybdacyclobuta-2,3-dienes as a product of reaction of an alkylidene catalyst with a terminal alkyne.^14^

To further explore the frontiers of the chemistry of group 4 metallacycles Jemmis, Schulz and Rosenthal have computationally investigated the possibilities to stabilise planar 1-metalla-2,3-cyclobutadienes and found that the incorporation of electron-donating substituents at α-carbon atoms could be beneficial.^11^ Later, our group has attempted to synthesise such structures by coupling of alkynyl and isonitrile ligands at titanocene, unfortunately this approach only resulted in redox-disproportionation of the Ti centre.^15^ Also, deprotonation of α-CH_2_SiMe_3_ and α-CH_2_N(SiMe_3_)_2_ substituted titanocene alkyne complexes was found to be unsuccessful.^16^ A more promising approach appeared to be the initial construction of a C_3_ framework prior to coordination to the metal. We have thus revisited and optimised the synthesis of a previously reported 1,3-dilithioallene precursor [Li_2_(Me_3_SiC_3_SiMe_3_)]^17^ and reacted this with [Cp_2_ZrCl_2_]. To our surprise, we obtained two unusual allenediide bridged dizirconocene complexes [Cp_2_Zr(RC_3_R)_2_ZrCp_2_] and [Cp_2_Zr(Cl)RC_3_R(Cl)ZrCp_2_] (R = SiMe_3_),^18^ the latter being a promising precatalyst for amine borane dehydropolymerisation.^19^ Interestingly, we were able to show that the dizirconacyclooctatetraene [Cp_2_Zr(RC_3_R)_2_ZrCp_2_] degrades under mass spectrometry conditions into the desired mononuclear 1-zirconacyclobuta-2,3-diene compound. In this contribution, we report on the synthesis and characterisation of the first 1-metalla-2,3-cyclobutadiene complex of a group 4 metal as well as the reactivity of this unusual complex.

## Results and discussion

### Reaction of [Cp_2_TiCl_2_] and 
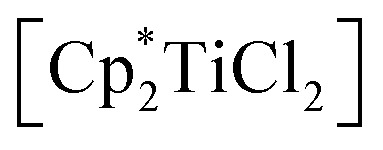
 with the 1,3-dilithioallene precursor

**Scheme 1:**
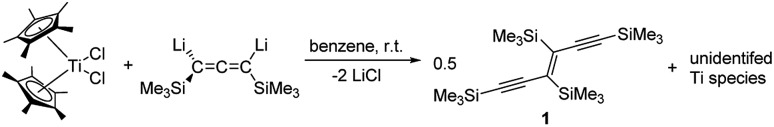
Reaction of 
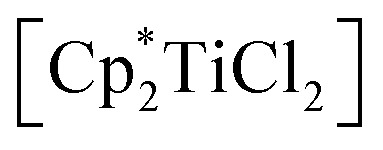
 with [Li_2_(Me_3_SiC_3_SiMe_3_)].

To evaluate the influence of the metal centre we first adapted our previously reported procedure^18^ and reacted [Cp_2_TiCl_2_] with [Li_2_(Me_3_SiC_3_SiMe_3_)]. ^1^H NMR analysis of an aliquot taken after a reaction time of 16 hours at room temperature shows the formation of several Cp containing products along with a large number of resonances in the SiMe_3_ region of the spectrum, out of which we identified the coupling product **1** (*cf.* p. S4, Fig. S1†). Even at low temperatures (–40 °C), only similar product mixtures could be isolated. We next employed more electron-donating, sterically more demanding 
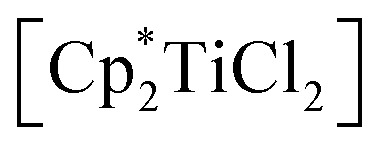
 (Cp* = η^5^-pentamethylcyclopentadienyl) and observed a slow colour change from red to brown. NMR analysis of the reaction mixture shows the formation of unidentified titanocene species along with the coupling product **1** as the main species, which can be isolated after column chromatographic workup in 90% yield (Scheme 1). The formation of **1** can be formally seen as the dimerisation of two carbenes in the coordination sphere of Ti. A similar type of carbene coupling to form alkynyl functionalised symmetrical olefins was reported before by Casey and co-workers for a Re system.^20^ Also, the formation of Ph_4_C_6_ by thermal/radical degradation of 1,3-bis(alkylseleno)allenes supports this proposal.^21^

In a previous study we had computed the isodesmic exchange reaction of 
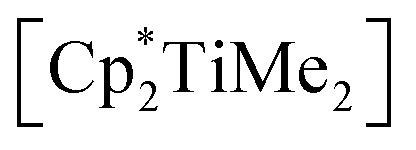
 and the allene precursor Me(Me_3_Si)C_3_(SiMe_3_)Me to form the desired four-membered metallacycle and ethane. This reaction is slightly exergonic and thus in principle feasible (Δ*G* = –1.75 kcal mol^–1^,^22^). This approach was chosen as a theoretical model for salt metathesis using a 1,3-dilithioallene to avoid solubility effects and cluster formation that would complicate computational analysis. For the reaction of [Cp_2_TiCl_2_] with [Li_2_(Me_3_SiC_3_SiMe_3_)] at low temperature we proposed formation of a 1-titana-2,3-cyclobutadiene (*vide supra*), which is in accordance with the computed exergonic reaction of Cp_2_TiMe_2_ and Me(Me_3_Si)C_3_(SiMe_3_)Me (Δ*G* = –11.77 kcal mol^–1^). The fact that a coupling reaction leading to **1** was observed in reactions with Cp and Cp* complexes indicates that at some stage of the reaction the interaction of an intermediately formed C_3_ framework with the Ti centre occurred. *ansa*-Cp complexes in many cases show reactivities that are comparable to Cp analogues and furthermore exhibit additional stabilisation of the metallocene through the bridging unit. Subtle differences in reactivity (*e.g.* formation of different products or faster conversion) are often referred to as the *ansa* effect.^23^ In line with this, the reaction of [*rac*-(ebthi)TiMe_2_] and Me(Me_3_Si)C_3_(SiMe_3_)Me is even more thermodynamically favourable (Δ*G* = –16.60 kcal mol^–1^).

### Synthesis and characterisation of 1-titanacyclobuta-2,3-diene (**2**)

**Scheme 2:**
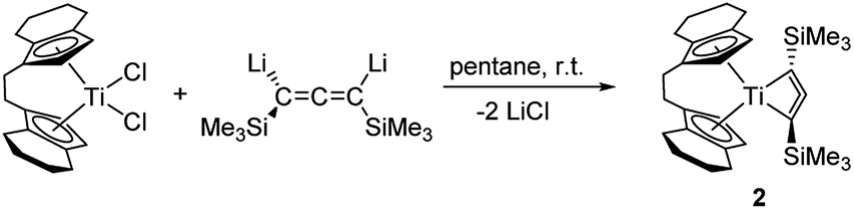
Reaction of [*rac*-(ebthi)TiCl_2_] with [Li_2_(Me_3_SiC_3_SiMe_3_)] and formation of **2**.

We have thus next reacted [*rac*-(ebthi)TiCl_2_] and [Li_2_(Me_3_SiC_3_SiMe_3_)] at room temperature in pentane (Scheme 2) and observed formation of a deep red solution, from which compound **2** could be isolated as red crystals after removal of LiCl. Moderate yields of **2** (58%) can be explained by the formation of **1** as a byproduct which was identified by NMR spectroscopy and this nicely demonstrates the subtle differences in reactivity along the series Cp–*rac*-(ebthi)–Cp*. Single crystals suitable for X-ray analysis were obtained from a saturated pentane solution at room temperature.

Compound **2** shows the expected characteristic ^1^H NMR signals at 0.35, 5.27, and 7.22 ppm, corresponding to the SiMe_3_ groups and the Cp protons of the *rac*-(ebthi) ligand. The ^13^C NMR spectra show two signals at low field that we assign to the internal (*δ* 134.2 ppm) and metal-bound carbon atoms (*δ* 213.8 ppm) of the metallacycle. Notably, these values strongly differ from those found for the previously reported Mo and W complexes, pointing towards structural differences such as the absence of a M–C2 interaction (Δ*δ* > 56 ppm, Table 1).

**Table 1:** Comparison of ^13^C NMR values for metallacyclic units in complexes **2**, **A**, **B**, and **C**

	**A** 12	**B** 13	**C** 14	**2**	[Li_2_(Me_3_SiC_3_SiMe_3_)]^18^
M–*C*	220.4	257	205.3	213.8	37.8, 44.6^*a*^
C–*C*–C	197.1	196	190.6	134.2	174.1

^*a*^Tetrameric compound in the solid state, dynamic behaviour in solution leads to two inequivalent ^13^C shifts.

**Fig. 2:**
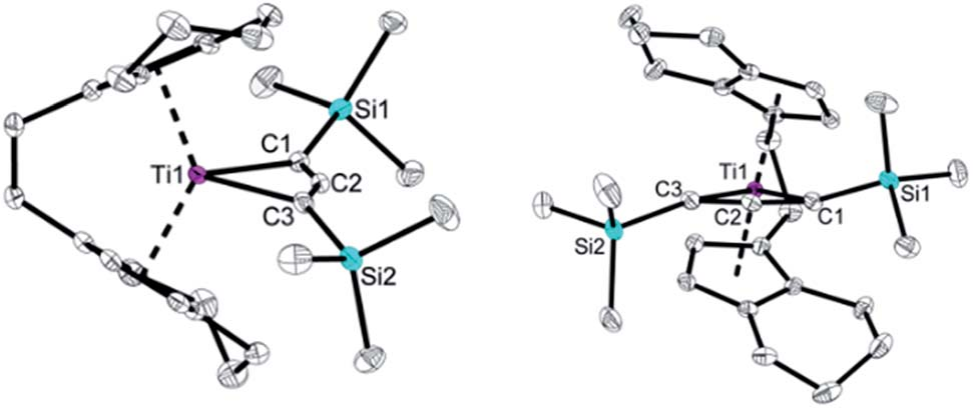
Two views of the molecular structure of complex **2**. Thermal ellipsoids correspond to 30% probability. Hydrogen atoms are omitted for clarity.

The molecular structure of compound **2** (Fig. 2) shows the bent metallocene fragment as part of the four-membered ring system. Ti–C bond lengths are significantly longer than typical single bonds (Ti1–C1 2.229(1), Ti1–C3 2.235(2); Σr_cov_ = 2.11 Å 24). That the Ti1–C2 distance (2.178(1) Å) is shorter than Ti1–C1/C3 indicates that the C_3_ unit is only slightly bent, which is corroborated by the angle C1–C2–C3 of 150.1(2)°. Compared to the Mo and W complexes this C_3_ unit in **2** is less bent (*cf.* 130°–135°). The C–C bond distances are in the range of double bonds (C1–C2 1.303(2), C2–C3 1.308(2) Å; Σr_cov_ = 1.34 Å 24) and shorter compared to values found for Mo and W complexes, most likely due to less electron donation to the metal centre. While the Ti1–C1–C2–C3 unit is planar (1.1(3)°) the SiMe_3_ groups are located above and below this plane, which is in contrast to the structural features reported for group 6 metallacyclobutadienes (*cf.* p. S15 Table S4,†). This description of a C=C=C unit in **2** is supported by the observed in-phase (1344 cm^–1^) and out-of-phase (1729 cm^–1^) vibrations which are in good agreement with calculated values (1343 cm^–1^/1787 cm^–1^).^22^

### Analysis of the structure and bonding of **2**

**Fig. 3:**
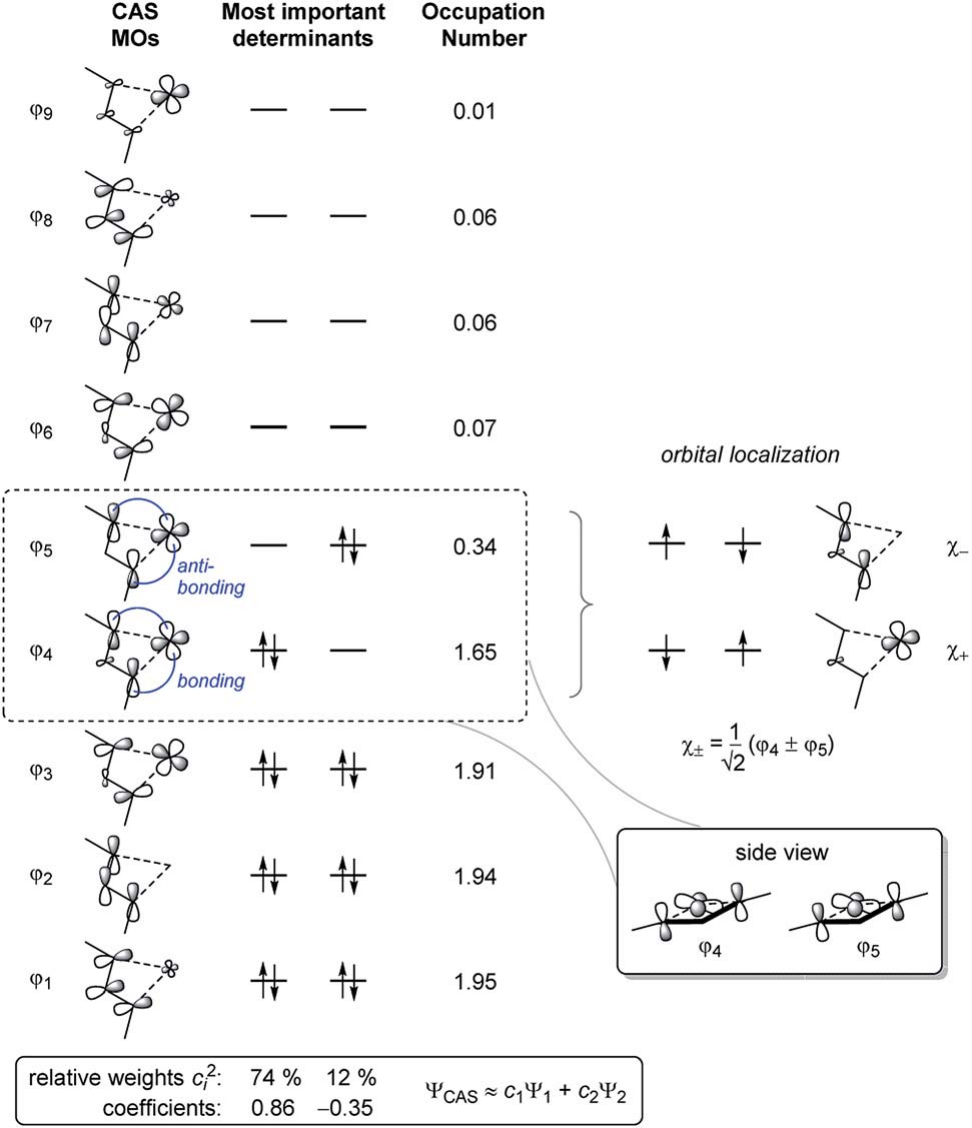
Schematic depiction of the active orbitals of a CAS(8,9) calculation. Only contributions to the wave function with relative weights > 1% are shown. The orbital localisation scheme indicates that one of the radical centres is localised at Ti, while the other is delocalised across the C_3_ backbone.

To obtain a better understanding of the bonding situation in 1-titanacyclobuta-2,3-diene **2**, a series of density functional theory (DFT) and wave function theory (WFT) calculations were performed. Firstly, the structure of **2** was optimised at the BP86 25/LANL2DZ^26^/TZVP^27^ level of theory, showing good agreement with structural data from single-crystal X-ray diffraction (*cf.* p. S48, Table S12†). At this point we noticed a small energy gap between the HOMO and LUMO (1.0 eV), which can be indicative of substantial biradical character. To further evaluate this, the optimised structure was used for several single point calculations employing the pure and hybrid density functionals (DF) BP86 and B3LYP^24^,^28^ to calculate the Kohn–Sham (KS) orbitals, as well as HF to compute the canonical MOs. All (KS) wave functions were tested with respect to RHF/UHF or RKS/UKS instabilities, in order to analyse the biradical character of Ti complex **2**. While the KS wave function based on the pure DF (BP86) showed no instabilities, the hybrid DF (B3LYP) and HF solution exhibited a low-lying, “broken-symmetry” open-shell singlet state. This kind of behaviour is often observed if the biradical character is not too large,^29^ since part of the non-dynamic correlation is treated by the exchange–correlation functional of the (pure) density functional. Mixing in exact exchange reduces the amount of correlation treated by the DF and thus the “broken-symmetry” solution becomes more stable. While the structures that were optimised using the BP86 functional show good agreement with experimental structures (Table S12†), the electronic energy should only be considered as a rough approximation due to incorrect treatment of the non-dynamic correlation. Therefore we employed the Complete Active Space (CAS(8,9)) SCF method^30^ to obtain a multi-determinant open-shell singlet wave function and describe the bonding situation in **2** appropriately. This calculation determined the biradical character of **2** with *β* = 28%,^31^,^32^ and identified the largest contributions to the overall wave function as the two determinants placing two electrons either in the formal HOMO (*φ*_4_) or LUMO (*φ*_5_, Fig. 3).

**Scheme 3:**
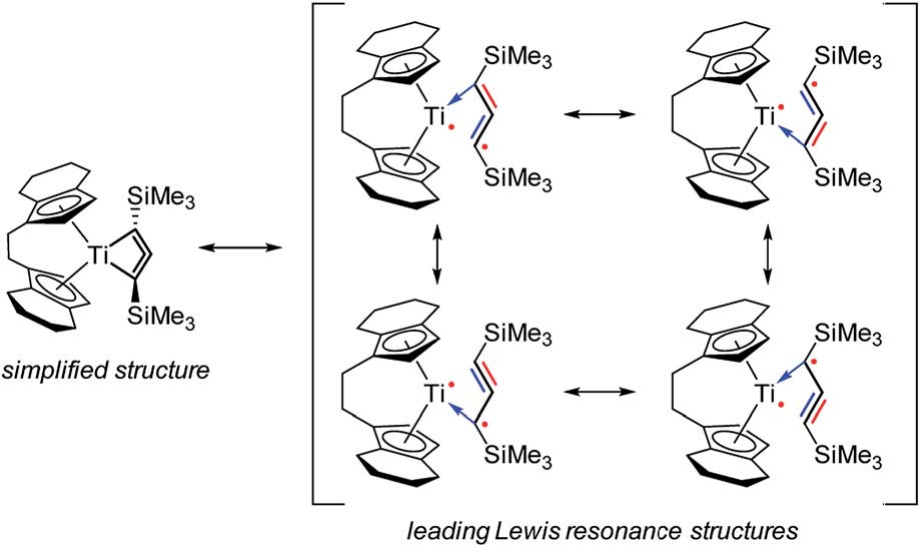
Main resonance structures which best describe the bonding situation in **2** as singlet biradical. The antiferromagnetically coupled electron at the ligand is delocalised within the out-of-plane π_*x*_ bonding system (red), while the in-plane π_*z*_ orbitals (blue) contribute to the C–Ti bonding interactions.

The singlet state is calculated to be the ground state (Δ*E*_S–T_ = –9.3 kcal mol^–1^); *i.e.* the radical centres are antiferromagnetically coupled and the calculated exchange coupling constant^33^ is 2*J* = *E*_S_ – *E*_T_ = –3260 cm^–1^. The radical centres are localised at Ti and on the C_3_ backbone of the ligand (Fig. 3, right). Therefore, the electronic structure can be understood as a complex possessing a formal Ti(iii) fragment and an organic radical, where the “free” electrons are antiferromagnetically coupled. As a consequence, no classical Ti(iii) chemistry would be expected as the complex is a singlet. In conjunction with results from Natural Bond Orbital (NBO) analysis, the electronic situation is best described by the main Lewis-type resonance structures depicted on the right side of Scheme 3. Note that the electrons in both the formal π_*x*_ and π_*z*_ bonding systems of the formal propadienylide ligand are delocalized across the C_3_ unit and that each of these π-bonding systems can be interpreted independently of the other, resulting in a variety of different Lewis resonance structures (see also Fig. S50†). The calculated natural charge of the C_3_(SiMe_3_)_2_ ligand amounts to –0.39*e* (CAS) or –0.64*e* (BP86), which is in the expected range of a formally anionic ligand. For a more detailed discussion of the CAS wave function and bonding situation, please refer to the ESI, pp. S49.†

**Fig. 4:**
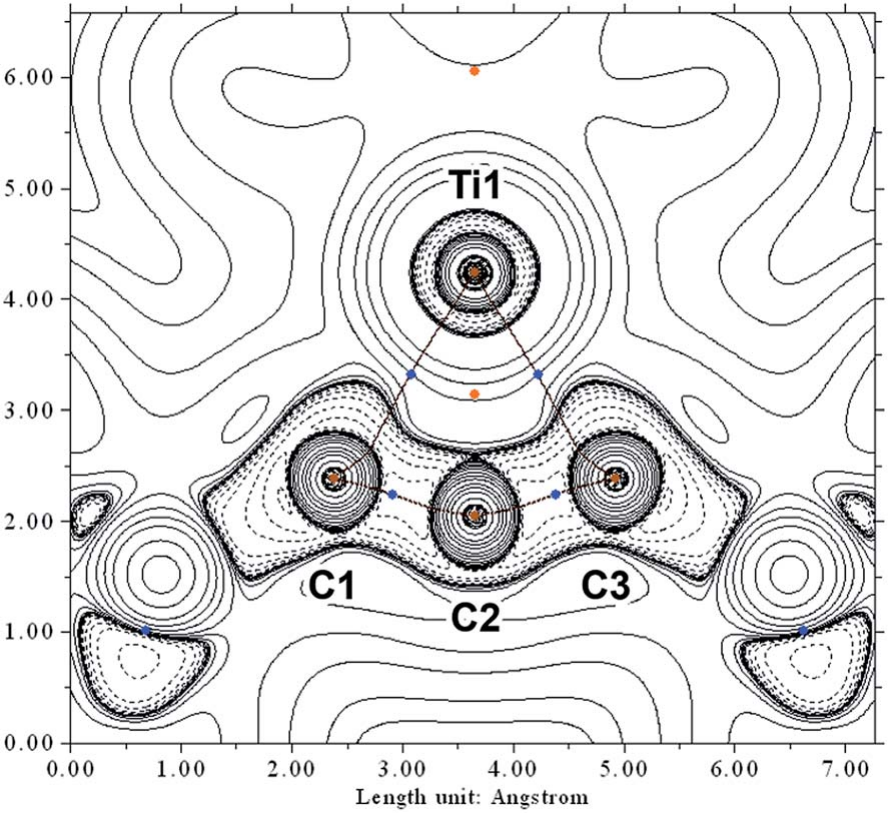
Contour plot of the Laplacian of the electron density ∇^2^*r* of Ti complex **2** in the TiC_3_ ring plane. Dashed lines indicate negative (local charge concentration), solid lines indicate positive values (local charge depletion). The Laplacian plot is overlaid with the molecular graph from QT-AIM analysis. Brown lines indicate bonding paths, blue dots correspond to bond critical points, orange points indicate ring critical points. Density from CAS(8,9)/def2-TZVP calculation.

In order to investigate the topology of the electron density, we performed a QT-AIM analysis.^34^,^35^ This revealed two Ti–C bond paths (Ti1–C1 and Ti1–C3), in agreement with the Lewis resonance scheme (Scheme 3). Despite the short interatomic distance between Ti1 and C2, there is no strong bonding interaction between those atoms; on the contrary, a ring critical point is found near the centre of the TiC_3_ ring system. Moreover, the Laplacian of the electron density ∇^2^*r* indicates that the Ti–C bonds are strongly polarised towards the C atoms (Fig. 4).

The densities obtained from CAS(8,9) and BP86 calculations are similar, indicating that the pure DFT method is suitable to approximately describe the electron density despite its single-determinant character (*cf.* p. S53, Fig. S52†). The results from QT-AIM analysis are corroborated by ELF analysis (*cf.* p. S53, Fig. S53†). There is no localised electron density in the valence region of C2 directed towards Ti1, whereas the bonding electrons between C1/C3 and Ti are localised in the same area as indicated by the Laplacian of the electron density. Notably, there is no localised electron density around C2 pointing away from Ti1 either, *i.e.* there is no lone pair of electrons at the central carbon atom. Consequently, the electronic structure of the C_3_ scaffold is different from that of structurally related bent allenes, such as so-called “carbodicarbenes” (*cf.* p. S54, Fig. S54†).^36^

With these results in mind we calculated the biradical character of the hypothetical analogous Cp (*β* = 30%) and Cp* (*β* = 74%) substituted complexes and found that the singlet–triplet gap and therefore the biradical character greatly depend on the pyramidalisation (hybridisation) of the carbon atoms C1 and C3 of the TiC_3_ ring. Since the coordination environment around C1/C3 is nearly planar in 
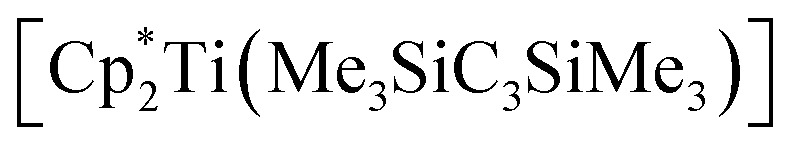
, it shows the highest biradical character. This trend is in agreement with previous computations^11^ and provides a possible explanation for the selective formation of **1** over a highly reactive planar biradical complex.

### Reactions of **2** with carbonyl compounds

To gain first insights into the reactivity of the isolable and unusual biradical [*rac*-(ebthi)Ti(Me_3_SiC_3_SiMe_3_)] complex we have performed stability tests. Therefore, we exposed solutions of **2** to air and moisture and found that, in both cases, slow formation of the propyne Me_3_SiC_2_CH_2_SiMe_3_ takes place (see ESI† for details). To evaluate whether our compound shows radical-type reactivity despite its biradical character, we have added 9,10-dihydroanthracene as a potential radical trap, however, we observed no conversion at room temperature. In reactions with TEMPO ((2,2,6,6-tetramethylpiperidin-1-yl)oxyl) formation of ill-defined reaction mixtures that contain Ti(iii) species takes place.

**Scheme 4:**
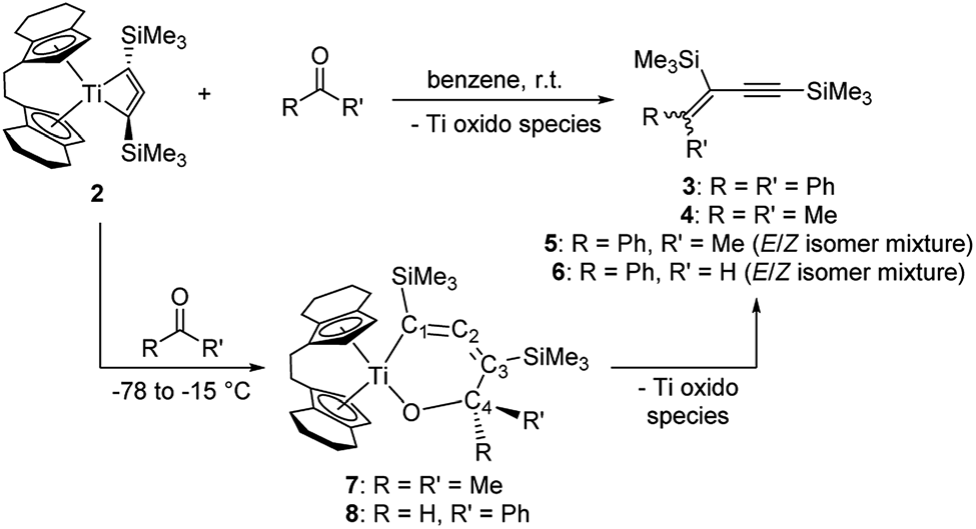
Reaction of **2** with ketones to yield enynes **3**, **4**, **5** and **6**.

We next focused on classical biradical-type reactions that are also known for inorganic cyclic four-membered 1,3-biradical systems.^37^ In the reaction with CO_2_, a colour change from red to teal and finally brownish was observed. The NMR spectra of this sample indicate the formation of three so far unidentified organometallic products and thus suggest a high reactivity towards carbonyl substrates. However, these species could not be separated and characterised yet. Based on this observation we have next evaluated the reactivity of **2** towards simple carbonyl compounds benzophenone, acetone, acetophenone, and benzaldehyde (Scheme 4).

NMR analysis of reaction solutions showed that in all cases formation of enynes occurred by formal oxygen transfer to Ti and coupling of the remaining C_1_ unit with the C_3_ moiety of the metallacyle. The molecular structure of compound **3** (*cf.* p. S14, Fig. S7†) confirms the assignment as an enyne.

The nature of the Ti species formed by oxygen transfer could not be unequivocally clarified; however, formation of a Ti oxido species is likely. It should however be noted that such species require either kinetic stabilisation by bulky Cp ligands,^38^ or additional Lewis bases such as pyridines^39^ to persist as monomeric well-defined complexes. Monitoring of the reaction of **2** and acetone at low temperature, *i.e.* slow warming from –78 °C, showed that the red colour of **2** is retained up to a temperature of –15 °C, at which point the solution turned teal. After workup at low temperature, the dark-teal residue was analysed by low-temperature NMR spectroscopy. ^13^C NMR spectra showed four new resonances due to a new metallacyclic species **7** at 179.7 (C2), 151.9 (C1), 109.4 (C3), and 90.3 ppm (C4), thus suggesting that the well-known insertion of the ketone^40^ is the first step of C–C bond formation that ultimately yields the enyne (Scheme 4). It should however be noted that this reaction of carbonyl compounds with **2** can also be regarded as an addition reaction to a 1,2-biradical with addition of the C=O bond to both radical centres. Looking at the nature of the reaction products and possible intermediates this cannot be distinguished from classical Ti(iv) diyl insertion reactivity.

Remarkably, in a similar low temperature experiment with **2** and benzaldehyde we detected only the intermediate species **8** which is consistent with a structure of the type **7** (^13^C NMR of **8**: 179.5 (C2), 154.2 (C1), 109.1 (C3), 91.8 ppm (C4)). Thermodynamic calculations reveal intermediate **8** as the sterically less stressed isomer which is preferred by –5.2 kcal mol^–1^ (*cf.* p. S42, Fig. S48†).^22^ This [3 + 1] coupling mode is rather unusual for a 1,3-enyne as these compounds are typically prepared by dimerisation of terminal alkynes,^41^ or by Pd catalysed Sonogashira coupling reactions.^42^ Similar carbene transfer reagents include Tebbe's and Petasis' Cp_2_Ti=CH_2_ reagent,^43^ however, in the herein described case transfer of a C_3_ unit is possible. Moreover, the present reaction pattern is reminiscent of earlier work on retro-cycloaddition using group 4 metallocene oxido or imido complexes.^44^ It should however be noted that transformation of the herein described reaction into a catalytic protocol might be hampered by the oxophilicity of the Ti centre.

## Conclusions

For the first time, we have shown that through combination of suitable metal centre, cyclopentadienyl ligand and substituents at the C_3_ unit the synthesis of an elusive 1-metallacyclobuta-2,3-diene based on a group 4 metal is possible. Whereas for Cp and Cp* substituted complexes only formation of an unsaturated six-membered chain product was observed, possibly due to coupling of two C_3_ carbene moieties, in case of *rac*-(ebthi) formation of the desired 1-titanacyclobuta-2,3-diene was possible. This compound represents a formal metallacyclic analogue of non-existent 1,2-cyclobutadiene. Analysis of the structure and bonding reveals a highly unusual interaction of a formal Ti(iii) fragment and an organic monoanionic radical with antiferromagnetic coupling between both radical centres, resulting in a singlet species. Reaction of the metallacycle with carbonyl compounds yields enynes *via* an unusual [3 + 1] coupling with formal insertion of the C=O bond into the M–C bond being the first step of the reaction. Further studies to understand the structural principles as well as further investigations regarding the reactivity of **2** and related complexes will be done in our lab in the future.

## Conflicts of interest

There are no conflicts to declare.

## Supplementary Material

Supplementary information

Crystal structure data

## References

[cit1] Hoffmann R., Hopf H. (2008). Angew. Chem., Int. Ed..

[cit2] Johnson R. P. (1989). Chem. Rev..

[cit3] Rosenthal U., Burlakov V. V., Bach M. A., Beweries T. (2007). Chem. Soc. Rev..

[cit4] Beaumier E. P., Pearce A. J., See X. Y., Tonks I. A. (2019). Nat. Rev. Chem..

[cit5] Herrmann W., Böhm V. P. W., Reisinger C.-P. (1999). J. Organomet. Chem..

[cit6] Rosenthal U., Ohff A., Baumann W., Kempe R., Tillack A., Burlakov V. V. (1994). Angew. Chem., Int. Ed. Engl..

[cit7] Suzuki N., Nishiura M., Wakatsuki Y. (2002). Science.

[cit8] Ugolotti J., Dierker G., Kehr G., Fröhlich R., Grimme S., Erker G. (2008). Angew. Chem., Int. Ed..

[cit9] Wei J., Zhang W.-X., Xi Z. (2018). Chem. Sci..

[cit10] Beweries T., Haehnel M., Rosenthal U. (2013). Catal. Sci. Technol..

[cit11] Roy S., Jemmis E. D., Schulz A., Beweries T., Rosenthal U. (2012). Angew. Chem., Int. Ed..

[cit12] McCullough L. G., Schrock R. R., Dewan J. C., Murdzek J. C. (1985). J. Am. Chem. Soc..

[cit13] McCullough L. G., Listemann M. L., Schrock R. R., Churchill M. R., Ziller J. W. (1983). J. Am. Chem. Soc..

[cit14] Heppekausen J., Stade R., Kondoh A., Seidel G., Goddard R., Fürstner A. (2012). Chem.–Eur. J..

[cit15] Reiß F., Altenburger K., Hollmann D., Spannenberg A., Jiao H., Arndt P., Rosenthal U., Beweries T. (2017). Chem.–Eur. J..

[cit16] Reiß F., Reiß M., Spannenberg A., Jiao H., Hollmann D., Arndt P., Rosenthal U., Beweries T. (2017). Chem.–Eur. J..

[cit17] Pang Y., Petrich S. A., Young Jr V. G., Gordon M. S., Barton T. J. (1993). J. Am. Chem. Soc..

[cit18] Reiß F., Reiß M., Spannenberg A., Jiao H., Baumann W., Arndt P., Rosenthal U., Beweries T. (2018). Chem.–Eur. J..

[cit19] Trose M., Reiß M., Reiß F., Anke F., Spannenberg A., Boye S., Lederer A., Arndt P., Beweries T. (2018). Dalton Trans..

[cit20] Casey C. P., Kraft S., Powell D. R. (2000). J. Am. Chem. Soc..

[cit21] Shimizu T., Miyasaka D., Kamigata N. (2000). Org. Lett..

[cit22] For basic thermochemistry, molecular structures were optimized using the pure density functional (DF) BP86 in combination with the LANL2DZ basis set and corresponding ECP at Ti and the TZVP basis set on all other atoms (notation BP86/LANL2DZ/TZVP) (see ESI for details)

[cit23] Labella L., Chernega A., Green M. L. H. (1995). J. Chem. Soc., Dalton Trans..

[cit24] Pyykkö P., Atsumi M. (2009). Chem.–Eur. J..

[cit25] Becke A. D. (1988). Phys. Rev. A.

[cit26] Hay P. J., Wadt W. R. (1985). J. Chem. Phys..

[cit27] Schäfer A., Huber C., Ahlrichs R. (1994). J. Chem. Phys..

[cit28] Vosko S. H., Wilk L., Nusair M. (1980). Can. J. Phys..

[cit29] (b) Cramer C. J., Essentials of Computational Chemistry: Theories and Models, John Wiley & Sons, Ltd, Chichester, UK, 2004.

[cit30] Klene M., Robb M. A., Frisch M. J., Celani P. (2000). J. Chem. Phys..

[cit31] Salem L., Rowland C. (1972). Angew. Chem., Int. Ed. Engl..

[cit32] Where *β* = 1 indicates a “perfect” biradical with two electrons in two degenerate orbitals and smaller values indicate an increasing energy gap between HOMO and LUMO, and *β* → 0 indicates a closed-shell species

[cit33] Noodleman L. (1981). J. Chem. Phys..

[cit34] (c) Bader R. F. W., Atoms in Molecules: A Quantum Theory, Oxford University Press, 1994.

[cit35] Lu T., Chen F. (2012). J. Comput. Chem..

[cit36] Tonner R., Frenking G. (2007). Angew. Chem., Int. Ed..

[cit37] Hinz A., Kuzora R., Schulz A., Villinger A. (2014). Chem.–Eur. J..

[cit38] Hanna T. E., Lobkovsky E., Chirik P. J. (2007). Inorg. Chem..

[cit39] Smith M. R., Matsunaga P. T., Andersen R. A. (1993). J. Am. Chem. Soc..

[cit40] Suzuki N., Kurita R., Kawamura A., Ono T., Masuyama Y. (2018). J. Organomet. Chem..

[cit41] Chinchilla R., Nájera C. (2007). Chem. Rev..

[cit42] Doucet H., Hierso J. C. (2007). Angew. Chem., Int. Ed..

[cit43] Modern Carbonyl Olefination – Methods and Applications, ed. T. Takeda, Wiley-VCH, Weinheim, 2004.

[cit44] Hanna T. A., Baranger A. M., Walsh P. J., Bergman R. G. (1995). J. Am. Chem. Soc..

