# Supplementary material for: 1-Titanacyclobuta-2,3-diene – an elusive four-membered cyclic allene
Source: Chem Sci. 2019 Apr 23;10(20):5319–25. doi: 10.1039/c9sc01002e (PMC6540905; doi:10.1039/c9sc01002e)
Supplement: Supplementary file 1 [file SC-010-C9SC01002E-s001.pdf]

Electronic Supplementary Material (ESI) for Chemical Science.
This journal is © The Royal Society of Chemistry 2019

Supporting Information

1-Titanacyclobuta-2,3-diene – an Elusive Four-membered Cyclic Allene

Fabian Reiß*,[a] Melanie Reiß,[a] Jonas Bresien,[b] Anke Spannenberg,[a] Haijun Jiao,[a] Wolfgang Baumann,[a] Perdita Arndt,[a] and Torsten Beweries*[a]

[a] Leibniz-Institut für Katalyse e.V. an der Universität Rostock, Albert-Einstein-Str. 29a, 18059 Rostock, Germany.

[b] Abteilung für Anorganische Chemie, Institut für Chemie, Universität Rostock, Albert-Einstein-Straße 3a, D-18059 Rostock, Germany

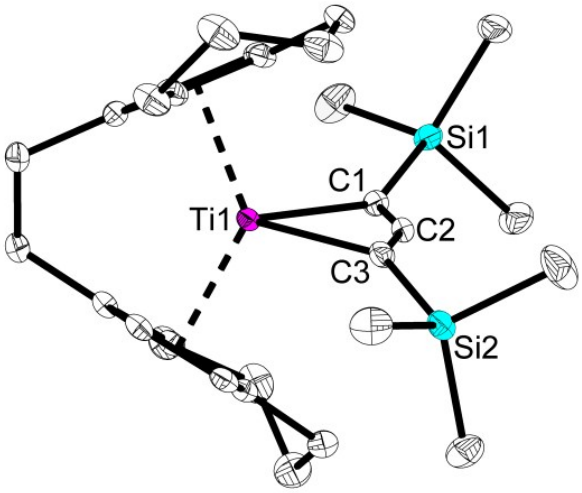

Content

1. Experimental Details 2
2. Reaction of 2 with ketones and aldehydes 5
3. Proof of Stability of Compound 2 11
4. Crystallographic Details 12
5. Details of the NMR spectroscopy 18
6. Details of vibrational spectroscopy 30
7. Computational Details 39
8. Literature 56

1. Experimental Details

1.1. General

All manipulations were carried out in an oxygen- and moisture-free argon atmosphere using standard Schlenk and drybox techniques. The solvents were purified with the Grubbs-type column system "Pure Solv MD-5" and dispensed into thick-walled glass Schlenk bombs equipped with Young-type Teflon valve stopcocks. Bis(cyclopentadienyl)titanium(IV) dichloride ([Cp_2TiCl_2], 97 %, Sigma-Aldrich) was recrystallised prior to used. Bis(pentamethylcyclopentadienyl)titanium(IV) dichloride ([$Cp^*_2TiCl_2$], MCAT) and *rac*-[1,2-bis-(4,5,6,7-tetra-hydro-inden-1-yl)ethan]titanium(IV) dichloride ([*rac*-(ebthi)$TiCl_2$], MCAT) were transferred in Schlenk Tubes stored under argon and used as received. [$Li_2(Me_3SiC_3SiMe_3)$] was prepared according to literature procedure and isolated as white solid.[1] Preparative chromatography was performed by elution from columns of slurry-packed Silica Gel 60 (0.04-0.063 mm, Macherey-Nagel GmbH). NMR spectra were determined on Bruker AV300 and AV400. ^{1}H and ^{13}C chemical shifts were referenced to the solvent signal: [D$_6$]benzene (δ_H 7.16, δ_C 128.06)[2], [D8]toluene(δ_H 2.08, δ_C 20.4) Accordingly, chemical shifts of ^{29}Si are given relative to $SiMe_4$, $\Xi(^{29}Si)$ = 19.867 187 MHz. Raman spectra were recorded on a LabRAM HR 800 Raman Horiba spectrometer equipped with an Olympus BX41 microscope with variable lenses was used. The samples were excited by different laser sources: 633 nm (17 mW, air cooled), 784 nm Laser diode (100 mW, air-cooled) or 473 nm Ar+ Laser (20 mW, air-cooled). All measurements were carried out at ambient temperature. IR spectra were recorded on a Bruker Alpha FT-IR, ATR Spectrometer, spectra are not corrected. MS analysis was done using a Finnigan MAT 95-XP (Thermo-Electron), CI^+/CI^- Isobutane and for the air stable compounds in EI mode. CHN analysis was done using a Leco Tru Spec elemental analyser. Melting points are uncorrected and were determined in sealed capillaries under Ar atmosphere using a Mettler-Toledo MP 70. Data were collected on a STOE IPDS II (**3**) and a Bruker Kappa APEX II Duo diffractometer (**2**), respectively. The structures were solved by direct methods (SHELXS-97)[3] and refined by full-matrix least-squares procedures on F^2 (SHELXL-2014).[4] XP (Bruker AXS) and Diamond[5] were used for graphical representations. All calculations were carried out with the Gaussian 09 package of molecular orbital programs.[6]

1.2. Reaction of $Cp^*_2TiCl_2$ with [$Li_2(Me_3SiC_3SiMe_3)$] to (E)-hexa-3-en-1,5-diyne-1,3,4,6-tetrayltetrakis(trimethylsilane) (1)

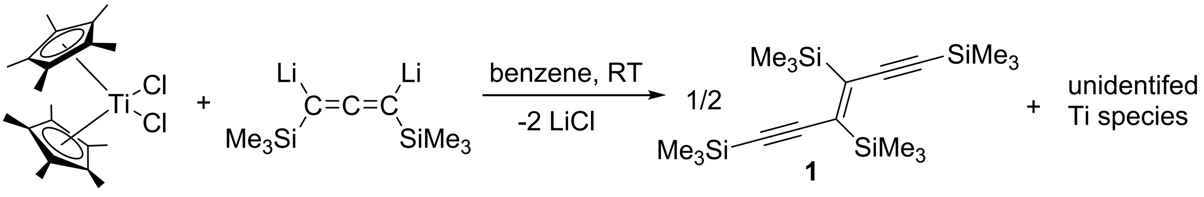

$Cp^*_2TiCl_2$ (0.51 mmol, 0.200 g) and [$Li_2(Me_3SiC_3SiMe_3)$] (0.51 mmol, 0.100 g) were mixed and dissolved in benzene (5 mL). After 12 hours of stirring at room temperature the solvent was removed in *vacuo*. Then the brown residue was extracted with pentane (5 x 4 mL) and cannula-filtered. Silica Gel was added to the combined pentane solutions. After removing the solvent the product **1** was separated by column chromatography (hexane). Yield 64 mg, 90 %.

m.p. 85–87 °C (air). **^{1}H NMR** (25 °C, [D$_6$]benzene, 400.13 MHz): δ = 0.47 (s, 18H, $^2J(^1H-^{29}Si)$ = 6.8 Hz, $^1J(^1H-^{13}C)$ = 120 Hz, 2 x $SiMe_3$-C3), 0.19 (s, 18H, $^2J(^1H-^{29}Si)$ = 7.0 Hz, $^1J(^1H-^{13}C)$ = 120, Hz, 2 x $SiMe_3$-

C1). **^{13}C NMR** (25 °C, [D_6]benzene, 100.61 MHz): δ = 150.5 (2 x C3), 111.5 (2 x C1), 109.0 (2 x C2), -0.2 (2 x $SiMe_3$-C1), -0.8 (2 x $SiMe_3$-C3). **^{29}Si-inept NMR** (25 °C, [D_6]benzene, 59.63 MHz): δ = –4.7 (dec, $^2J(^1H-^{29}Si)$ = 6.8 Hz, 2 x $SiMe_3$), –18.9 (dec, $^2J(^1H-^{29}Si)$ = 7.0 Hz, 2 x $SiMe_3$). **MS-CI$^+$** (*isobutane*): [M^+] 364 (23), [$M+H^+$] 365 (100), [$M-Me^+$] 349 (22). **IR** (ATR, 64 scans): 2955 (w), 2897 (w), 2121 (w), 1450 (w), 1242 (m), 1136 (w), 1105 (w), 830 (s), 754 (s), 730 (m), 696 (m), 636 (m), 618 (m), 532 (m), 449 (w). **RAMAN** (473 nm, 8 sec, 10 acc): 2959 (w), 2897 (w), 2099 (s), 2067 (w), 1462 (m), 1436 (w), 1257 (w), 1182 (w), 1140 (w), 755 (w), 721 (w), 694 (w), 632 (w), 615 (w), 586 (w), 385 (w). **Elemental analysis** calcd (%) for M($C_{18}H_{38}Si_4$) = 364.83 g mol^{-1}: C 58.93, H 10.44; found: C 58.21, H 10.23 (measured with V_2O_5. Without V_2O_5 C values decrease about 15 %).

1.3. Synthesis of 2

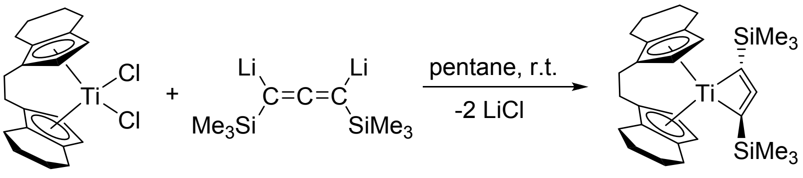

2

[*rac*-(ebthi)$TiCl_2$] (0.52 mmol, 200 mg) and [$Li_2(Me_3SiC_3SiMe_3)$] (0.52 mmol, 101 mg) were mixed and dissolved in pentane (10 mL) at 0 °C. Then the reaction mixture was slowly warmed to room temperature. After 12 hours of stirring at room temperature the deep red solution was cannula-filtered and the residue was extracted with pentane (5 x 3 mL). The combined pentane solutions were concentrated and stored at -78 °C for crystallisation to obtain complex **2** (150 mg, 58 %).

m.p. 108 °C (dec. Ar). **^{1}H NMR** (25 °C, [D_6]benzene, 400.13 MHz): δ = 7.22 (d, 2H, $^1J(^1H-^{13}C)$ = 172 Hz, $^3J(^1H-^1H)$ = 3.36 Hz, C*H* ebthi), 5.27 (d, 2H, $^1J(^1H-^{13}C)$ = 168 Hz, $^3J(^1H-^1H)$ = 3.36 Hz, C-*H* ebthi), 2.72–2.52 (m, 4H, 2 x C*H₂* ebthi), 2.41–2.25 (m, 2H, CH_2 ebthi), 2.05–1.87 (m, 4H, 2 x CH_2 ebthi), 1.43–1.07 (m, 10H, 5 x CH_2 ebthi), 0.35 (s, 18H, $^2J(^1H-^{29}Si)$ = 6.6 Hz, 2 x $SiMe_3$). **^{13}C NMR** (25 °C, [D_6]benzene, 100.61 MHz): δ = 213.8 (*C*=C=*C*), 134.2 (C=*C*=C), 124.1, 122.9, 117.2 (3 x *C* ebthi), 116.5, 100.2 (2 x *C*H ebthi), 27.4, 24.0, 23.9, 23.1, 22.8 (10 x CH_2 ebthi), 2.3 (2 x $SiMe_3$). **^{29}Si-inept NMR** (25 °C, [D_6]benzene, 59.63 MHz): δ = –11.28 (dec, $Si(CH_3)_3$, $^2J(^1H-^{29}Si)$ = 6.6 Hz). **MS-CI$^+$** (*isobutane*): [M^+] 494 (26), [$M-TMS^+$] 421 (21), [$(M \times 2)^+$] 988 (100). **IR** (ATR, 16 scans): 2924 (w), 2897 (w), 2849 (w), 1729 (m), 1692 (w), 1433 (w), 1344 (m), 1285 (w), 1240 (m), 1036 (w), 1001 (w), 952 (w), 828 (s), 777 (m), 750 (s), 715 (m), 683 (m), 626 (m), 604 (w), 518 (w), 502 (m), 408 (m). **RAMAN** (samples of **2** decompose while irradiation with all available laser sources). **Elemental analysis** calcd (%) for M($C_{29}H_{42}Si_2Ti$) = 494.69 g mol^{-1}: C 70.41, H 8.56; found: C 70.59, H 8.57. Single crystals suitable for single crystal diffraction were grown from pentane at room temperature.

1.4. Reaction of Cp_2TiCl_2 with $[Li_2(Me_3SiC_3SiMe_3)]$ at ambient temperature.

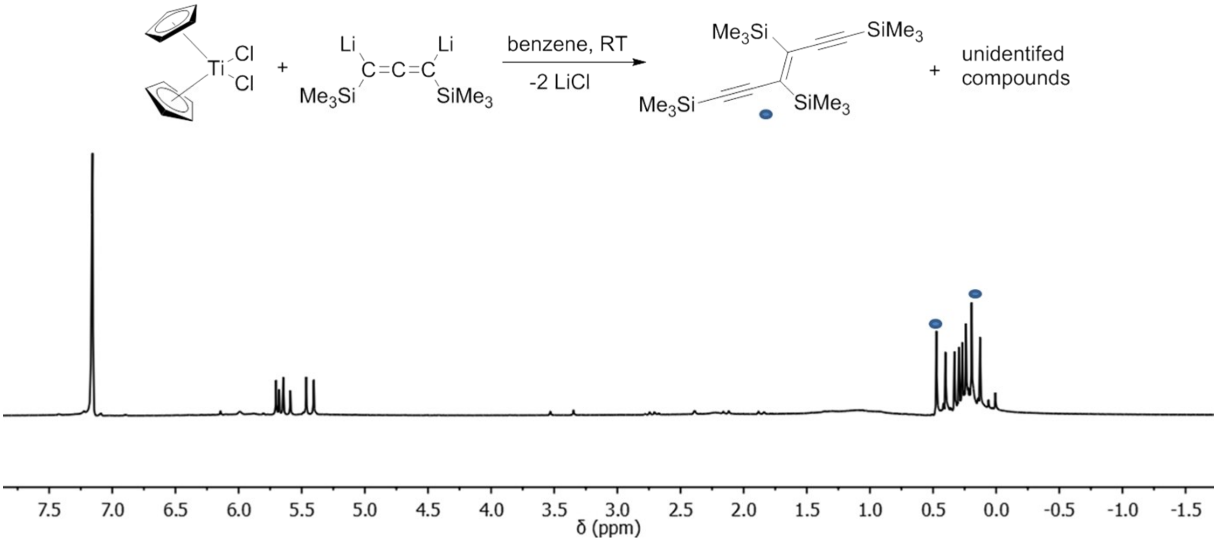

Figure S 1: ^1H NMR spectrum of the reaction mixture after 12 h (25 °C, [D$_6$]benzene, 300.20 MHz).

Cp_2TiCl_2 (0.51 mmol, 0.200 g) and $[Li_2(Me_3SiC_3SiMe_3)]$ (0.51 mmol, 0.100 g) were mixed and dissolved in benzene (5 mL). After 12 hours of stirring at room temperature an NMR sample was taken.

2. Reaction of 2 with ketones and aldehydes

2.1. Reaction of 2 with benzophenone to (4,4-diphenylbut-3-en-1-yne-1,3-diyl)bis(trimethylsilane) (3)

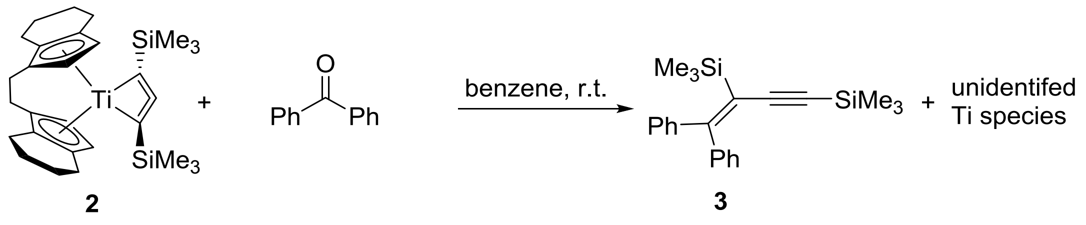

Compound **2** (0.20 mmol, 0.100 g) and benzophenone (0.20 mmol, 37 mg) were mixed and dissolved in benzene (2 mL). After 16 hours of stirring at room temperature the solvent was removed in *vacuo*. Then the brown residue was dissolved in pentane (5 mL) and Silica Gel was added. After removing the solvent the product **3** was separated by column chromatography (hexane/ethyl acetate 20 : 1). Yield 64 mg, 90 %.

m.p. 61–64 °C (in air). **^{1}H NMR** (25 °C, [D$_6$]benzene, 400.13 MHz): δ = 7.65 (m, 2H, Ph), 7.06 (m, 8H, Ph), 0.17 (s, 9H, $^{2}J(^{1}H-^{29}Si)$ = 7.0 Hz, SiMe$_3$), 0.12 (s, 9H, $^{2}J(^{1}H-^{29}Si)$ = 6.6 Hz, SiMe$_3$). **^{13}C NMR** (25 °C, [D$_6$]benzene, 100.61 MHz): δ = 162.8 (C4), 143.7, 142.9 (*i*-Ph), 130.4, 130.2 (Ph), 128–127 (4 signals of Ph under solvent signal), 123.2 (C3), 109.2 (C2), 101.7 (C1), 0.3 ((SiMe$_3$)-C3), 0.0 ((SiMe$_3$)-C1). **^{29}Si-inept NMR** (25 °C, [D$_6$]benzene, 59.63 MHz): δ = –4.08 (dec, Si(CH$_3$)$_3$, $^{2}J(^{1}H-^{29}Si)$ = 6.7 Hz), –19.23 (dec, Si(CH$_3$)$_3$, $^{2}J(^{1}H-^{29}Si)$ = 7.0 Hz). **MS-EI**: [M$^+$] 348 (100), [M$^+$-Me] 333 (42). **IR** (32 scans, ATR): 3077 (w), 3048 (w), 3024 (w), 2957 (w), 2923 (w), 2895 (w), 2855 (w), 2117 (m), 1567 (w), 1535 (w), 1486 (w), 1442 (w), 1405 (w), 1311 (w), 1291 (w), 1244 (m), 1179 (w), 1105 (w), 1071 (w), 1028 (w), 936 (w), 907 (w), 832 (s), 752 (s), 693 (s), 628 (m), 605 (m), 559 (w), 493 (m), 465 (w). **RAMAN** (473 nm, 8 sec, 10 acc): 3067 (m), 3054 (w), 2959 (w), 2897 (m), 2120 (m), 1597 (m), 1576 (w), 1534 (s), 1491 (w), 1409 (w), 1290 (w), 1176 (w), 1155 (w), 1106 (w), 1026 (w), 998 (m), 837 (w), 755 (w), 689 (w), 639 (w), 613 (w), 603 (w), 535 (w), 495 (w), 403 (w). **Elemental analysis** calcd (%) for M($C_{22}H_{28}Si_2$) = 348.17 g mol^{-1}: C 75.79, H 8.10; found: C 75.82, H 8.10. Single crystals suitable for single crystal diffraction were grown from pentane.

2.2. Reaction of 2 with acetone to (4-methylpent-3-en-1-yne-1,3-diyl)bis(trimethylsilane) (4)

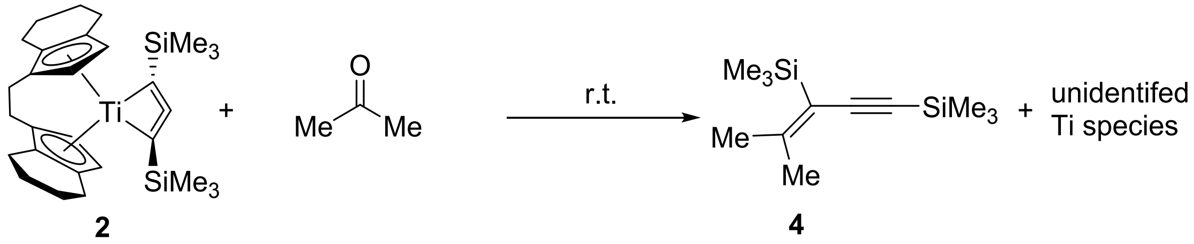

Compound **2** (0.30 mmol, 0.150 g) was dissolved in dry acetone (2 mL) at 0 °C. After 16 hours of stirring at room temperature, Silica Gel was added. After removing the solvent, the product **4** was separated by column chromatography (hexane) as colourless liquid. Yield 40 mg, 58 %.

^{1}**H NMR** (25 °C, [D$_6$]benzene, 400.13 MHz): δ = 2.03 (q, 3H, $^{4}J(^{1}H-^{1}H)$ = 0.38 Hz, $J(^{1}H-^{13}C)$ = 126 Hz CH$_3$), 1.63 (q, 3H, $^{4}J(^{1}H-^{1}H)$ = 0.38 Hz, $J(^{1}H-^{13}C)$ = 126 Hz, CH$_3$), 0.27 (s, 9H, $^{2}J(^{1}H-^{29}Si)$ = 6.4 Hz, $J(^{1}H-^{13}C)$ = 119 Hz, (SiMe$_3$)-C1), 0.25 (s, 9H, $^{2}J(^{1}H-^{29}Si)$ = 7.0 Hz, $J(^{1}H-^{13}C)$ = 120 Hz, (SiMe$_3$)-C3). ^{13}**C NMR** (25 °C, [D$_6$]benzene, 100.61 MHz): δ = 158.6 (C4), 118.4 (C3), 108.5 (C2), 99.3 (C1), 25.6, 24.0 (2 x CH$_3$), 0.6 ((SiMe$_3$)-C3), 0.3 ((SiMe$_3$)-C1). ^{29}**Si-inept NMR** (25 °C, [D$_6$]benzene, 59.63 MHz): δ = -8.1 (dec, Si(CH$_3$)$_3$, $^{2}J(^{1}H-^{29}Si)$ = 6.6 Hz), -19.8 (dec, Si(CH$_3$)$_3$, $^{2}J(^{1}H-^{29}Si)$ = 7.0 Hz). **MS-EI$^+$:** [M$^+$] 224 (6), [M-H$^+$] 223 (12), [M-C$_4$H$_9^+$] 167 (98), [M-SiMe$_3^+$] 152 (15), [SiMe$_3^+$] 73 (100). **IR** (32 scans, ATR): 2958 (w), 2899 (w), 2155 (w), 2117 (w), 1584 (w), 1444 (w), 1407 (w), 1368 (w), 1248 (m), 1128 (w), 1109 (w), 887 (w), 8334 (s), 756 (m), 693 (w), 634 (w), 614 (w), 455 cm^{-1} (w). **RAMAN** (784 nm, 8 sec, 10 acc): 2961 (w), 2898 (w), 2156 (w), 2117 (m), 1631 (w), 1586 (w), 1440 (w), 1411 (w), 1377 (w), 1242 (w), 1129 (w), 1065 (w), 840 (w), 758 (w), 696 (m), 633 (s), 612 (w), 602 (w), 512 (w), 455 (w), 432 cm^{-1} (w).

2.3. Reaction of 2 with acetophenone to *E*/*Z* isomere mixture of (4-phenylpent-3-en-1-yne-1,3-diyl)bis(trimethylsilane) (5*E* and 5*Z*)

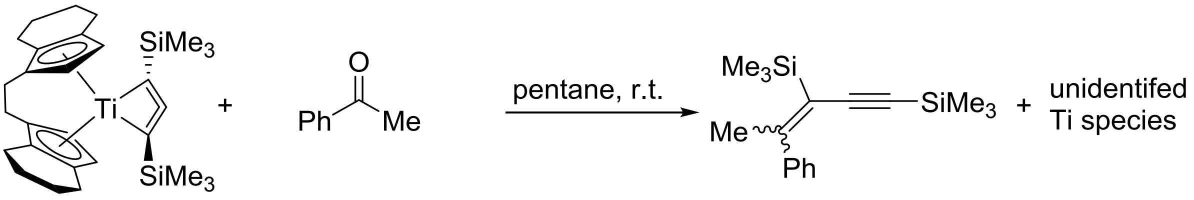

2 **E/Z isomere mixture** **5**

Compound **2** (0.20 mmol, 0.100 g) was dissolved in pentane (2 mL) and acetophenone (0.20 mmol, 24 mg) was added. After 16 hours of stirring at room temperature an NMR sample (E/Z ratio 0.8 : 1) was taken and then the solvent of the reaction mixture was removed in *vacuo*. Next, the orange residue was suspended in pentane (5 mL) and Silica Gel was added. After removing the solvent, the *E* and *Z* isomers (**5*E*** and **5*Z***) were separated by column chromatography (hexane/ethyl acetate 20 : 1). Yield: 13 mg (**5*E***), 13 mg (**5*Z***), 46 %. **MS-EI$^+$**: [M$^+$] 286 (58), [M-Me$^+$] 271 (59), [M-2Me$^+$] 255 (29), [TMS] 73 (72).

5*Z*: ^{1}**H NMR** (25 °C, [D$_6$]benzene, 400.13 MHz): δ = 7.01 (m, 5H, Ph), 2.40 (s, 3H, $J(^{1}H-^{13}C)$ = 127 Hz, CH$_3$ (C5)), 0.28 (s, 9H, $^{2}J(^{1}H-^{29}Si)$ = 6.6 Hz, $J(^{1}H-^{13}C)$ = 120 Hz, (SiMe$_3$)-C3), 0.05 (s, 9H, $^{2}J(^{1}H-^{29}Si)$ = 7.0 Hz, $J(^{1}H-^{13}C)$ = 120 Hz, (SiMe$_3$)-C1). ^{13}**C NMR** (25 °C, [D$_6$]benzene, 100.61 MHz): δ = 161.6 (C4), 144.8 (*i*-Ph), 128.3, 127.8, 127.7 (3 signals under solvent signal, Ph), 122.0 (C3), 108.2 (C2), 101.7 (C1), 26.8 (CH$_3$, (C5)), 0.5 ((SiMe$_3$)-C3), 0.2 ((SiMe$_3$)-C1). ^{29}**Si-inept NMR** (25 °C, [D$_6$]benzene, 59.63 MHz): δ = -6.0 (dec, Si(CH$_3$)$_3$, $^{2}J(^{1}H-^{29}Si)$ = 6.6 Hz), -19.4 (dec, Si(CH$_3$)$_3$, $^{2}J(^{1}H-^{29}Si)$ = 7.0 Hz). **RAMAN** (473 nm, 20 sec, 20 scans): 3061 (m), 2959 (m), 2848 (s), 2848 (w), 2201 (w), 2112 (s), 1600 (s), 1559 (s), 1467 (w), 1286 (w), 1137 (w), 1064 (w), 998 (m), 844 (w), 759 (w), 689 (w), 633 (m), 597 (w), 563 (w), 404 cm^{-1} (w).

5*E*: ^{1}**H NMR** (25 °C, [D$_6$]benzene, 400.13 MHz): δ = 7.52 (m, 2H, Ph), 7.21 (m, 2H, Ph), 7.09 (m, 1H, Ph), 2.03 (s, 3H, $J(^{1}H-^{13}C)$ = 127 Hz, CH$_3$ (C5)), 0.34 (s, 9H, $^{2}J(^{1}H-^{29}Si)$ = 6.8 Hz, $J(^{1}H-^{13}C)$ = 119 Hz, (SiMe$_3$)-C3), 0.11 (s, 9H, $^{2}J(^{1}H-^{29}Si)$ = 7.0 Hz, $J(^{1}H-^{13}C)$ = 120 Hz, (SiMe$_3$)-C1). ^{13}**C NMR** (25 °C,

[D$_6$]benzene, 100.61 MHz): δ = 159.4 (C4), 144.5 (*i*-Ph), 128.1, 127.8, 127.8 (3 signals under solvent signal, Ph), 120.2 (C3), 108.8 (C2), 99.5 (C1), 23.9 (CH$_3$, (C5)), 0.3 ((SiMe$_3$)-C3), 0.1 ((SiMe$_3$)-C1). **^{29}Si-inept NMR** (25 °C, [D$_6$]benzene, 59.63 MHz): δ = -6.7 (dec, Si(CH$_3$)$_3$, ^2J(^{1}H-^{29}Si) = 6.8 Hz), -19.7 (dec, Si(CH$_3$)$_3$, ^2J(^{1}H-^{29}Si) = 7.0 Hz). **RAMAN** (473 nm, 10 sec, 20 scans): 3067 (w), 2960 (m), 2900 (s), 2187 (w), 2115 (s), 1599 (m), 1578 (m), 1552 (s), 1439 (w), 1374 (w), 1287 (w), 1266 (w), 1183 (w), 1125 (w), 1027 (w), 999 (s), 837 (w), 758 (w), 692 (w), 636 (m), 613 (w), 572 (w), 402 cm^{-1} (w).

2.4. Reaction of 2 with benzaldehyde to *E/Z* isomere mixture of (4-phenylbut-3-en-1-yne-1,3-diyl)bis(trimethylsilane) (6*E* and 6*Z*)

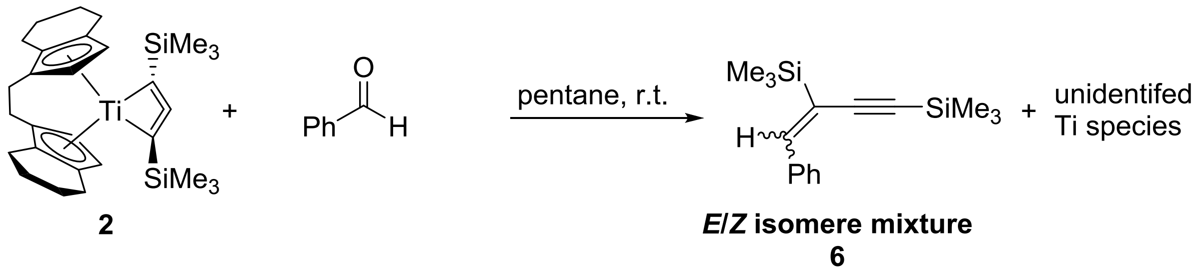

Compound **2** (0.20 mmol, 0.100 g) was dissolved in pentane (2 mL) and benzaldehyde (0.20 mmol, 22 mg) was added. After 16 hours of stirring at room temperature the solvent of the reaction mixture was removed in *vacuo*. Next, the orange residue was suspended in pentane (5 mL) and was filtered over Silica Gel. After removing the solvent, a mixture of *E/Z* isomers (**6**, *E/Z* ratio 0.8 : 1) was separated as colourless liquid. Yield: 27 mg, 50 %,.

^{1}H NMR (25 °C, [D$_6$]benzene, 400.13 MHz): δ = 8.11 (m, 2H, Ph), 7.89 (s, 1H, *H*-C4[Z]), 7.22 (m, 2H, Ph), 7.04 (m, 6H, Ph), 6.88 (s, 1H, *H*-C4[E]), 0.28 (s, 9H, *J*(^{1}H-^{13}C) = 120 Hz, (SiMe$_3$)-C1[Z]), 0.26 (s, 9H, *J*(^{1}H-^{13}C) = 120 Hz, (SiMe$_3$)-C3[E]), 0.24 (s, 9H, *J*(^{1}H-^{13}C) = 120 Hz, (SiMe$_3$)-C1[E]), 0.17 (s, 9H, *J*(^{1}H-^{13}C) = 120 Hz, (SiMe$_3$)-C3[Z]). **^{13}C NMR** (25 °C, [D$_6$]benzene, 100.61 MHz): δ = 152.9 (C4[Z]), 145.8 (C4[E]), 139.0 (*i*-Ph[Z]), 138.3 (*i*-Ph[E]), 129.4, 129.0, 128.7, 128.4, 128.2, 128.2 (2 x Ph), 127.6 (C3[Z]), 124.2 (C3[E]), 110.2 (C2[Z]), 107.2 (C1[E]), 106.9 (C2[E]), 97.1 (C1[Z]), 0.3 (SiMe$_3$)-C1[Z]), 0.2 ((SiMe$_3$)-C3[Z]), 0.1 (SiMe$_3$)-C1[E]), -1.9 ((SiMe$_3$)-C3[E]). **^{29}Si-inept NMR** (25 °C, [D$_6$]benzene, 59.63 MHz): δ = -0.7 (dec, ^2J(^{1}H-^{29}Si) = 6.8 Hz, Si(CH$_3$)$_3$-C3[E]), -5.9 (m, Si(CH$_3$)$_3$-C3[Z]), -18.7 (m, Si(CH$_3$)$_3$), -19.1 (m, Si(CH$_3$)$_3$). **MS-EI$^+$**: [M$^+$] 272 (75), [M-Me$^+$] 257 (79), [M-2Me$^+$] 242 (17), [TMS] 73 (95). **IR** of **6** isomer mixture (32 scans, ATR): 3059 (w), 3026 (w), 2959 (w), 2897 (w), 2117 (m), 1584 (w), 1558 (w), 1491 (w), 1446 (w), 1407 (w), 1248 (m), 1111 (w), 1062 (w), 1028 (w), 922 (w), 858 (m), 832 (s), 752 (m), 689 (m), 634 (w), 602 (w), 589 (w), 567 (w), 524 cm^{-1} (w). **Raman** of **6** isomer mixture (784 nm, 15 sec, 15 acc): 3059 (w), 2964 (w), 2899 (w), 2118 (m), 1600 (m), 1587 (w), 1558 (m), 1446 (w), 1362 (w), 1206 (w), 1186 (w), 1156 (w), 1111 (w), 1028 (w), 1000 (s), 922 (w), 882 (w), 842 (w), 757 (w), 695 (w), 634 (w), 589 (w), 567 (w), 486 cm^{-1} (w).

2.5. Reaction of 2 with acetone at low temperature to characterise the intermediate structure 7

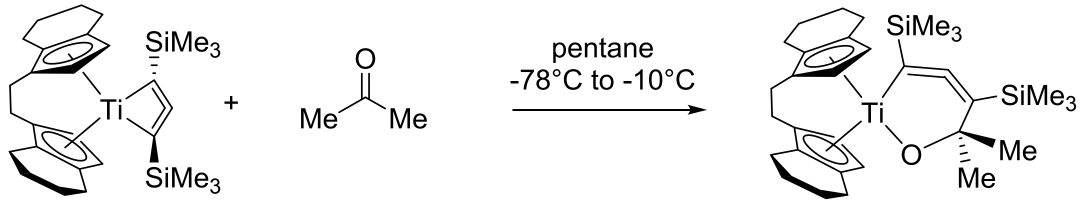

2 **7**

Compound **2** (0.20 mmol, 0.100 g) was dissolved in pentane (8 mL) at ambient temperature and then cooled to -78 °C. To this solution, neat acetone (0.2 mmol, 0.012 g) was added at this temperature. The temperature was slowly raised to -15°C, where a colour gradient from red to petrol was obtained within 4 hours. This turbid reaction mixture was dried *in vacuo* at -20 °C for 4 h, the residue was extracted/filtered with pentane (2 mL) at -20 °C. This filtrate was concentrated to approximately 1 mL and was slow cooled to -78 °C. The resulting dark petrol coloured residue was identified as **7** by low temperature NMR and IR spectroscopy. This complex is only stable at temperatures below -10 °C.

^{1}H NMR (-10 °C, [D$_8$]toluene, 400.13 MHz): δ = 6.83 (d, 1H, $^{1}J(^{1}H\text{-}^{13}C)$ = 170 Hz, $^{3}J(^{1}H\text{-}^{1}H)$ = 3.0 Hz, C-*H* ebthi), 6.73 (d, 1H, $^{1}J(^{1}H\text{-}^{13}C)$ = 170 Hz, $^{3}J(^{1}H\text{-}^{1}H)$ = 2.6 Hz, C-*H* ebthi), 5.13 (d, 1H, $^{1}J(^{1}H\text{-}^{13}C)$ = 170 Hz, $^{3}J(^{1}H\text{-}^{1}H)$ = 3.0 Hz, C-*H* ebthi), 4.90 (d, 1H, $^{1}J(^{1}H\text{-}^{13}C)$ = 170 Hz, $^{3}J(^{1}H\text{-}^{1}H)$ = 2.6 Hz, C-*H* ebthi), 3.04 (m, 1H, C*H$_2$* ebthi), 2.83 (m, 1H, C*H$_2$* ebthi), 2.40 (m, 10H, 5 x C*H$_2$* ebthi), 1.67 (m, 8H, 4 x C*H$_2$* ebthi), 1.44 (br. s, 6H, $J(^{1}H\text{-}^{13}C)$ = 125 Hz, CH_3), 0.40 (s, 9H, $^{2}J(^{1}H\text{-}^{29}Si)$ = 6.5 Hz, $J(^{1}H\text{-}^{13}C)$ = 119 Hz, (C1-Si*Me*$_3$)), 0.32 (s, 9H, $^{2}J(^{1}H\text{-}^{29}Si)$ = 6.5 Hz, $J(^{1}H\text{-}^{13}C)$ = 119 Hz, (C3-Si*Me*$_3$)). **^{13}C NMR** (-10 °C, [D$_8$]toluene, 100.61 MHz): δ = 179.7 (C2), 151.9 (C1), 139.1, 131.7, 131.3 129.4, 123.0, 119.5 (6 x *C* ebthi), 109.6, 107.7, 105.1, 104.7 (4 x *C*-H ebthi), 109.4 (C3), 90.3 (C4), 38.9, 31.7 (2 x C4-*Me*), 29.3, 28.6, 26.0, 25.9, 25.1, 25.0, 24.0, 23.6, 23.3, 23.0 (10 x *C*H$_2$ ebthi), 3.1 (C1-Si*Me*$_3$), 2.0 (C3-Si*Me*$_3$). **^{29}Si-inept NMR** (-10 °C, [D$_8$]toluene, 59.63 MHz): δ = -7.17 (dec, $Si(CH_3)_3$, $^{2}J(^{1}H\text{-}^{29}Si)$ = 6.4 Hz), -12.6 (dec, $Si(CH_3)_3$, $^{2}J(^{1}H\text{-}^{29}Si)$ = 6.4 Hz). **IR** (32 scans): 2955 (m), 2924 (m), 2855 (w), 1805 (m), 1446 (w), 1244 (m), 1177 (w), 932 (m), 828 (s), 781 (s), 559 (m).

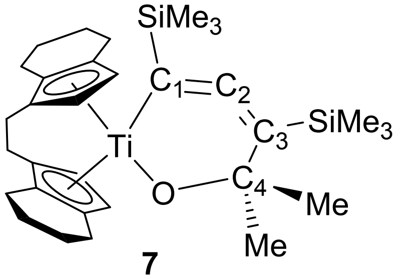

7

Scheme S1: NMR Assignment Scheme of **7**.

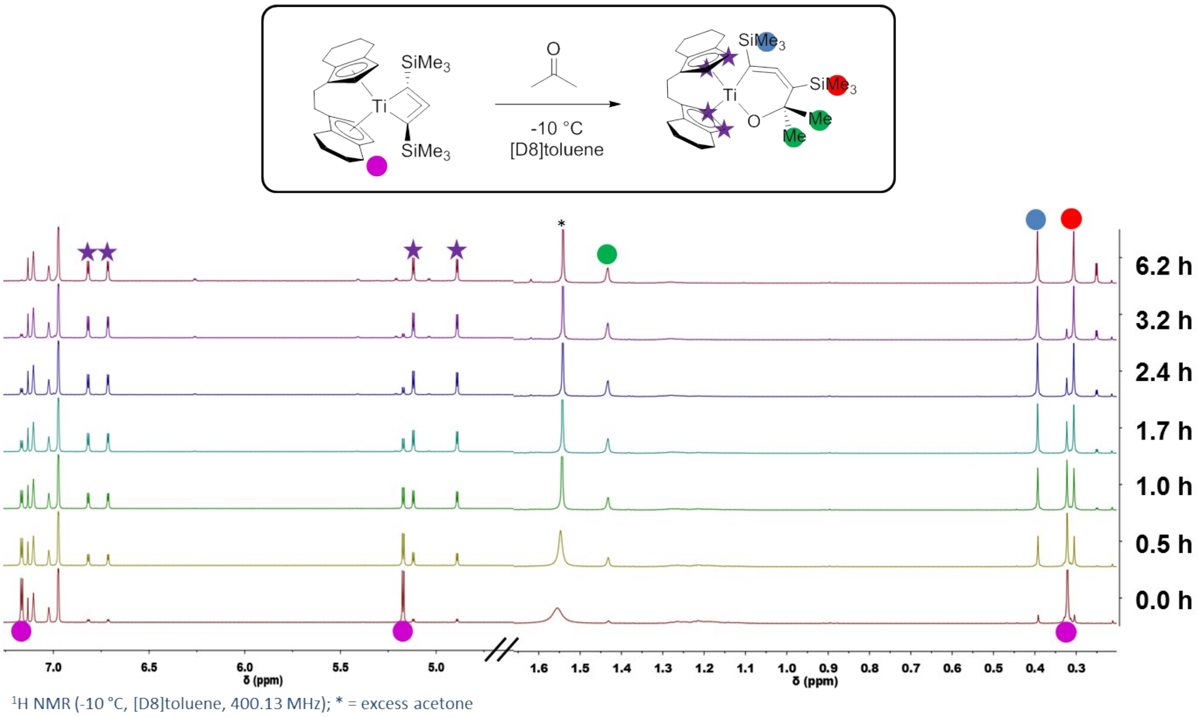

Figure S2: Low-temperature reaction monitoring of the formation of **7** at -10 °C in [D_8]toluene (c.f. Figure S20, Figure S21).

2.6. Reaction of 2 with benzaldehyde at low temperature to characterise the proposed intermediate structure 8.

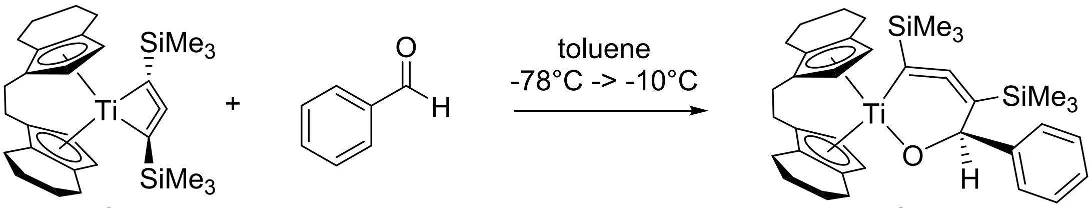

2 **8**

Compound **2** (0.05 mmol, 0.025 g) was dissolved in [D_8]toluene (0.7 mL) at ambient temperature and then cooled to -78 °C. To this solution, neat benzaldehyde (0.05 mmol, 0.005 g) was added at this temperature. The sample was brought to –10 °C in the probe of the NMR spectrometer and the reaction sequence was monitored at that temperature for 6 hours while recording a series of NMR spectra. The conversion proved slow enough to characterise the intermediate **8**.

^{1}H NMR (-10 °C, [D_8]toluene, 400.13 MHz): δ = 6.78 (d, 1H, $^3J(^1H-^1H)$ = 2.7 Hz, C-*H* ebthi), 6.10 (s, 1H C4-*H*), 5.25 (d, 1H, $^3J(^1H-^1H)$ = 3.2 Hz, C-*H* ebthi), 4.82 (d, 1H, $^3J(^1H-^1H)$ = 2.7 Hz, C-*H* ebthi), 3.55 (m, 4H, C*H$_2$* ebthi), 0.46 (s, 9, $J(^1H-^{13}C)$ = 119 Hz, (C1-Si*Me*$_3$)), 0.09 (s, 9H, $^2J(^1H-^{29}Si)$ = 6.6 Hz, $J(^1H-^{13}C)$ = 118 Hz, (C3-Si*Me*$_3$)). **^{13}C NMR** (-10 °C, [D_8]toluene, 100.61 MHz): δ = 179.5 (C2), 154.2 (C1), 149.98 (*ipso*-C_{Ph}), 137.8, 130.9, 128.9, 124.2, 120.2,(5 x Ph) 111.1, 104.3, 103.5, 104.7 (4 x *C*-H ebthi), 109.1 (C3), 91.8 (C4), 2.51 (C1-Si*Me*$_3$), -0.2 (C3-Si*Me*$_3$). **^{29}Si-inept NMR** (-10 °C, [D_8]toluene, 59.63 MHz): δ = -6.58 (m, C1-Si(CH$_3$)$_3$,), -10.3 (m, C3-Si(CH$_3$)$_3$,). (A more precise assignment was not made, since the signals in the reaction mixture cannot be assigned without any doubt.)

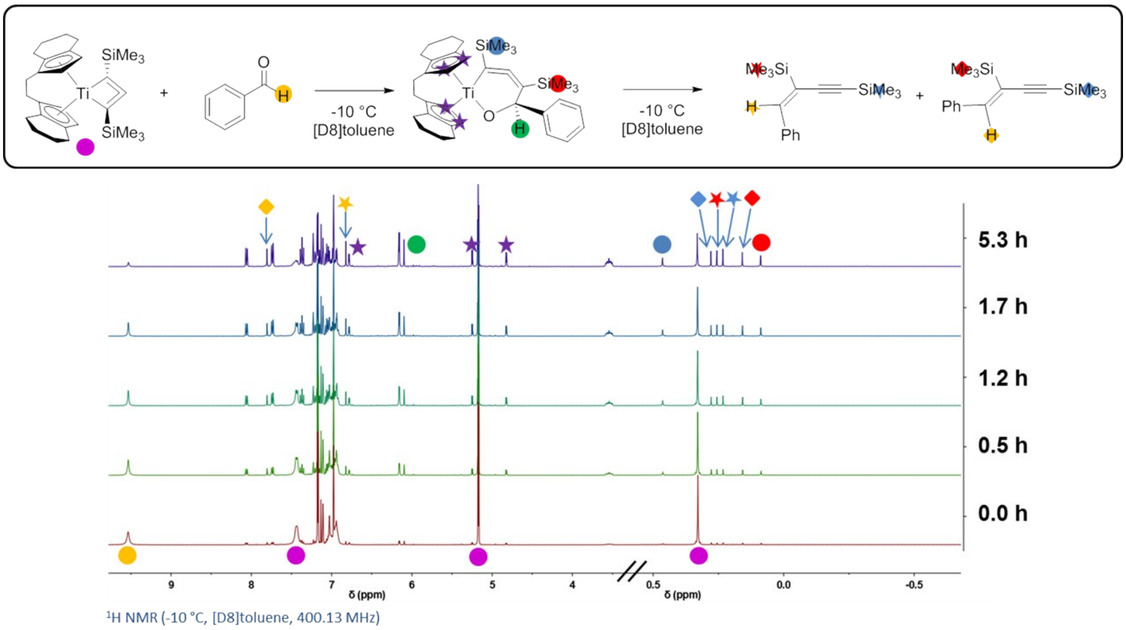

Figure S3: Low-temperature reaction monitoring of the formation of **8** at -10 °C in $[D_8]$toluene.

3. Proof of Stability of Compound 2

3.1. Decomposition of 2 in air and water

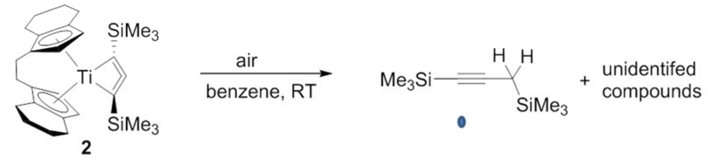

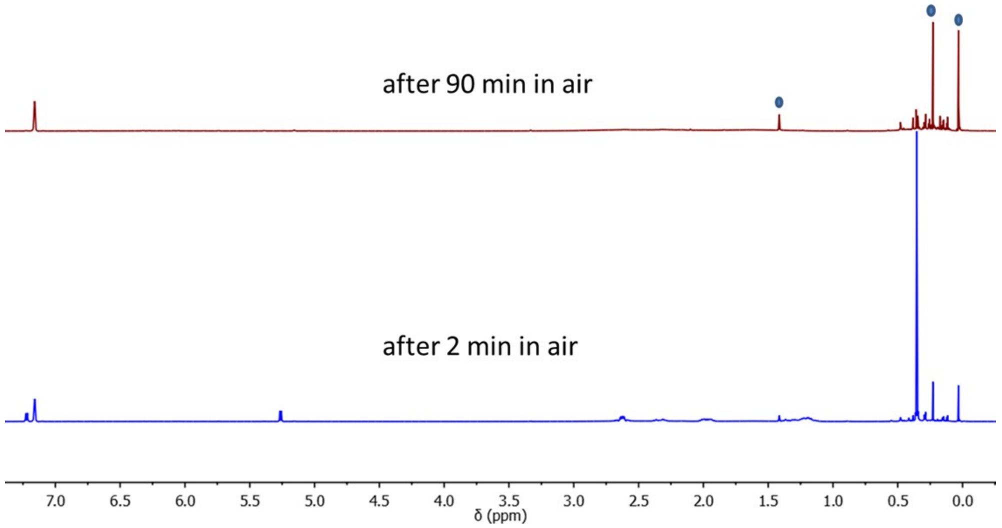

Figure S4: ^{1}H NMR spectrum of **2** in [D$_6$]benzene after exposure to air for 2 min and 90 min.

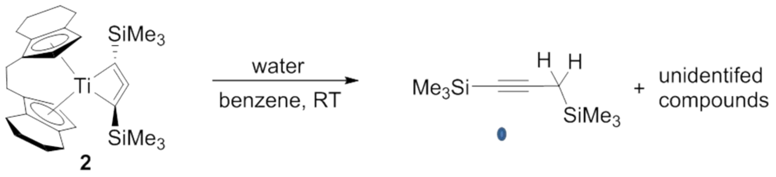

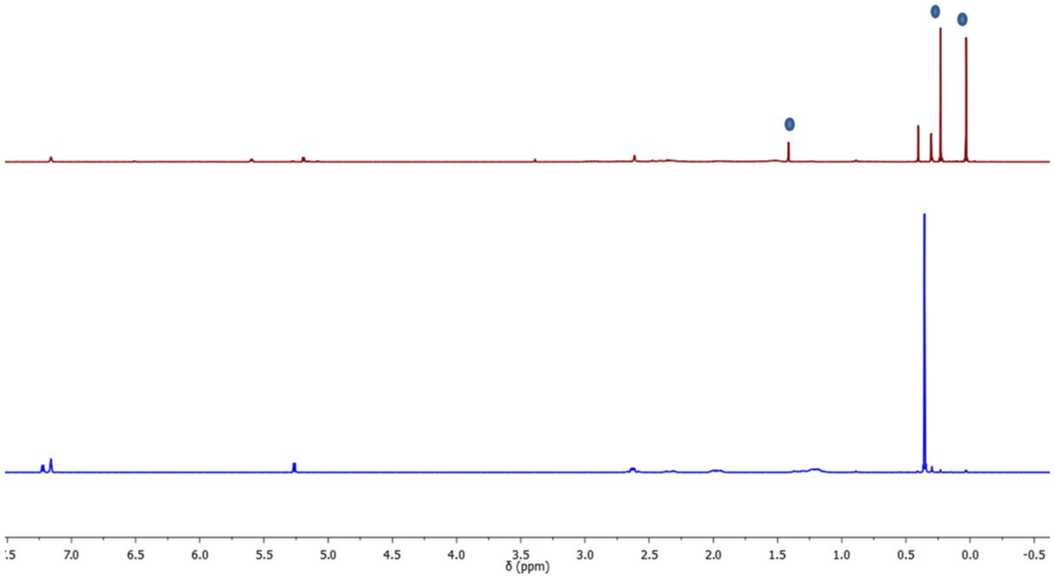

Figure S5: ^{1}H NMR spectrum **2** in [D$_6$]benzene after exposure to water.

4. Crystallographic Details

Table S1: Crystallographic details of **2** and **3**.

	**2**	**3**
Chem. Formula	$C_{29}H_{42}Si_2Ti$	$C_{22}H_{28}Si_2$
Form. Wght [g mol^{-1}]	494.70	348.62
Colour	red	colourless
Cryst. system	monoclinic	monoclinic
Space group	$P2_1/n$	$P2_1/n$
a [Å]	14.2569(6)	11.485(2)
b [Å]	10.8620(4)	9.6273(19)
c [Å]	18.2070(7)	19.737(4)
α [°]	90	90
β [°]	98.8260(8)	101.59(3)
γ [°]	90	90
V [$Å^3$]	2786.12(19)	2137.7(8)
Z	4	4
$\rho_{calc.}$ [g cm^{-3}]	1.179	1.083
μ [mm^{-1}]	0.408	0.167
T [K]	150(2)	150(2)
radiation type	MoKα	MoKα
reflections measured	25163	36201
independent reflections	6735	5154
observed reflections with I > 2σ(I)	5551	3064
$R_{int.}$	0.0286	0.0688
F(000)	1064	752
R_1 ($I > 2\sigma(I)$])	0.0340	0.0338
wR_2 (all data)	0.0934	0.0852
GOF on F^2	1.013	0.814
Parameters	314	223
CCDC number	1897219	1897220

4.1. Compound 2

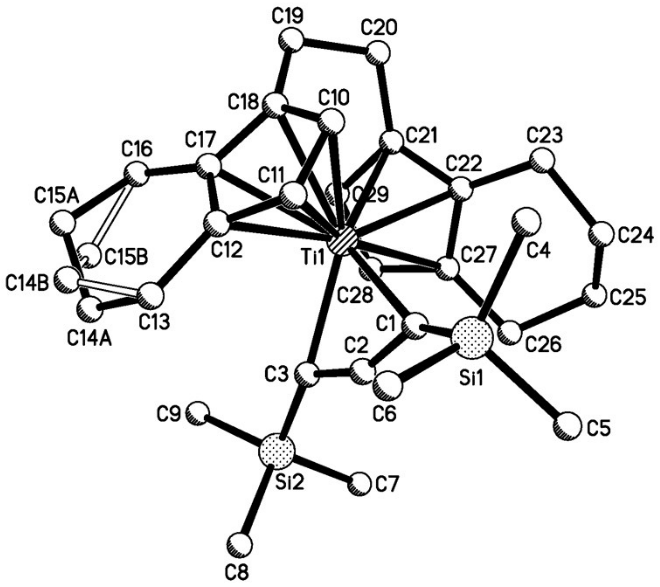

Figure S6: Numbering scheme of **2**. H atoms are omitted for clarity.

Table S2: Selected bond lengths (Å), angles and torsion angles (°) of **2**.

**Ti1–C1**	2.2287(14)	**C2–C1–Si1**	136.71(12)
**Ti1–C2**	2.1781(14)	**C2–C1–Ti1**	70.69(9)
**Ti1–C3**	2.2349(15)	**Si1–C1–Ti1**	145.29(8)
**C1–C2**	1.303(2)	**C1–C2–C3**	150.08(15)
**C2–C3**	1.308(2)	**C2–C3–Ti1**	70.39(9)
**C1–Si1**	1.8370(15)	**C2–C3–Si2**	134.80(13)
**C3–Si2**	1.8326(16)	**Si2–C3–Ti1**	148.47(8)
		**C1–Ti1–C3**	68.83(6)

4.2. Compound 3

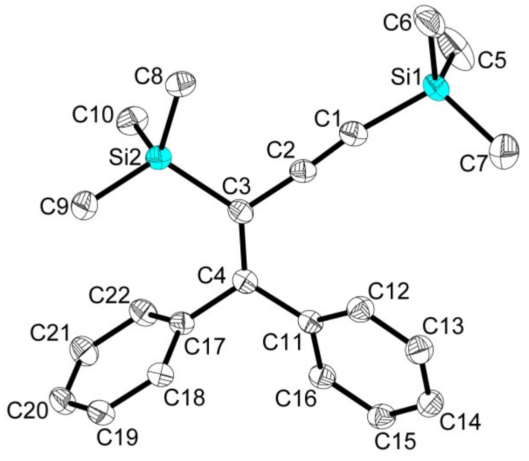

Figure S7: Molecular structure of compound **3**. Thermal ellipsoids correspond to 30% probability. Hydrogen atoms are omitted for clarity.

Table S3: Selected bond lengths (Å), angles and torsion angles (°) of **3**.

**C1–Si1**	1.8276(16)	**C2–C1–Si1**	172.62(13)
**C1–C2**	1.2080(19)	**C1–C2–C3**	176.07(15)
**C2–C3**	1.4364(19)	**C2–C3–Si2**	113.09(9)
**C3–Si2**	1.9002(15)	**C4–C3–Si2**	127.25(11)
**C3–C4**	1.3595(18)	**C4–C3–C2**	119.63(13)
**C4–C11**	1.485(2)	**C11–C4–C17**	114.94(11)
**C4–C17**	1.4881(19)	**C3–C4–C11**	124.65(12)
**C2–C3–C4–C11**	7.7(2)	**C3–C4–C17**	120.42(12)
**C2–C3–C4–C17**	-172.18(12)		

4.3. Comparision of structural features of 2 with known metallacyclobutadienes

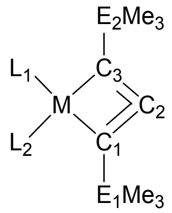

Table S4: Comparision of known structural parameters from known complexes and **2.**

Compound	M-C_1	M-C_2	M-C_3	C_1-C_2	C_2-C_3	C_1-E_1	C_3-E_2	C_1-M-C_3	C_1-C_2-C_3	$\angle\sum(C_1)$	$\angle\sum(C_3)$
rac-(ebthi)Ti($Me_3SiC_3SiMe_3$)<br>M = Ti, E = Si	2.2287(14)	2.1781(14)	2.2349(15)	1.303(2)	1.308(2)	1.8370(15)	1.8326(16)	68.83(6)	150.08(15)	352.69	353.66
Cp(Cl)W($Me_3CC_3CMe_3$) (**B**)[7]<br>M = W, E = C	1.929(4)	2.049(2)	1.919(2)	1.311(1)	1.399(2)	1.565(2)	1.501(2)	79.4	130.2	359.79	358.36
$(Py)_2(OC(CF_3)_2)_2Mo(Me_3CC_3CMe_3)$ (**A**)[8]<br>M = Mo, E = C	1.943(3)	2.005(4)	1.943(3)	1.379(4)	1.379(4)	1.519(4)	1.519(4)	81.7	134.4	359.98	359.98
$(Ph_3SiO)_2$(phen)Mo(RC_3R) (**C**)[9]<br>M = Mo, R = *p*-$MeOC_6H_4$	1.961(5)	2.030(5)	1.979(4)	1.371(7)	1.374(6)	1.456(6)	1.457(6)	80.20(1<br>9)	135.2(4)	359.9	358.99

Table S5: Bond analysis (J. March) with the literature values and structural parameters of **2.[a]**

Compound	C_1-C_2	C_2-C_3	C_1-E_1	C_3-E_2
rac-(ebthi)Ti($Me_3SiC_3SiMe_3$)<br>M = Ti, E = Si	$C_{sp2} = C_{sp}$<br>$C_{sp} = C_{sp}$	$C_{sp2} = C_{sp}$<br>$C_{sp} = C_{sp}$	-	-
Cp(Cl)W($Me_3CC_3CMe_3$) (**B**)[7]<br>M = W, E = C	$C_{sp2} = C_{sp2}$<br>$C_{sp2} = C_{sp}$	$C_{sp} – C_{sp}$<br>$C_{sp2} – C_{sp}$	$C_{sp3} – C_{sp3}$<br>$C_{sp3} – C_{sp2}$	$C_{sp3} – C_{sp3}$
$(Py)_2(OC(CF_3)_2)_2Mo(Me_3CC_3CMe_3)$ (**A**)[8]<br>M = Mo, E = C	$C_{sp} – C_{sp}$<br>$C_{sp2} – C_{sp}$	$C_{sp} – C_{sp}$<br>$C_{sp2} – C_{sp}$	$C_{sp3} – C_{sp3}$<br>$C_{sp3} – C_{sp2}$	$C_{sp3} – C_{sp3}$<br>$C_{sp3} – C_{sp2}$
$(Ph_3SiO)_2$(phen)Mo(RC_3R) (**C**)[9]<br>M = Mo, R = *p*-$MeOC_6H_4$	$C_{sp} – C_{sp}$<br>$C_{sp2} – C_{sp}$	$C_{sp} – C_{sp}$<br>$C_{sp2} – C_{sp}$	$C_{sp3} – C_{sp2}$<br>$C_{sp2} – C_{sp}$	$C_{sp3} – C_{sp2}$<br>$C_{sp2} – C_{sp}$

[a] = two best fitting descriptions are presented: Values taken from literature[10] ($C_{sp3} – C_{sp3}$ 1.54; $C_{sp3} – C_{sp2}$ 1.50; $C_{sp2} – C_{sp}$ 1.42; $C_{sp} – C_{sp}$ 1.38; $C_{sp2} = C_{sp2}$ 1.34; C $C_{sp2} = C_{sp}$ 1.31; C sp = C sp 1.28

Table S 6: Bond analysis with respect to the reported literature values (P. Pyykkö).[a]

Compound	C_1-C_2	C_2-C_3	C_1-E_1	C_3-E_2
rac(EBTHI)Ti($Me_3SiC_3SiMe_3$)<br>M = Ti, E = Si	C=C<br>C≡C	C=C<br>C≡C	C-Si<br>C=Si	C-Si<br>C=Si
Cp(Cl)W($Me_3CC_3CMe_3$) (**B**)[7]<br>M = W, E = C	C=C<br>C≡C	C-C<br>C=C	C–C	C–C
$(Py)_2(OC(CF_3)_2)_2Mo(Me_3CC_3CMe_3)$ (**A**)[8]<br>M = Mo, E = C	C-C<br>C=C	C-C<br>C=C	C–C	C–C
$(Ph_3SiO)_2$(phen)Mo(RC_3R) (**C**)[9]<br>M = Mo, R = *p*-$MeOC_6H_4$	C-C<br>C=C	C-C<br>C=C	C–C<br>C=C	C–C<br>C=C

[a] = two best fitting descriptions are presented: Values taken from literature[11] C–C = 1.50; C=C = 1.34; C≡C = 1.20; C-Si = 1.91; C=Si = 1.74.

5. Details of the NMR spectroscopy

5.1. ^{1}H and ^{13}C NMR spectra of 1.

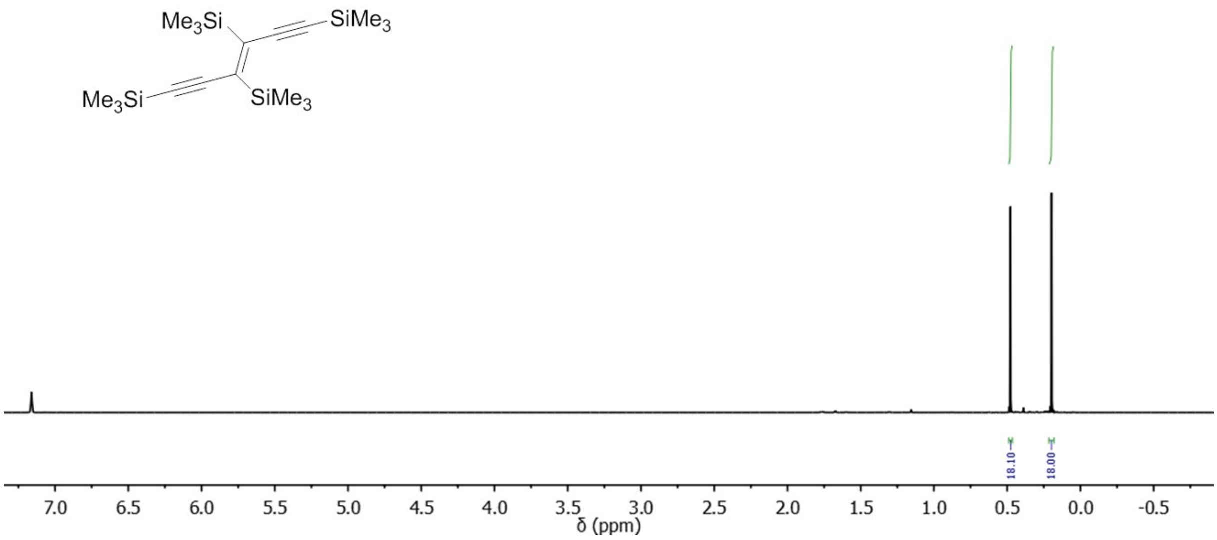

Figure S8: ^{1}H NMR spectrum of **1** (25 °C, [D$_6$]benzene, 400.13 MHz).

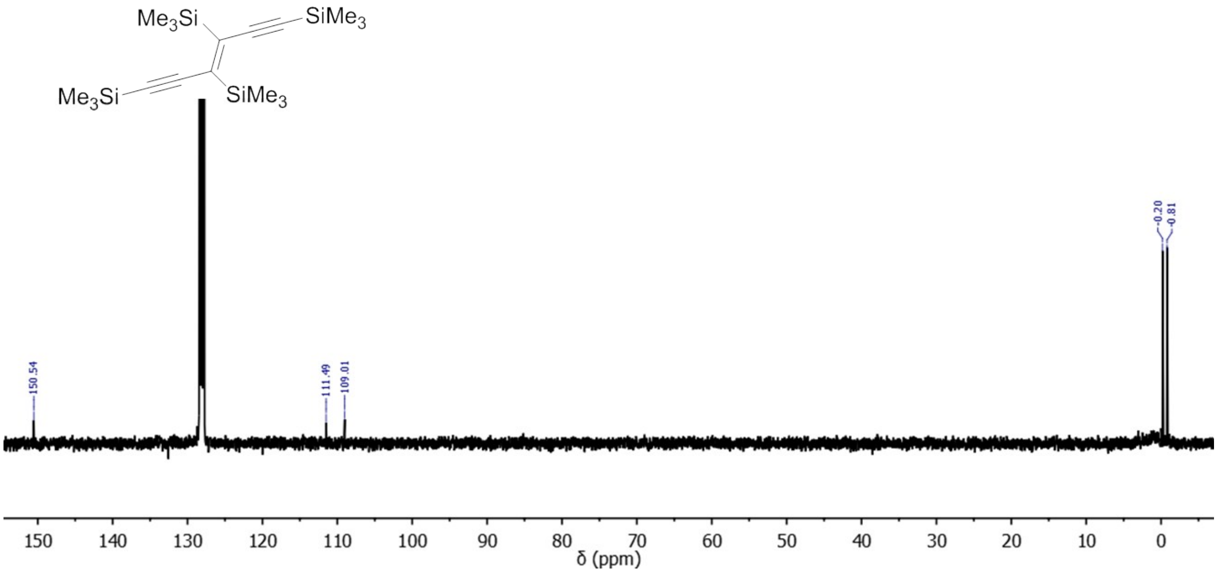

Figure S9: ^{13}C{^{1}H} NMR spectrum of **1** (25 °C, [D$_6$]benzene, 100.61 MHz).

5.2. ^{1}H and ^{13}C NMR spectra of 2

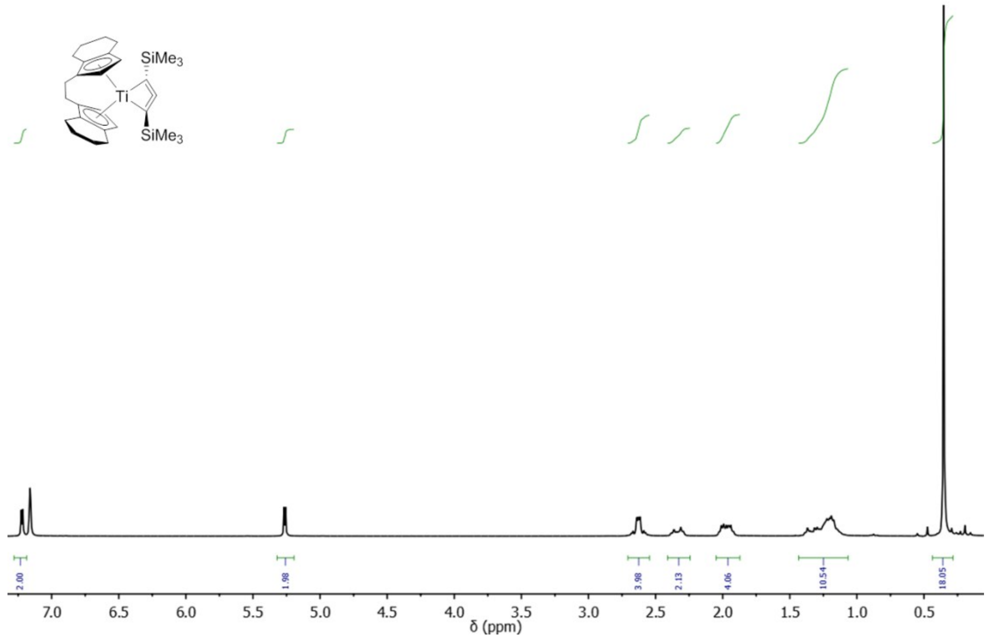

Figure S10: ^{1}H NMR spectrum of **2** (25 °C, [D_6]benzene, 400.13 MHz).

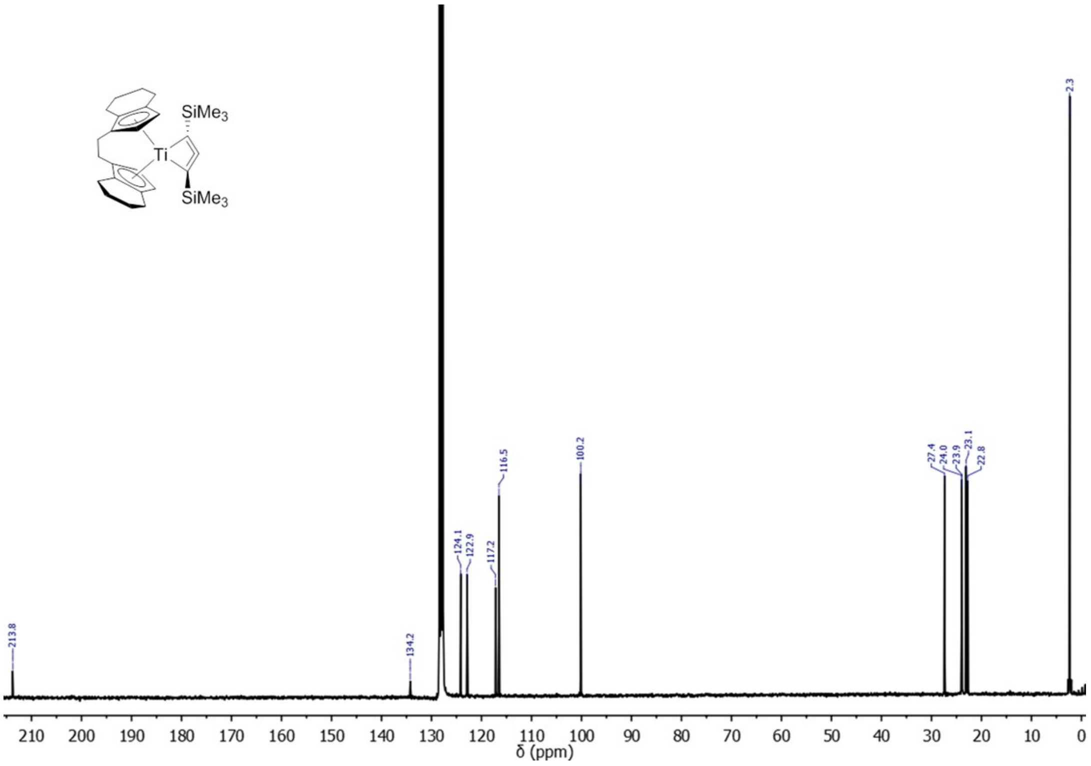

Figure S11: $^{13}C\{^{1}H\}$ NMR spectrum of **2** (25 °C, [D_6]benzene, 100.61 MHz).

5.3. ^{1}H and ^{13}C NMR spectra of 3

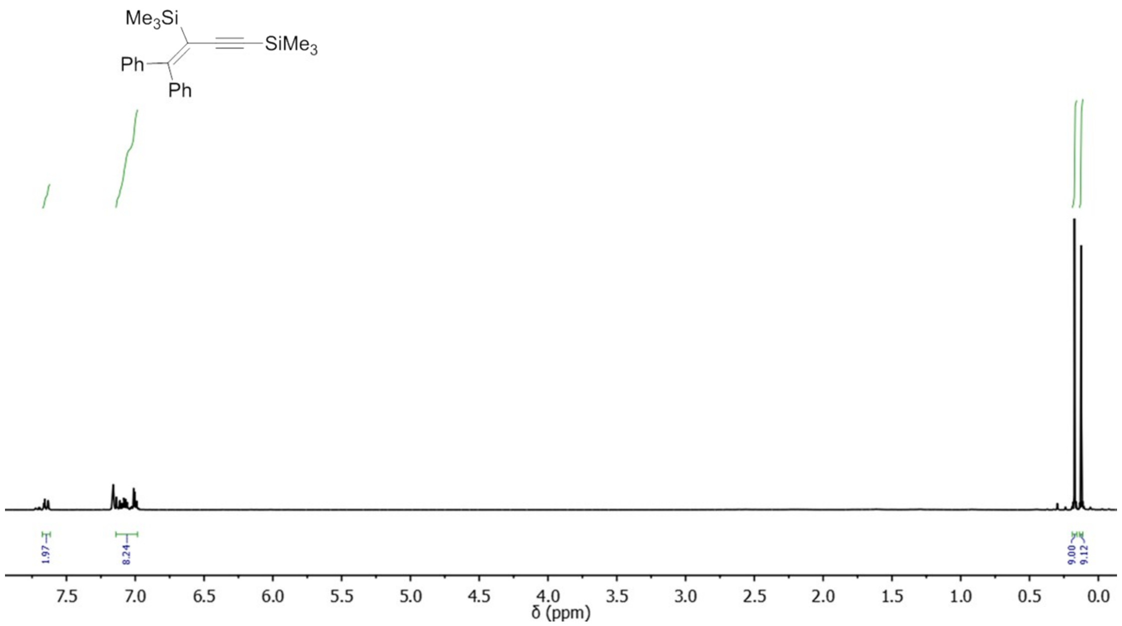

Figure S12: ^{1}H NMR spectrum of **3** (25 °C, [D$_6$]benzene, 300.20 MHz).

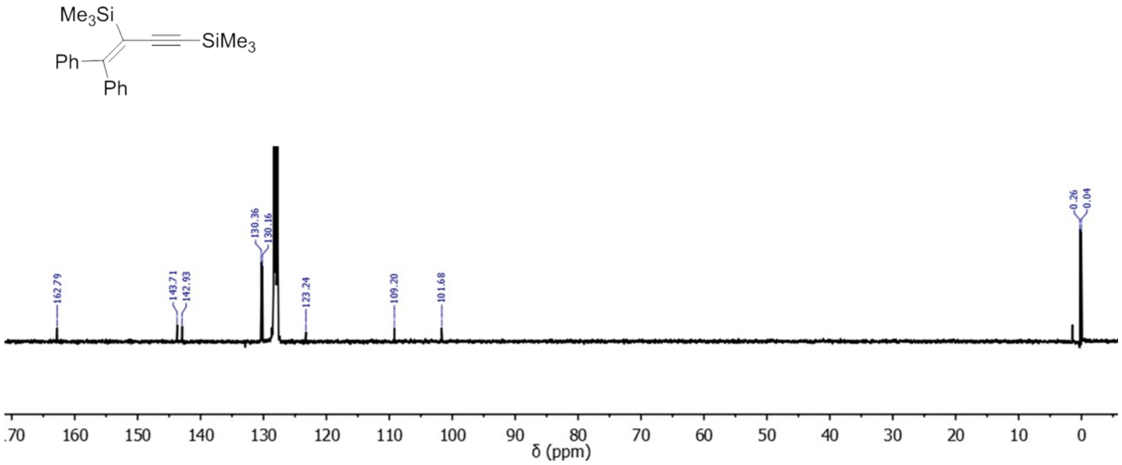

Figure S13: $^{13}C\{^{1}H\}$ NMR spectrum of **3** (25 °C, [D$_6$]benzene, 100.61 MHz).

5.4. ^{1}H and ^{13}C NMR spectra of 4

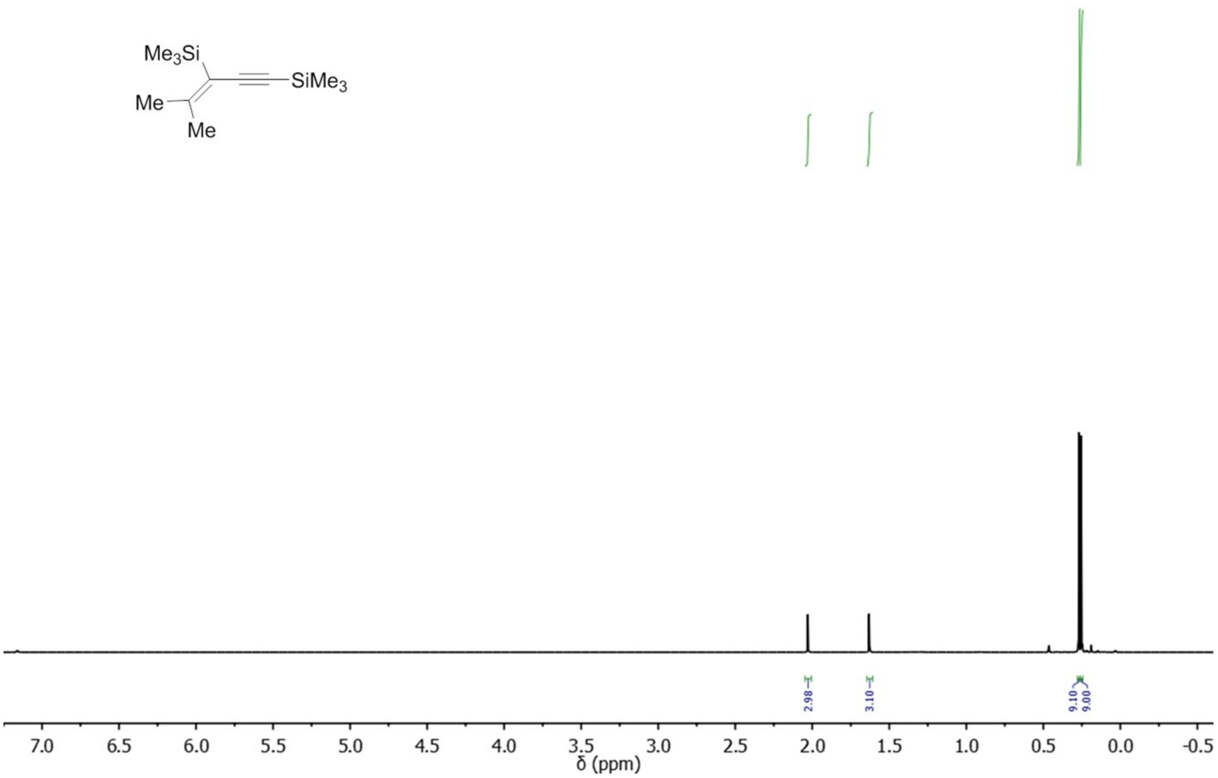

Figure S14: ^{1}H NMR spectrum of **4** (25 °C, [D_6]benzene, 400.13 MHz).

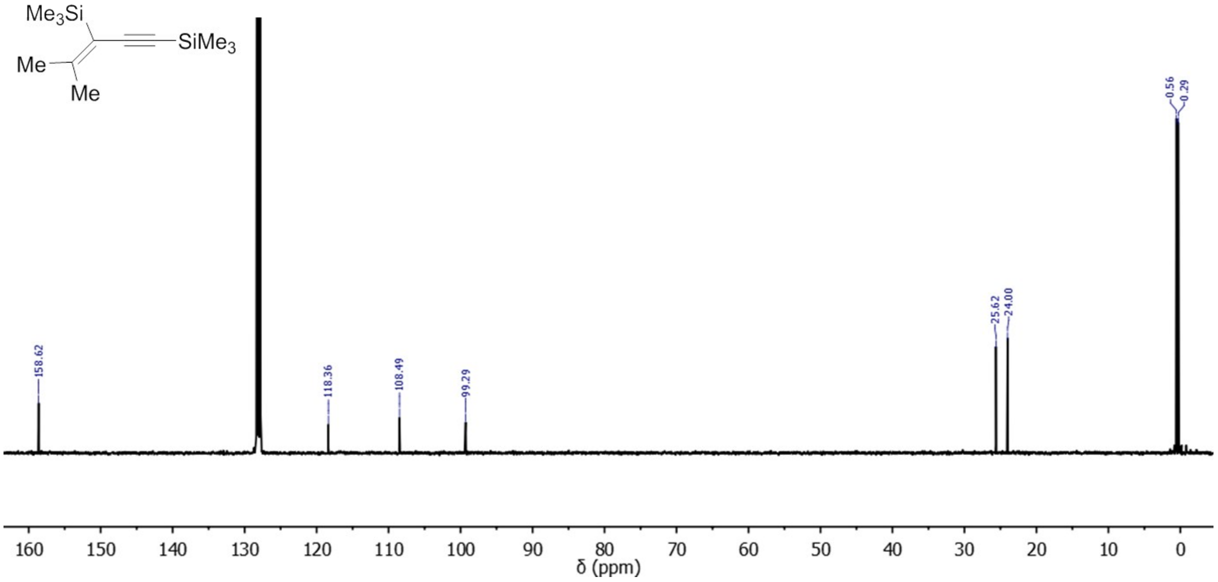

Figure S15: ^{13}C{^{1}H} NMR spectrum of **4** (25 °C, [D_6]benzene, 100.61 MHz).

5.5. ¹H and ¹³C NMR spectra of 5*E* and 5*Z*.

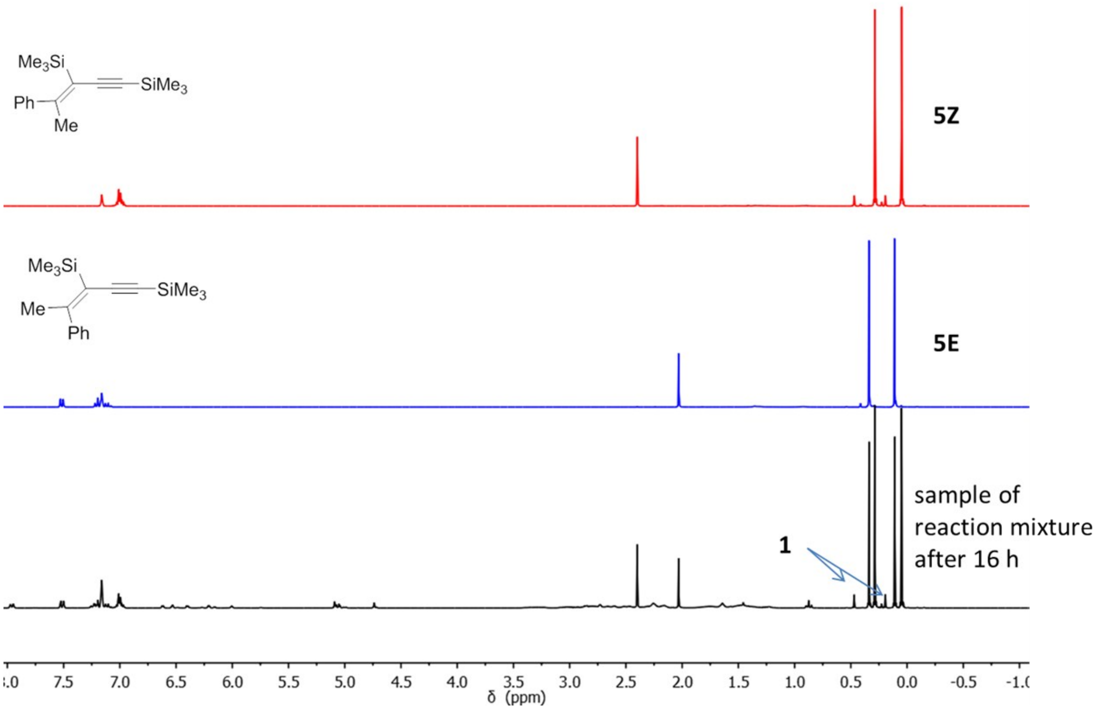

Figure S16: ^{1}H NMR spectrum of **5*E*** and **5*Z*** (25 °C, [D6]benzene, 400.13 MHz).

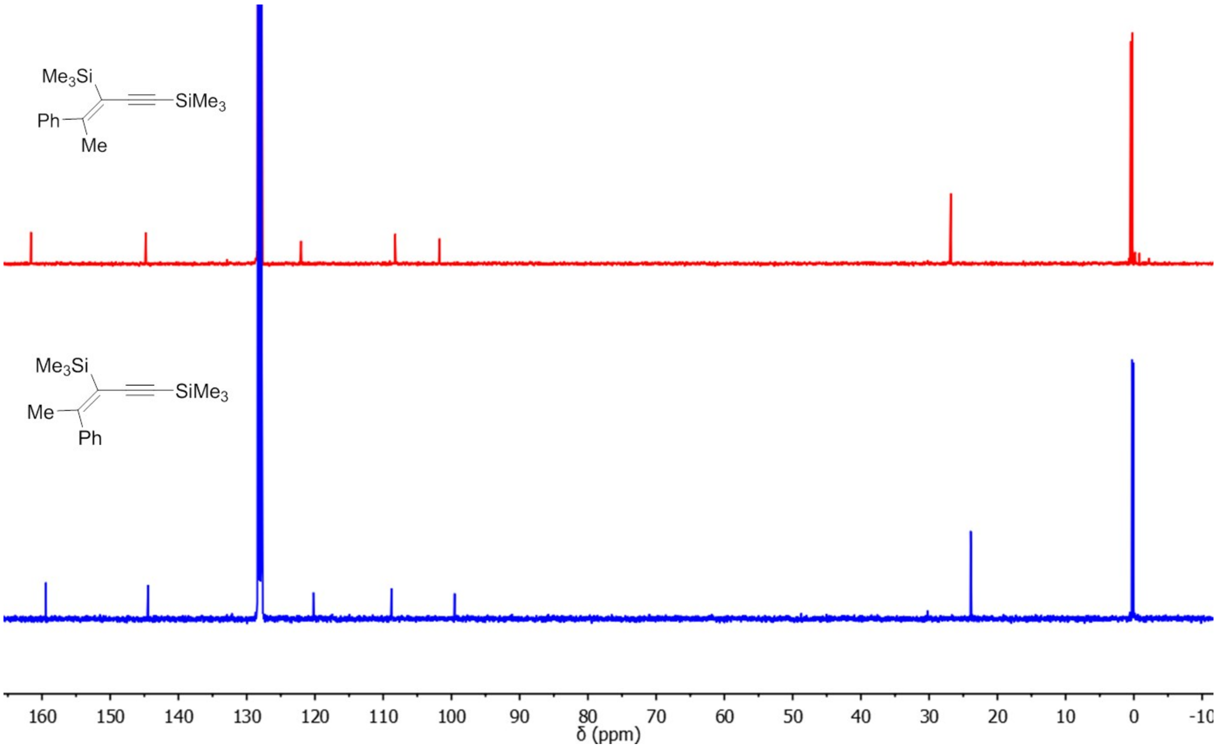

Figure S17: ^{13}C{^{1}H} NMR spectrum of **5*E*** and **5*Z*** (25 °C, [D$_6$]benzene, 100.61 MHz).

5.6. ^{1}H and ^{13}C NMR spectra of 6 isomer mixture

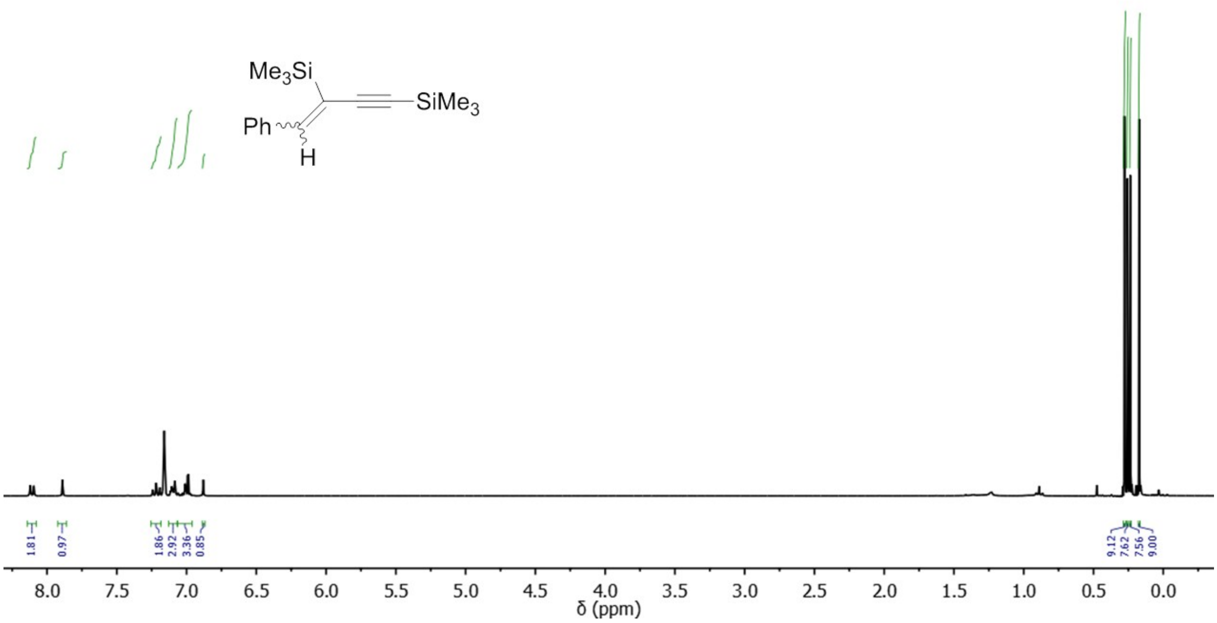

Figure S18: ^{1}H NMR spectrum of *E*/*Z* isomer mixture **6** (25 °C, [D$_6$]benzene, 400.13 MHz).

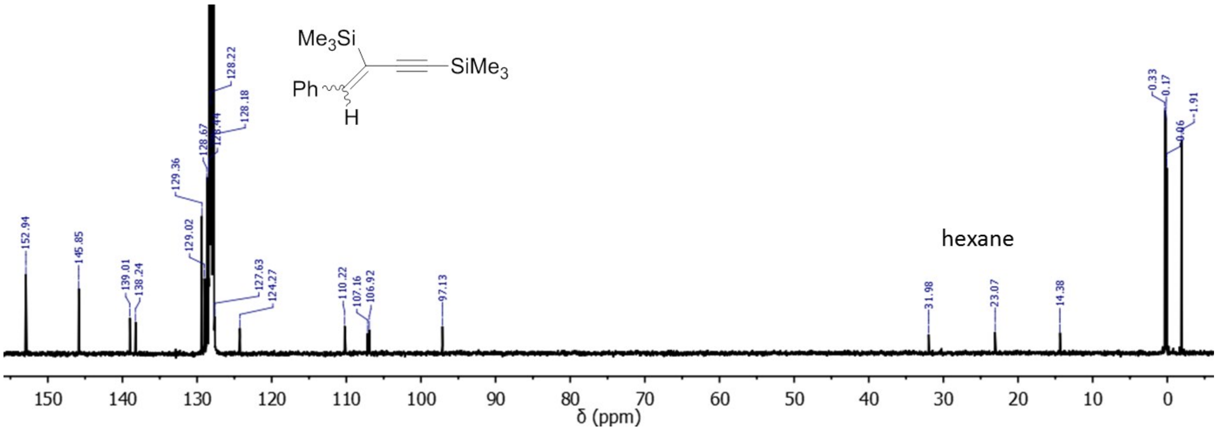

Figure S19: ^{13}C{^{1}H} NMR spectrum of *E*/*Z* isomer mixture **6** (25 °C, [D$_6$]benzene, 100.61 MHz).

5.7. ^{1}H and ^{13}C NMR spectra of 7

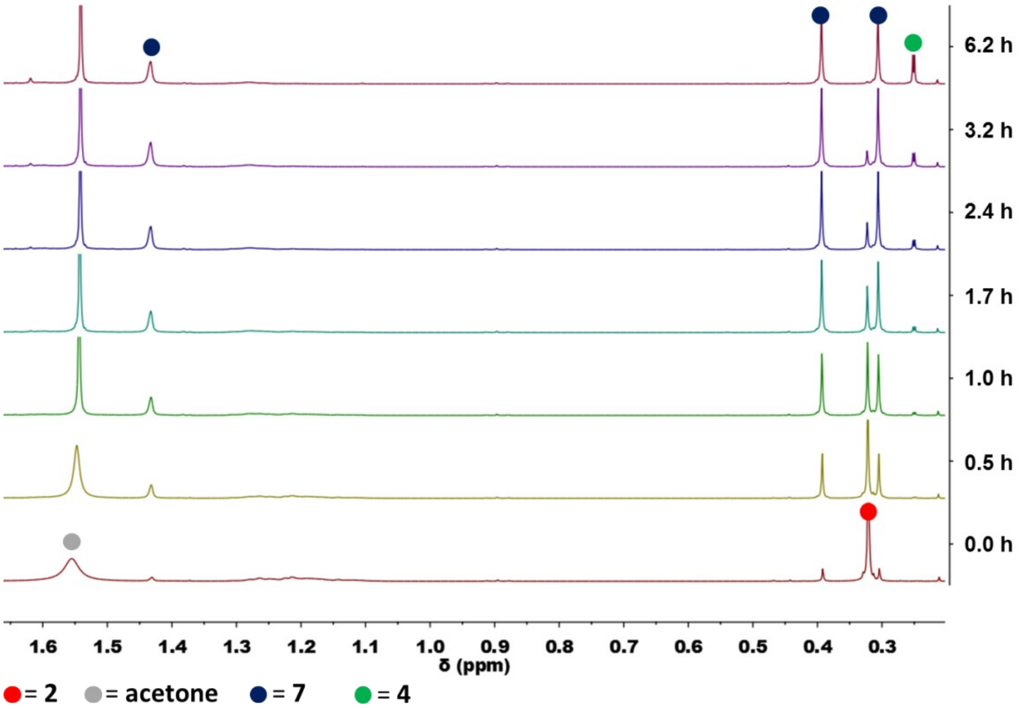

Figure S20: A series of time-dependent low temperature ^{1}H NMR spectra shows the formation of **4** from **2** and acetone via **7** as intermediate species (-10 °C, [D$_8$]toluene, 400.13 MHz, high field).

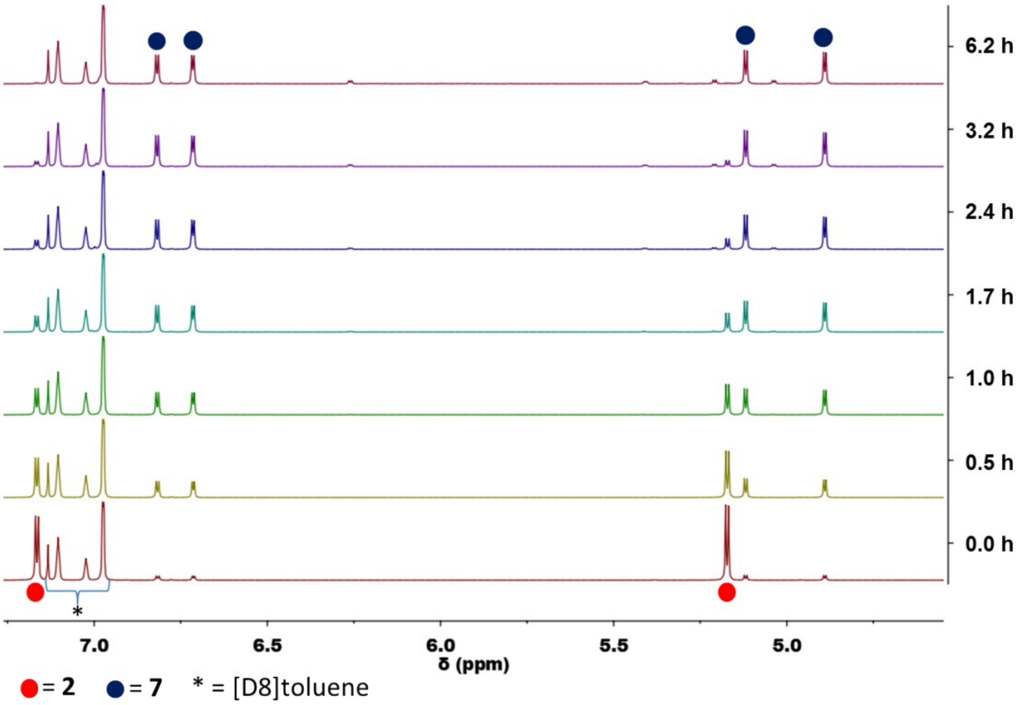

Figure S21: A series of time-dependent low temperature ^{1}H NMR spectra shows the formation of **4** from **2** and acetone via **7** as intermediate species (-10 °C, [D$_8$]toluene, 400.13 MHz, low field).

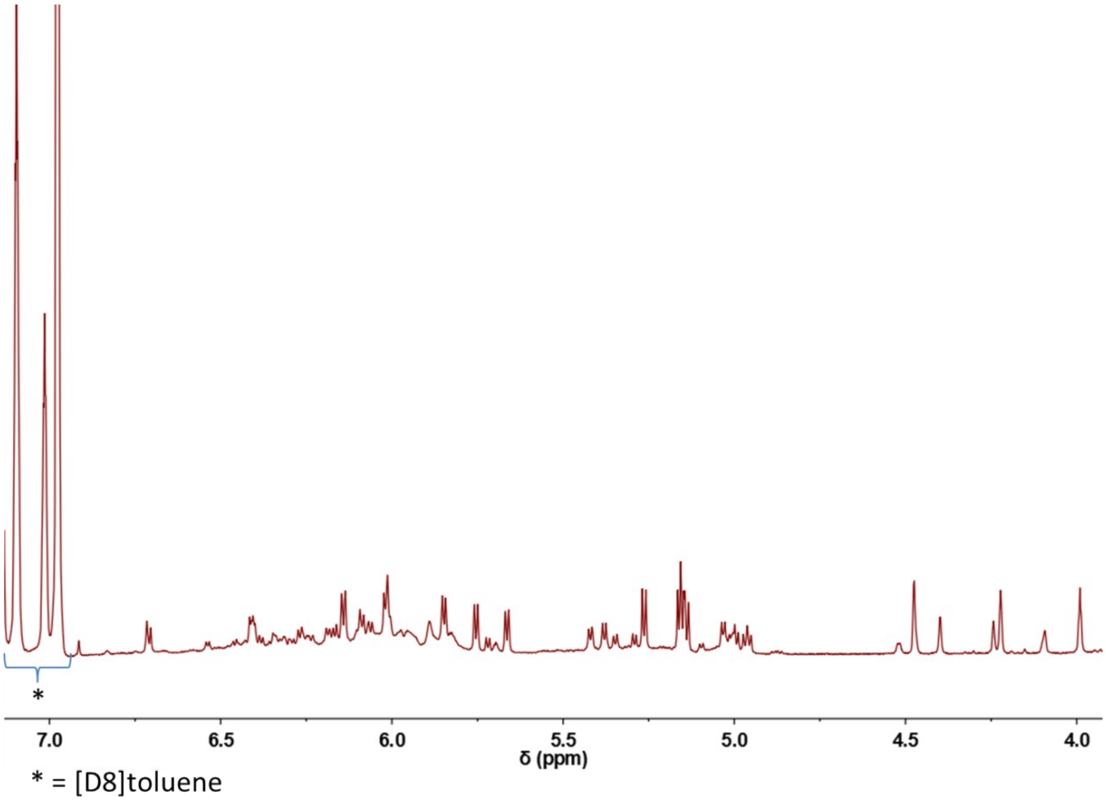

Figure S22: This ^{1}H NMR spectrum was recorded after storing the sample used before (Figure S20 and Figure S21) for 1 day at ambient temperature. It is easy to see from this that the [EBTHI] species under the given conditions is subject to a complex chemical sequence forming a series of unidentified products.

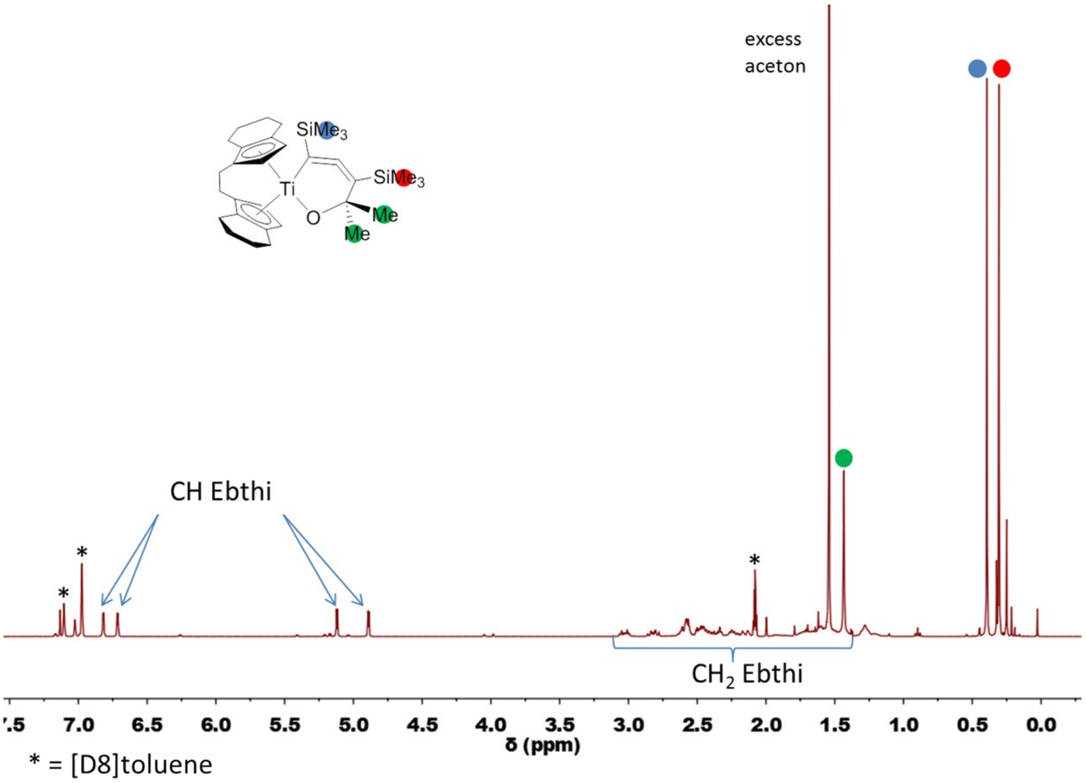

Figure S23: ^{1}H NMR spectrum of **7** (-10 °C, [D_8]toluene, 400.13 MHz). For this spectrum we carried out a low temperature NMR experiment, were the complex **2** was dissolved in [D_8]toluene at ambient temperature; the resulting red solution was then cooled to -50 °C and an excess of acetone was added at this temperature. The sample was positioned in the cooled NMR spectrometer and the reaction was monitored via ^{1}H NMR spectra. This spectrum was recorded after approximately 3 hours reaction time.

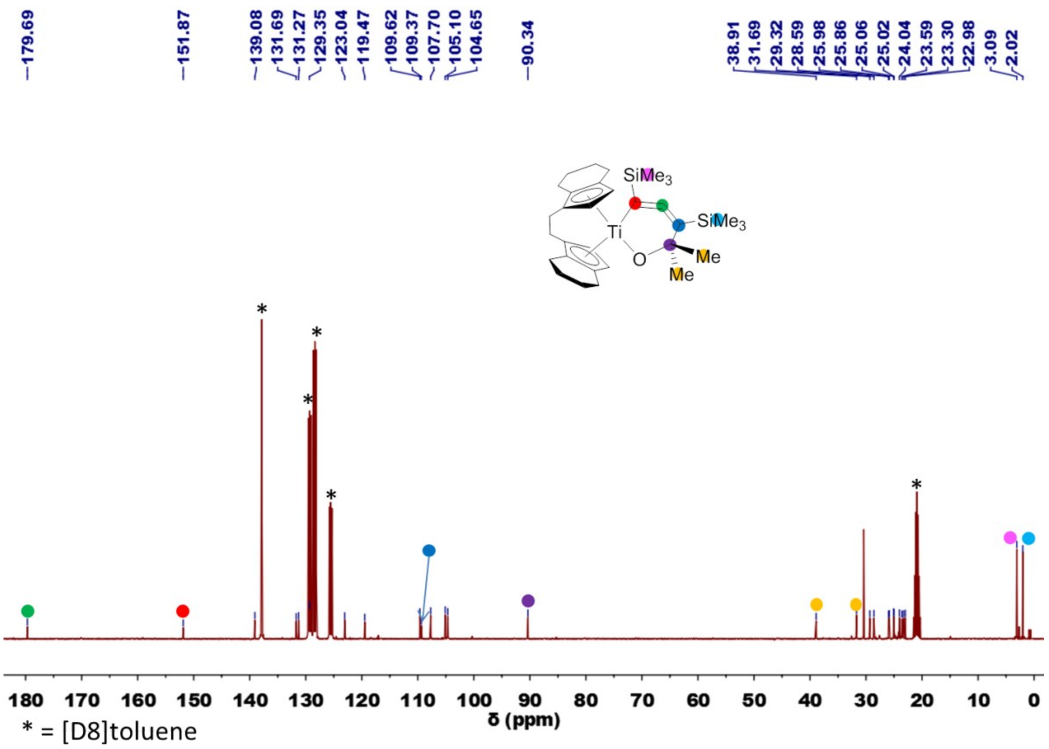

Figure S24: ^{13}C NMR spectrum of **7** (-10 °C, [D$_8$]toluene, 100.61 MHz). Assignment was done with the help of two dimensional ^{1}H,^{13}C HBMC spectroscopy.

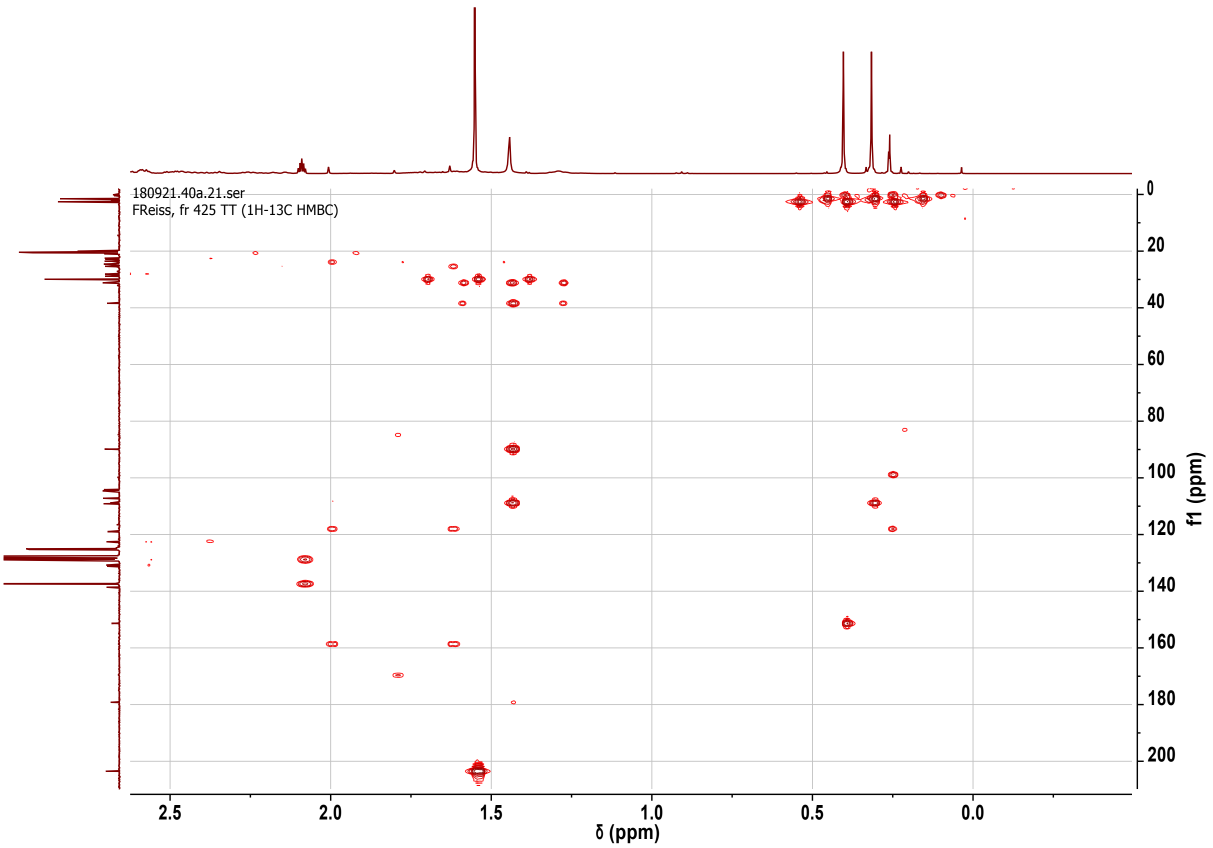

Figure S25: ^{1}H-^{13}C HMBC NMR spectrum of **7** (-10 °C, [D$_8$]toluene, 400.13 MHz, cutout of the high-field region).

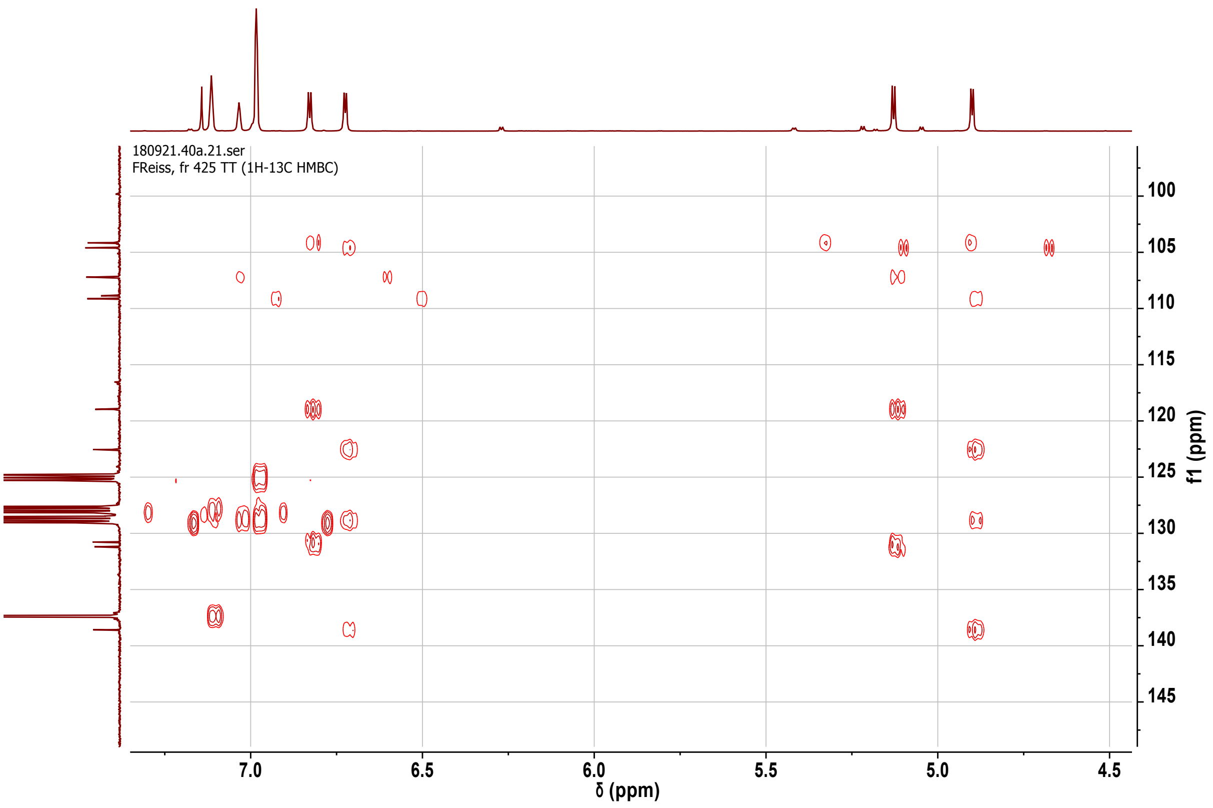

Figure S26: ^{1}H-^{13}C HMBC NMR spectrum of **7** (-10 °C, [D$_8$]toluene, 400.13 MHz, cutout of the high-field region).

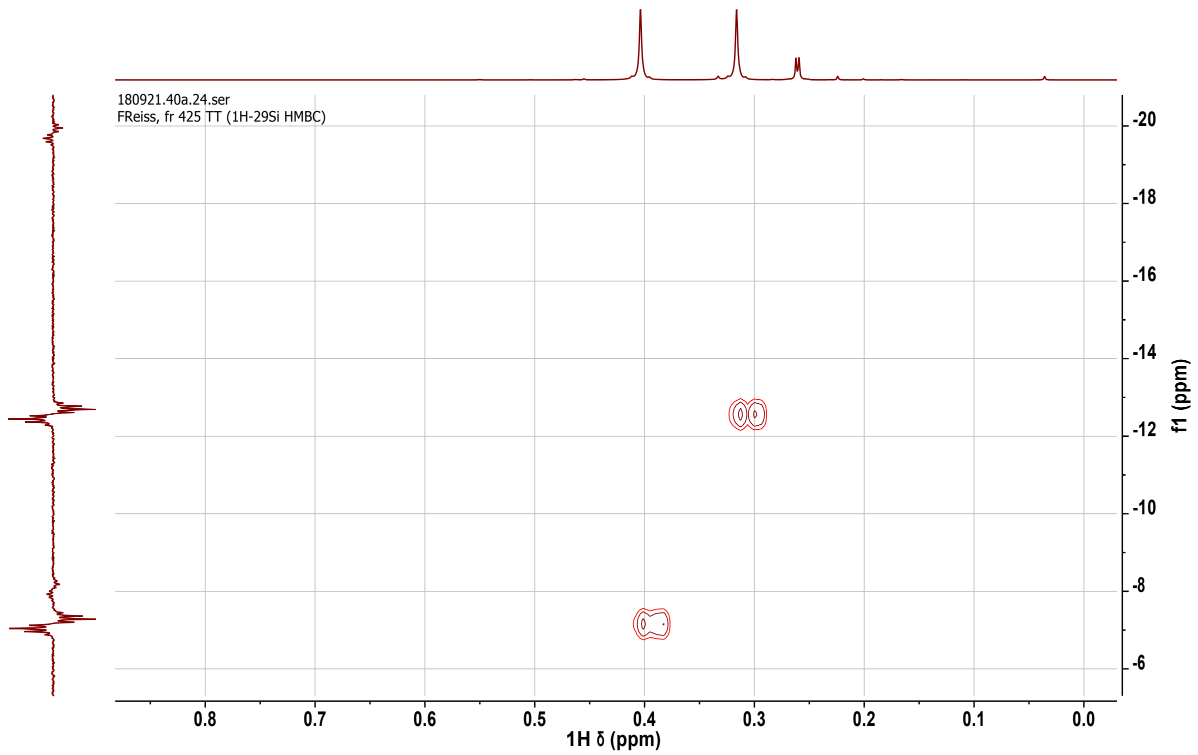

Figure S27: ^{1}H-^{29}Si HMBC NMR spectrum of **7** (-10 °C, [D$_8$]toluene, 400.13 MHz).

5.8. ^{1}H and ^{13}C NMR spectra of 8.

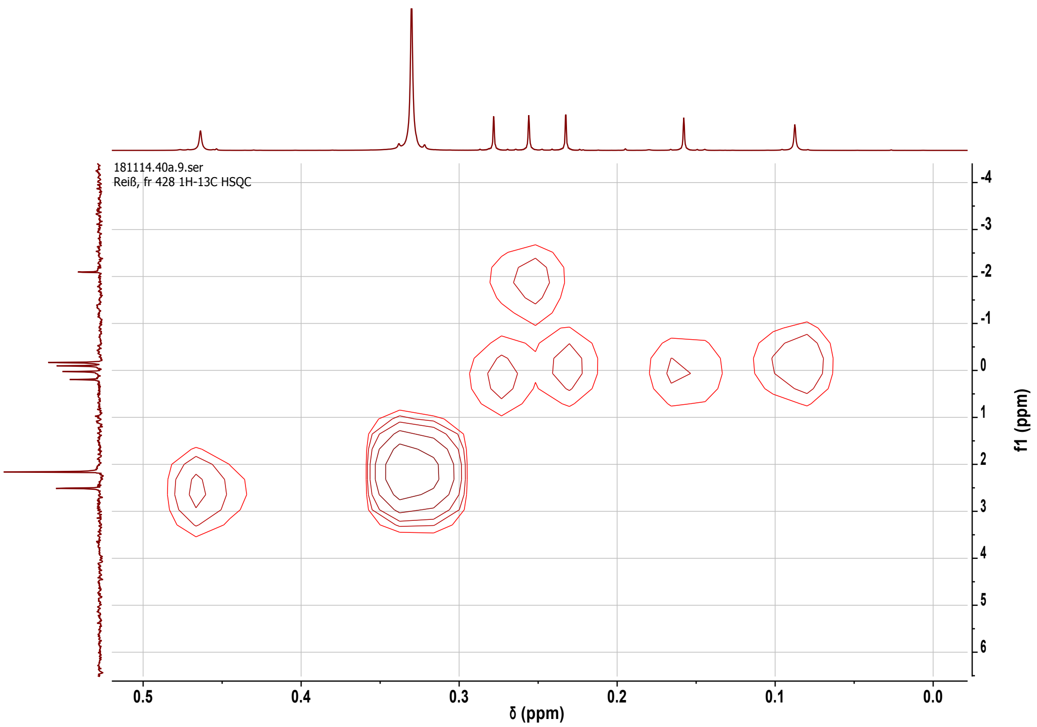

Figure S28: ^{1}H-^{13}C HSQC NMR spectrum of **8** (-10 °C, [D$_8$]toluene, 400.13 MHz, Cutout of the high-field region).

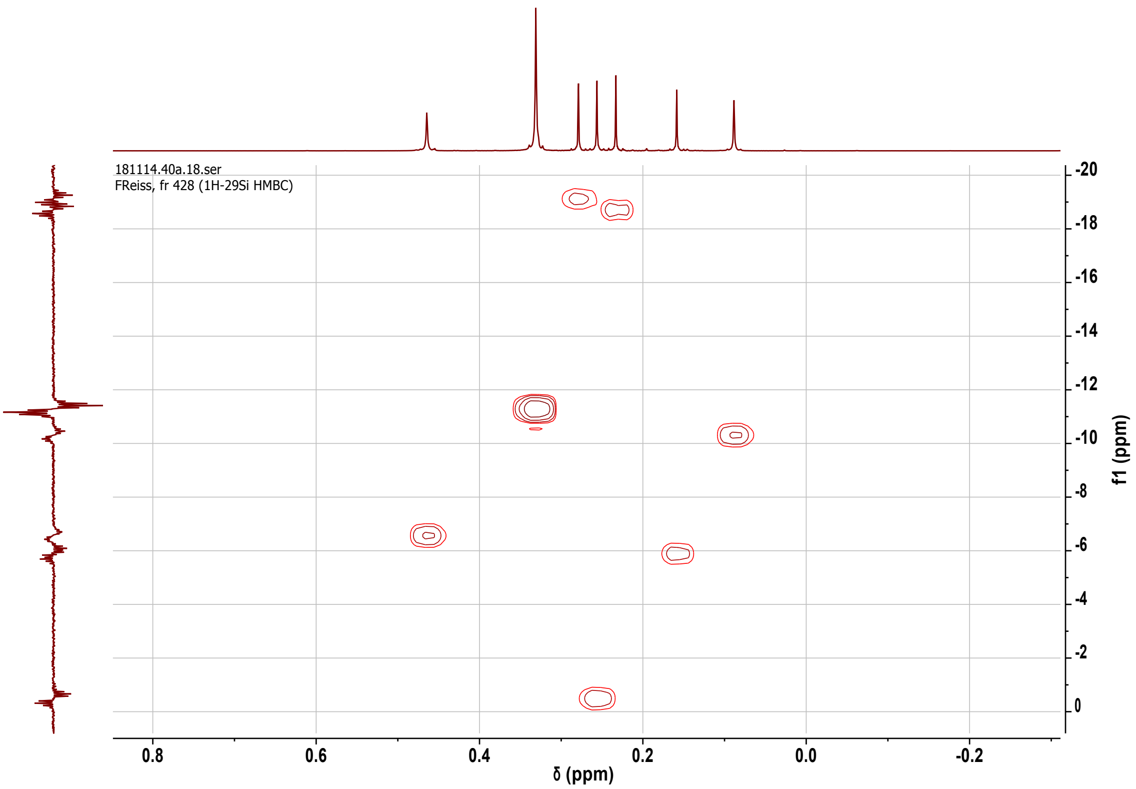

Figure S29: ^{1}H-^{29}Si HMBC NMR spectrum of **8** (-10 °C, [D$_8$]toluene, 400.13 MHz, Cutout of the high-field region).

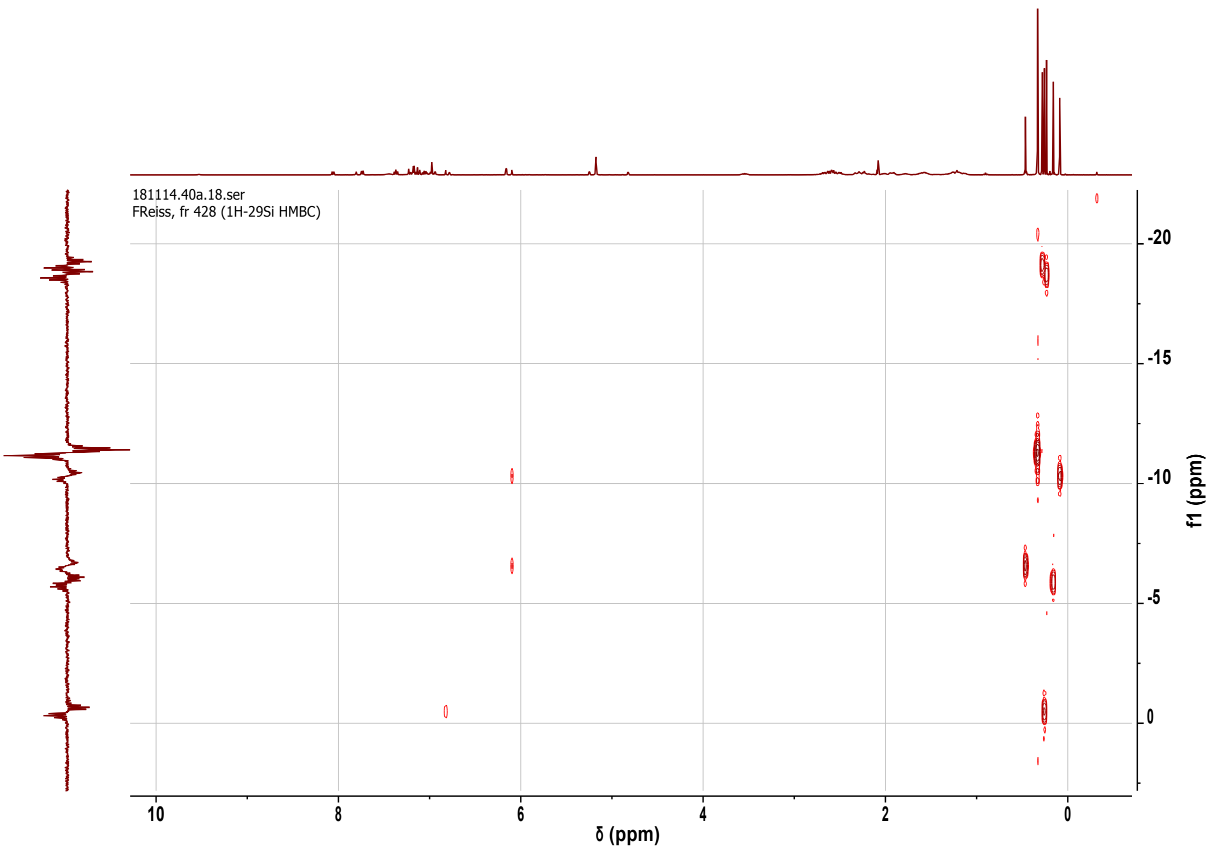

Figure S30: ^{1}H-^{29}Si HMBC NMR spectrum of **8** (-10 °C, [D$_8$]toluene, 400.13 MHz).

6. Details of vibrational spectroscopy

6.1. Assignment of the most important vibrations

In this chapter the experimental IR and Raman spectra (black) with their respective calculated uncorrected vibration spectra (red) are presented. The calculated spectra were taken from the frequency analyses with BP86/LANL2DZ/TZVP level of theory.

Table S7: Assignment of the most important vibrations of compound **2.**

Compound	C1=C2=C3 in-phase vib. calc.	C1=C2=C3 in-phase vib. exp.	C1=C2=C3 out-of-phase vib. calc.	C1=C2=C3 out-of-phase vib. exp.
**2**	1343 cm^{-1}	1344 cm^{-1}	1787 cm^{-1}	1729 cm^{-1}
**7**	[a]	[a]	1840 cm^{-1}	1805 cm^{-1}

[a] No Raman spectrum was collected of this intermediate species.

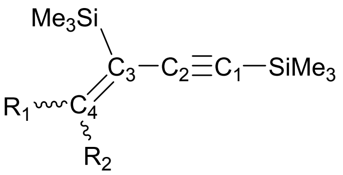

Figure S31: General carbon atom assignment for compounds **3**-**6**.

Table S8: Assignment of the most important vibrations of compounds **1**, **3**, **4**, **5** and **6**.

Compound	C_2=C_3 stretch calc.[a]	C_2=C_3 stretch exp.	C_3=C_4 stretch calc.	C_3=C_4 stretch exp.	C_1≡C_2 stretch calc.	C_1≡C_2 stretch exp.
**1**	1133 cm^{-1}	1140 cm^{-1}	1457 cm^{-1}	1462 cm^{-1}	2104 cm^{-1}[c] 2132 cm^{-1}[d]	2099 cm^{-1}[c] 2121 cm^{-1}[d]
**3**	1092 cm^{-1}	1106 cm^{-1}	1503 cm^{-1}	1534 cm^{-1}	2125 cm^{-1}	2120 cm^{-1}
**4**	1134 cm^{-1}	1128 cm^{-1}	1573 cm^{-1}	1586 cm^{-1}	2136 cm^{-1}	2117 cm^{-1}
**5E**	1121 cm^{-1}	1125 cm^{-1}	1539 cm^{-1}	1552 cm^{-1}	2133 cm^{-1}	2115 cm^{-1}
**5Z**	1135 cm^{-1}	1137 cm^{-1}	1546 cm^{-1}	1559 cm^{-1}	2134 cm^{-1}	2112 cm^{-1}
**6E**	1054 cm^{-1}	1062 cm^{-1}	1545 cm^{-1}	[b]	2122 cm^{-1}	[b]
**6Z**	1110 cm^{-1}	1111 cm^{-1}	1540 cm^{-1}	[b]	2136 cm^{-1}	[b]

[a] This vibration mixes strongly with CH vibrations of the substituents at carbon atom C4. [b] Vibrations cannot be unambiguously assigned. [c] C1≡C2 and C1'≡C2' in-phase vibration [d] C1≡C2 and C1'≡C2' out-of-phase vibration

6.2. Experimental and calculated vibrational spectra

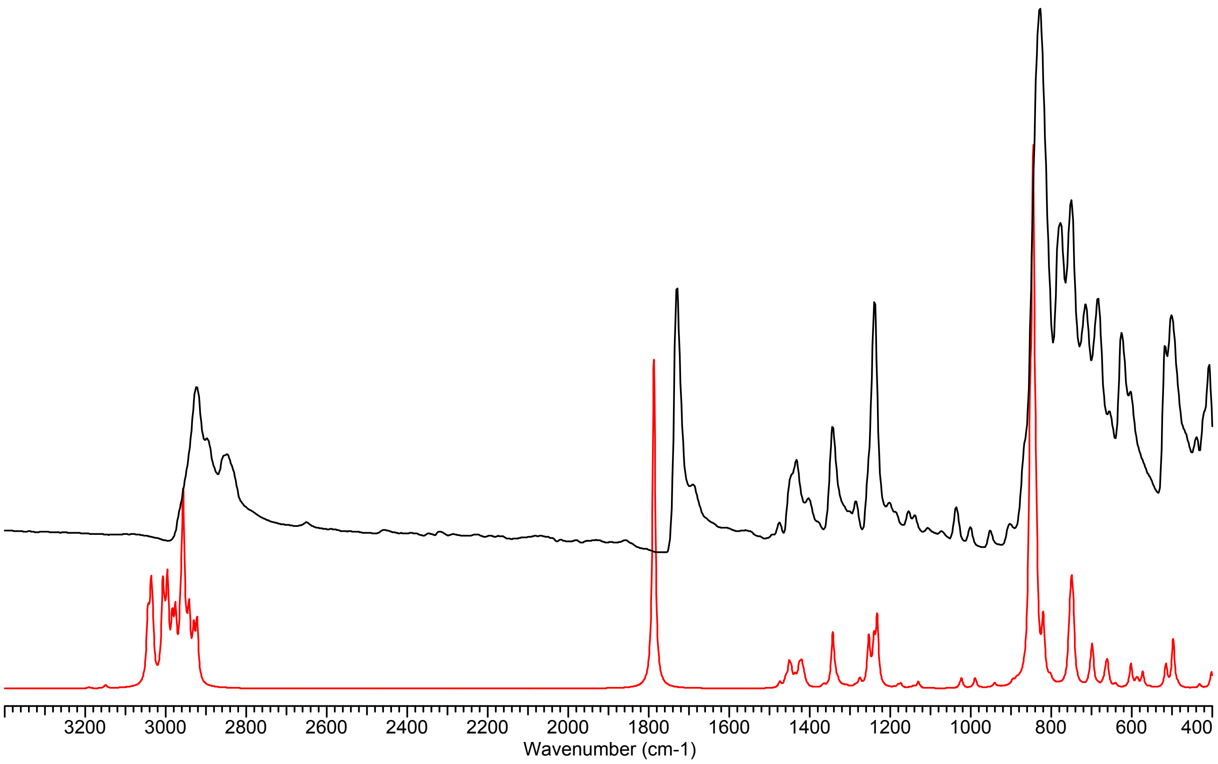

Figure S32: Experimental (black) and calculated (red) IR spectra of **2**

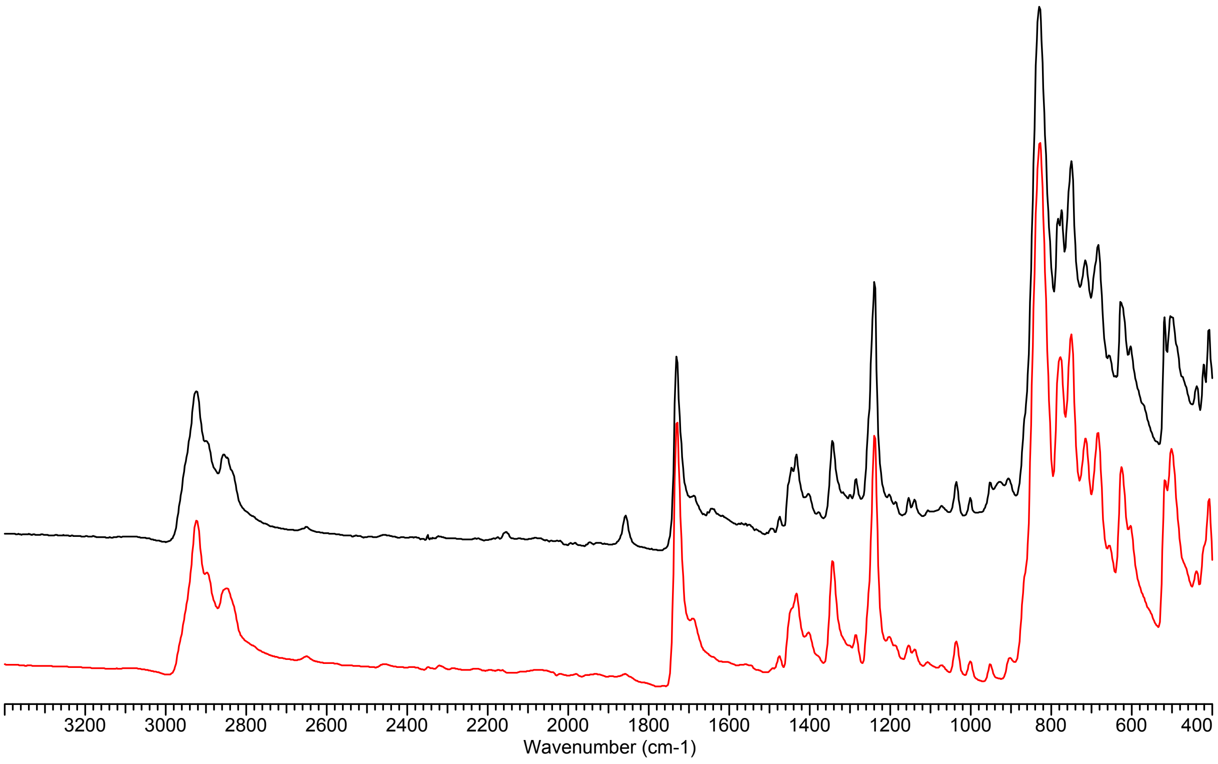

Figure S33: IR spectra of **2** immediately measured (red) and after 2 minutes exposure to air (black). The black spectrum features a characteristic vibration at 2157 cm^{-1} for the bis-(trimethylsilyl)-propyne.

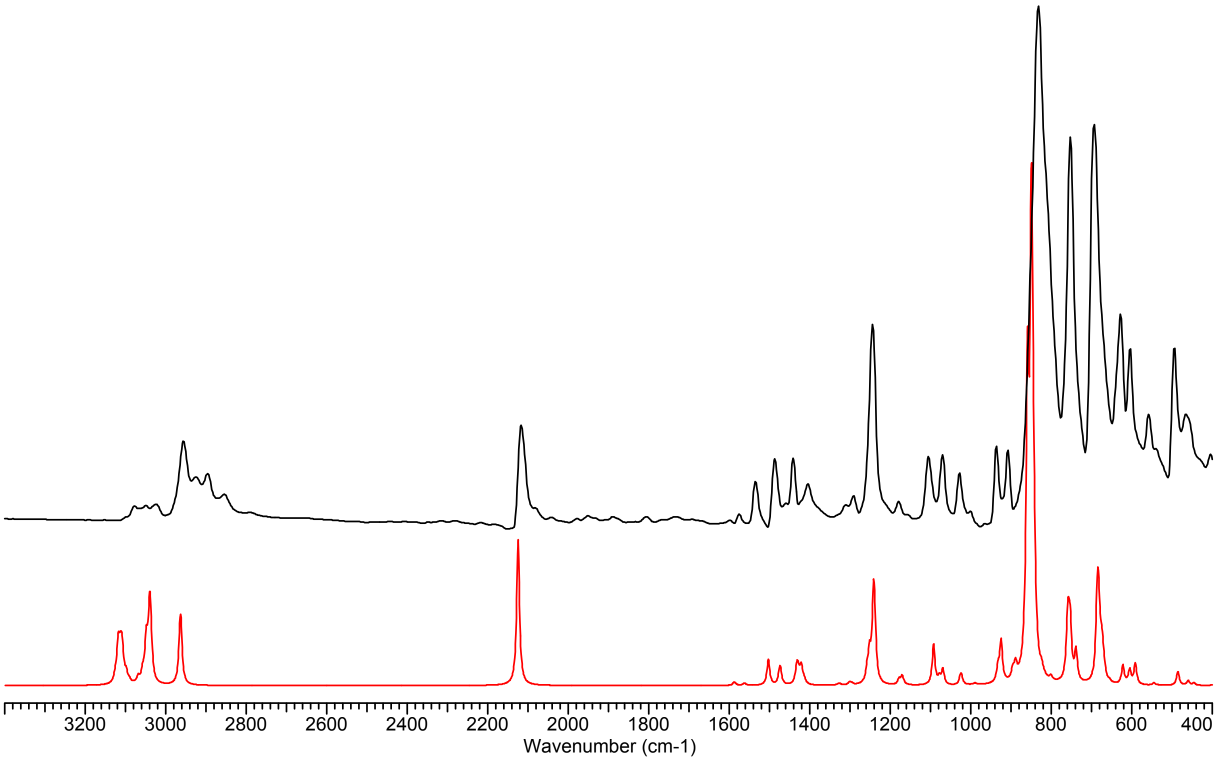

Figure S34: Experimental (black) and calculated (red) IR spectra of **3**.

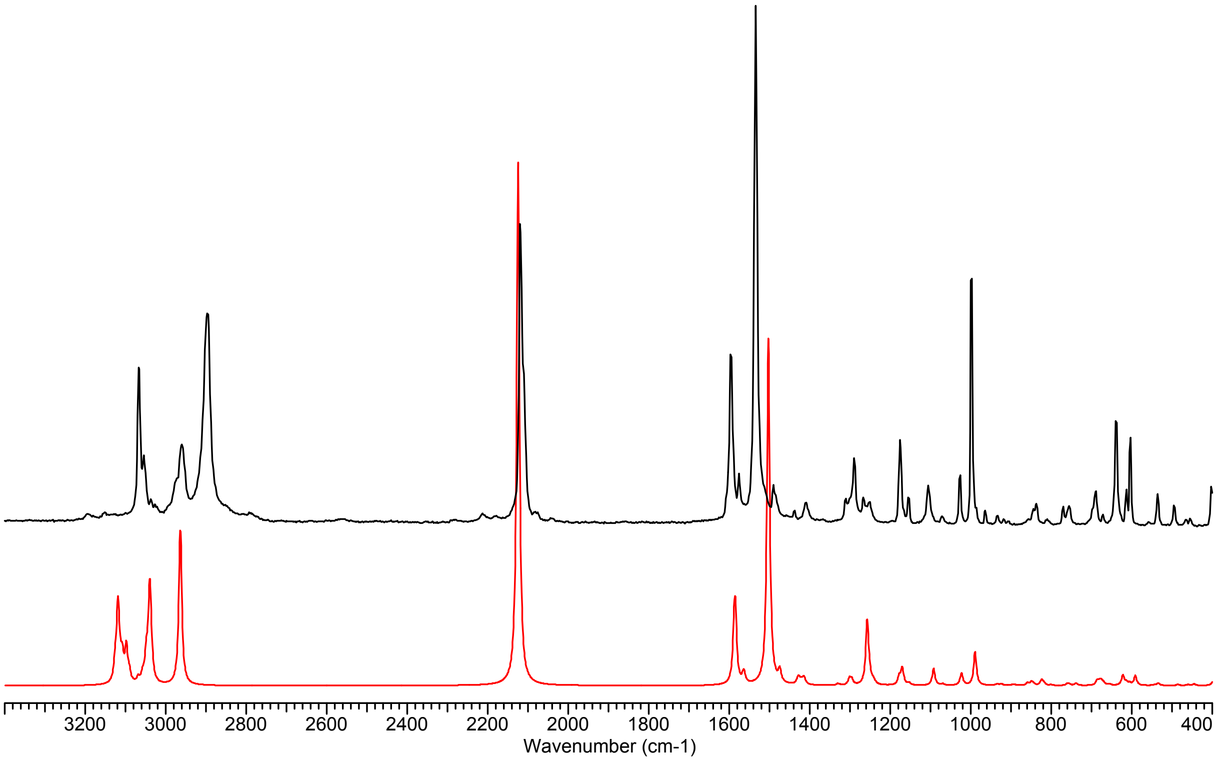

Figure S35: Experimental (black) and calculated (red) Raman spectra of **3**.

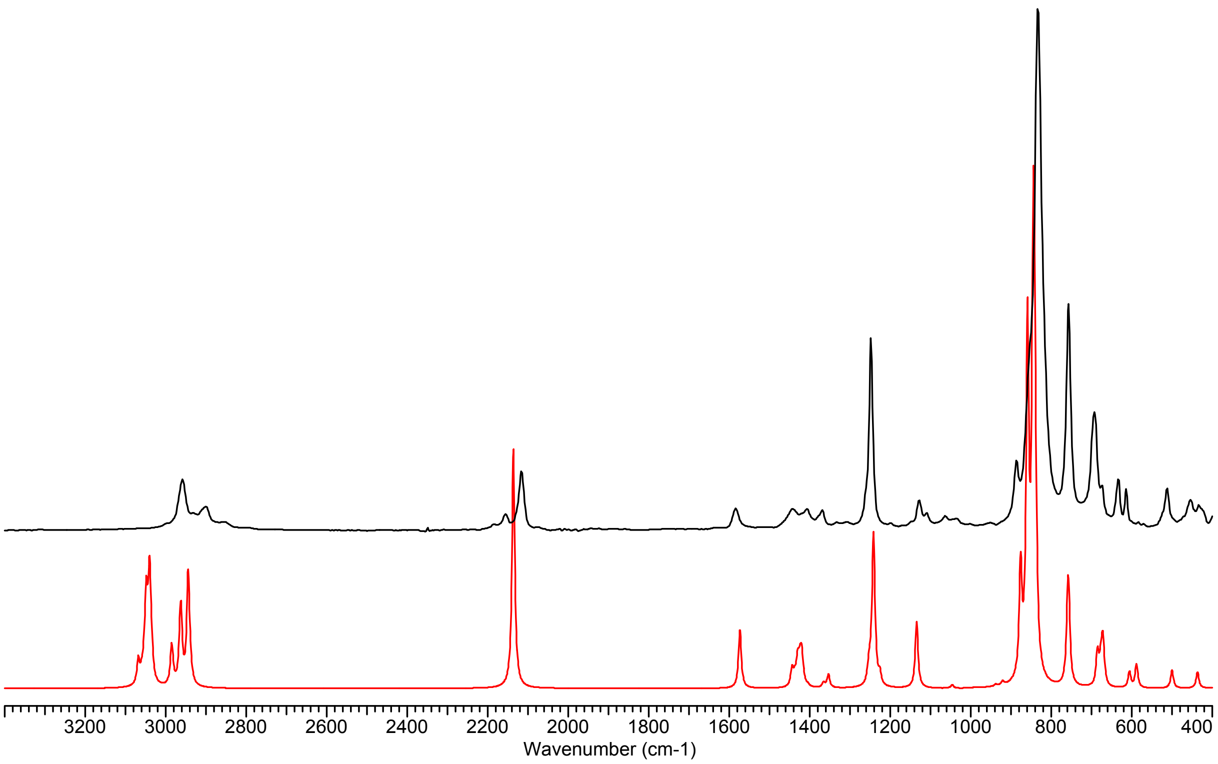

Figure S36: Experimental (black) and calculated (red) IR spectra of **4**.

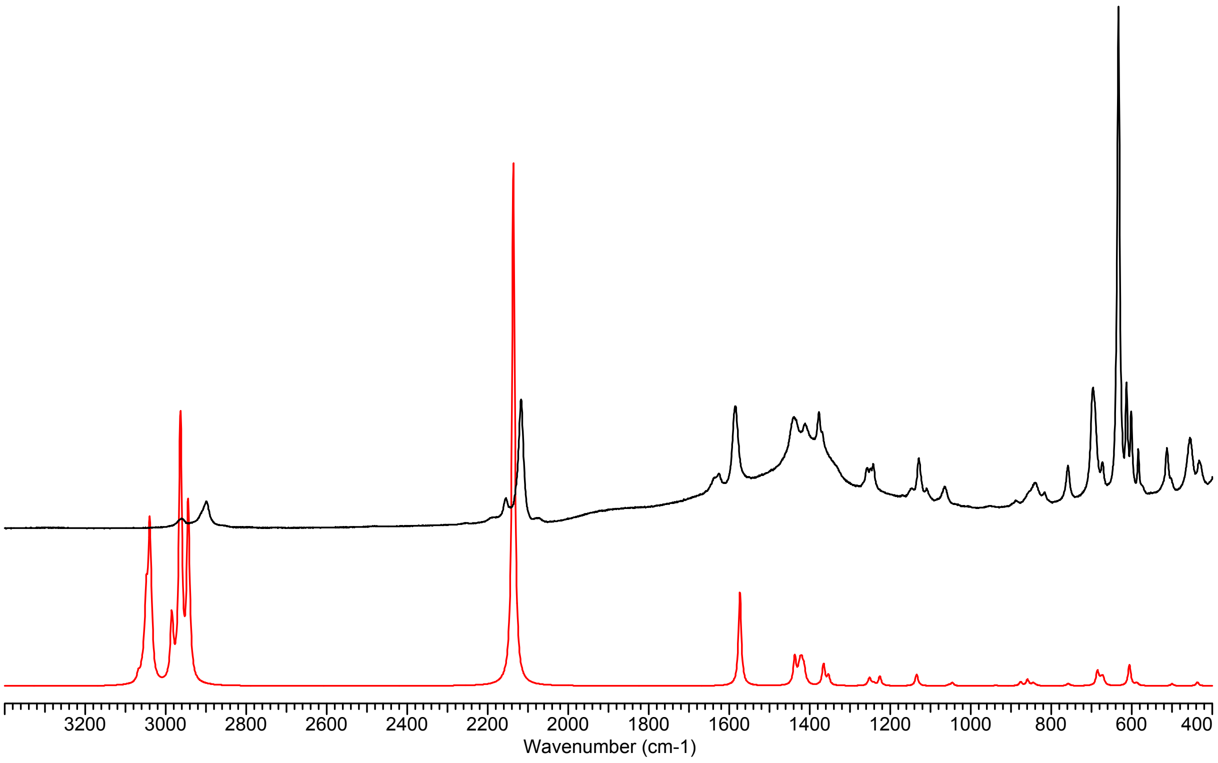

Figure S37: Experimental (black) and calculated (red) Raman spectra of **4**.

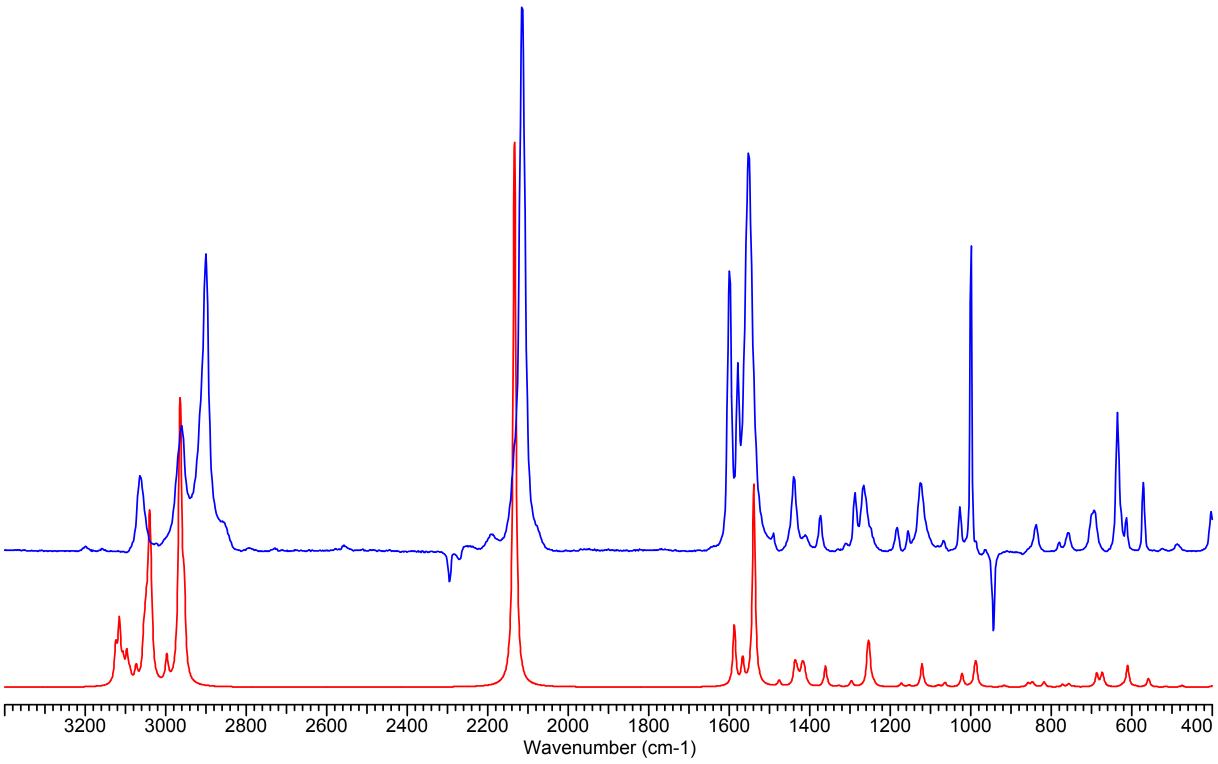

Figure S38: Calculated (red) and experimental (blue) Raman spectra of the *E*-isomer of **5**. The experimental spectrum was in this case baseline corrected and the vibrations of remained benzene are shown with negative values.

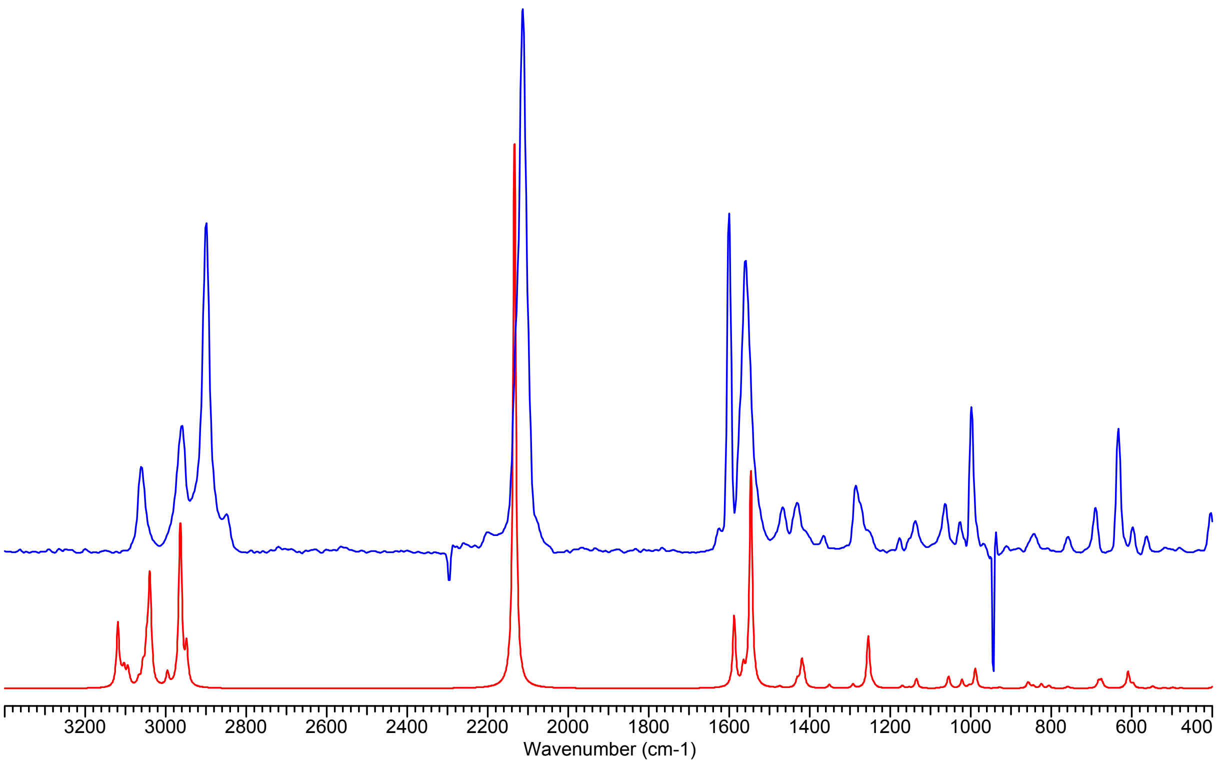

Figure S39: Calculated (red) and experimental (blue) Raman spectra of the *Z*-isomer of **5**. The experimental (blue) spectrum was in this case baseline corrected and the vibrations of remained benzene are shown with negative values.

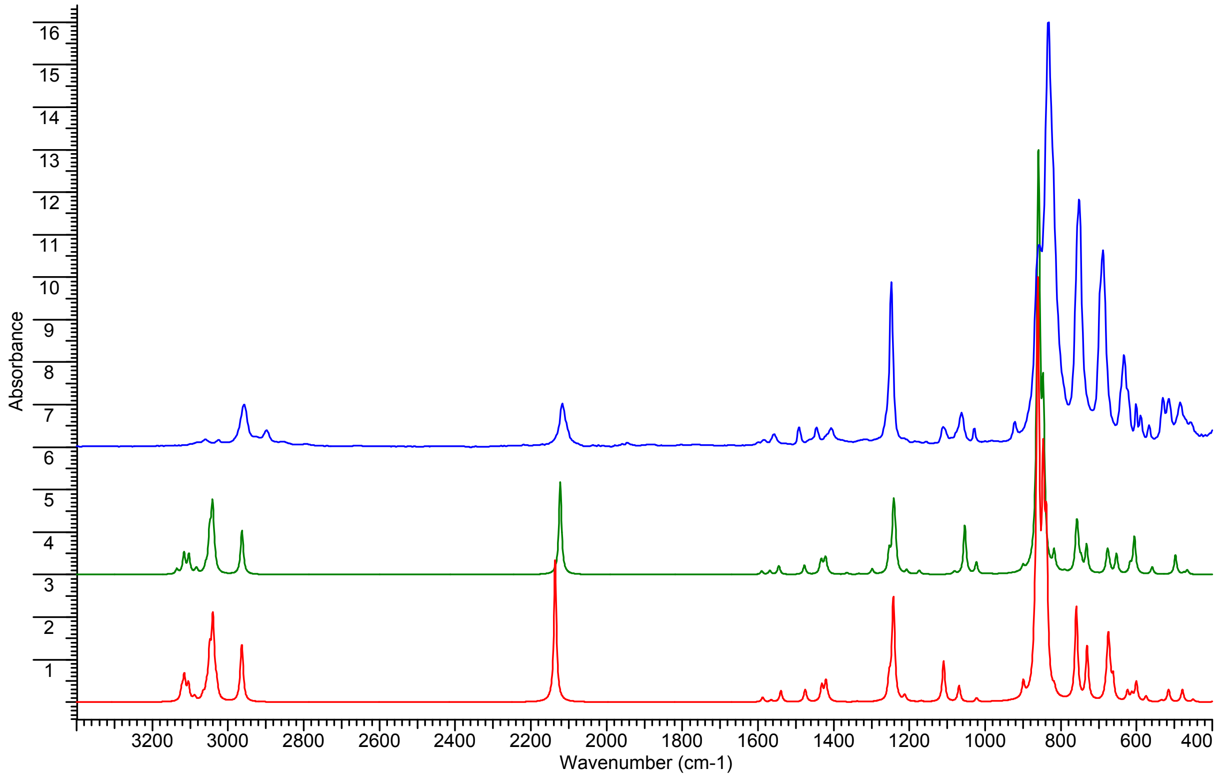

Figure S40: Experimental IR spectrum of isomer mixture of **6** (blue), calculated spectra for *E*-isomer (green) and *Z*-isomer (red).

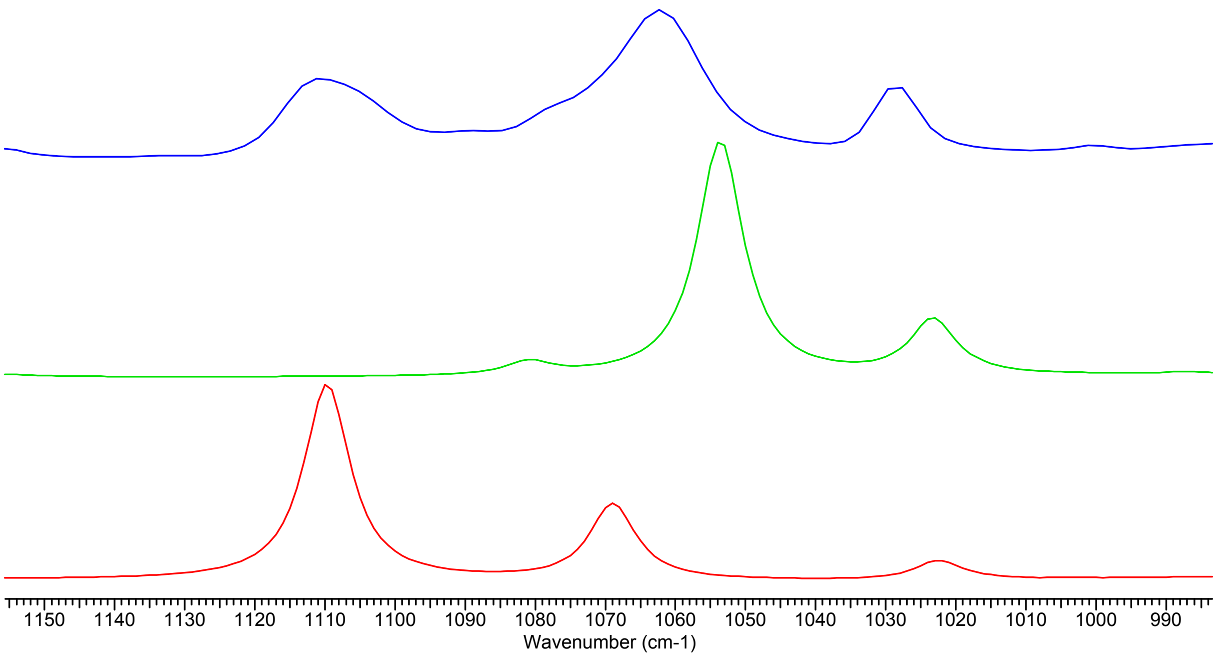

Figure S41: Representation of a selection of the IR spectra of **6** with the most noticeable differences of the *E*/*Z*-isomers which can be assigned to the CH in plane vibrations of the phenyl substituents which are mixed with CC stretching vibrations. Red line represents the calculated spectrum of the *Z* isomer, green for the *E* isomer and the blue spectrum

represents the experimental spectrum, which clearly shows the resulting product as a mixture of *E* and *Z* isomer as confirmed by NMR spectroscopy.

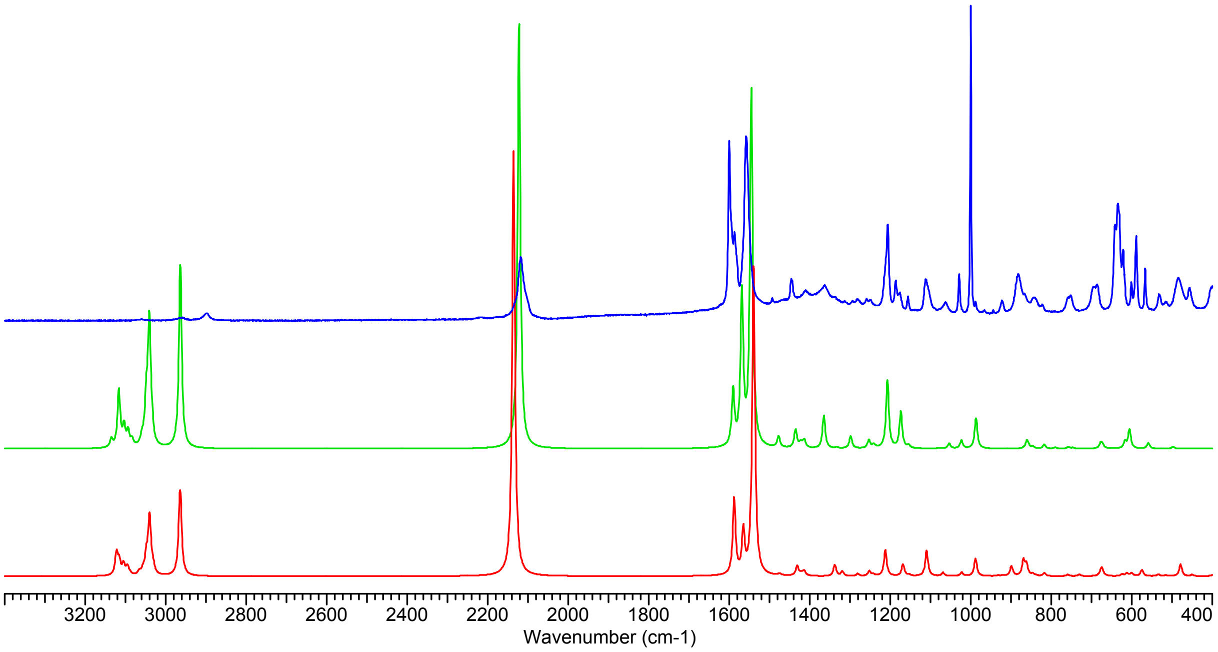

Figure S42: Experimental Raman spectra of isomer mixture of **6** (blue), calculated spectra for *E*-isomer (green) and *Z*-isomer (red).

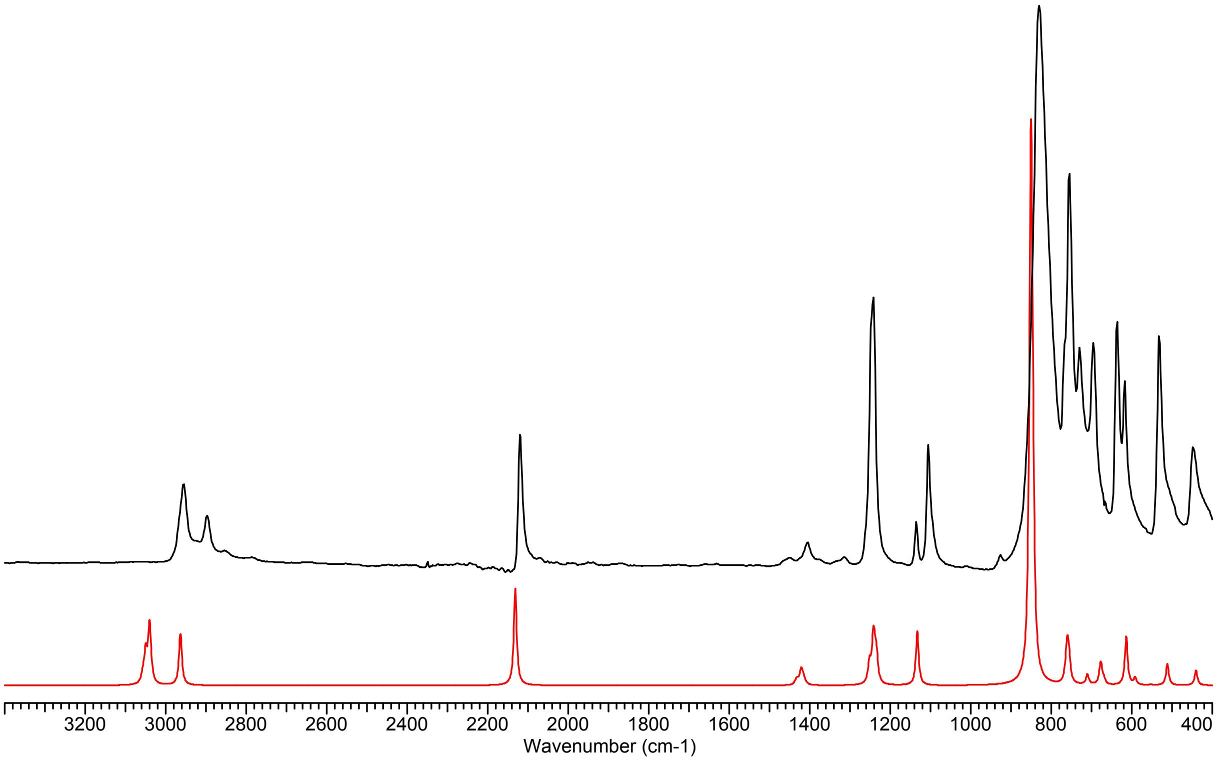

Figure S43: Experimental (black) and calculated (red) IR spectra of **1**.

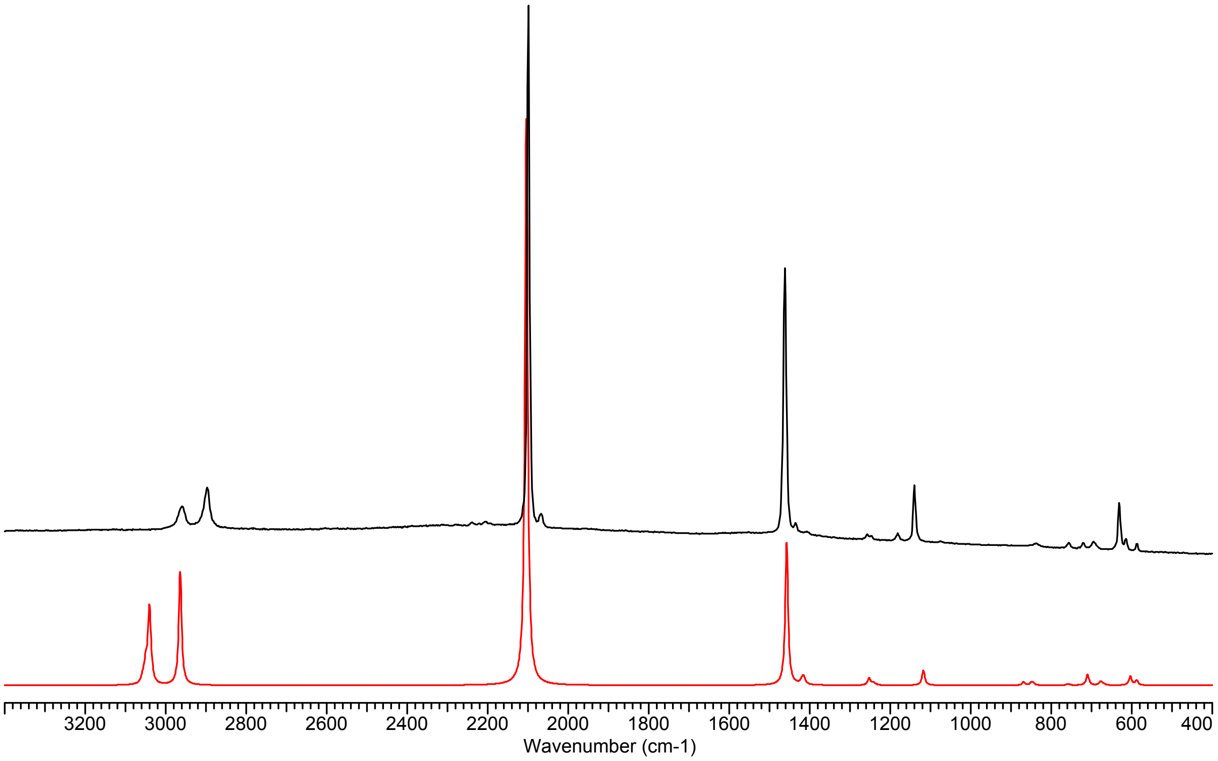

Figure S44: Experimental (black) and calculated (red) Raman spectra of **1**.

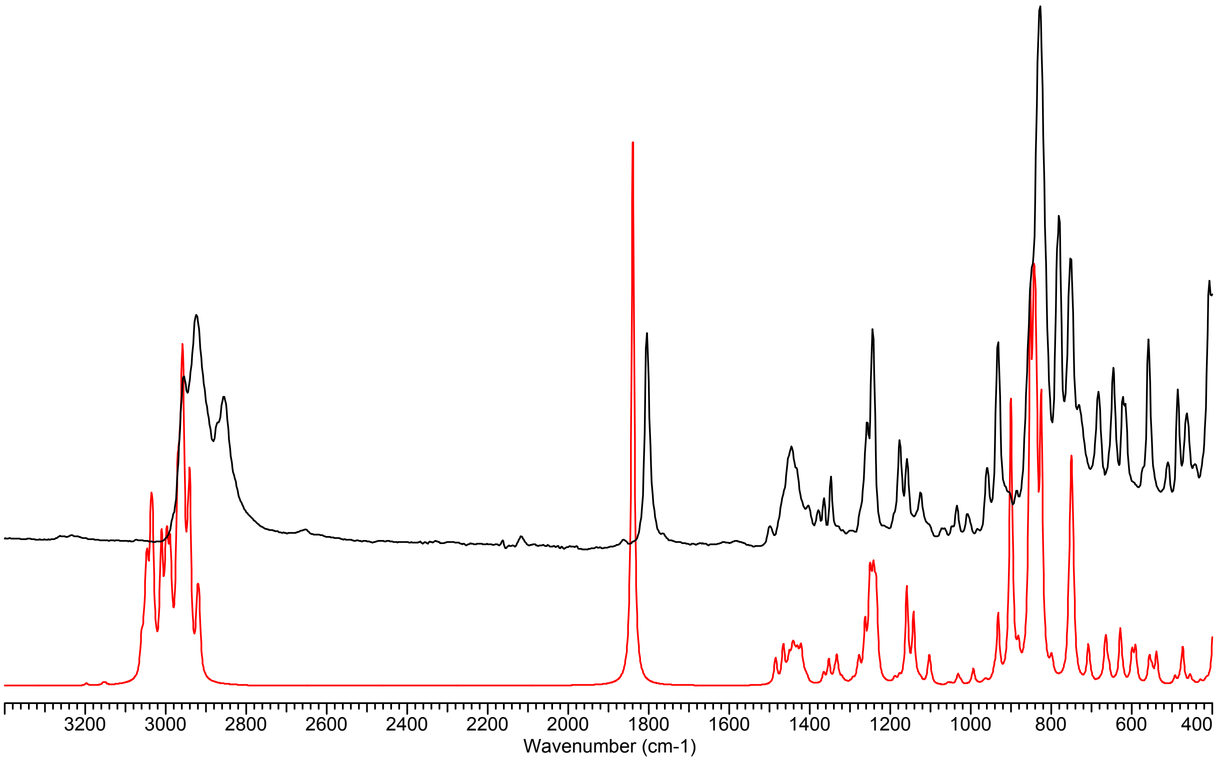

Figure S45: Experimental (black) and calculated (red) IR spectra of **7**.

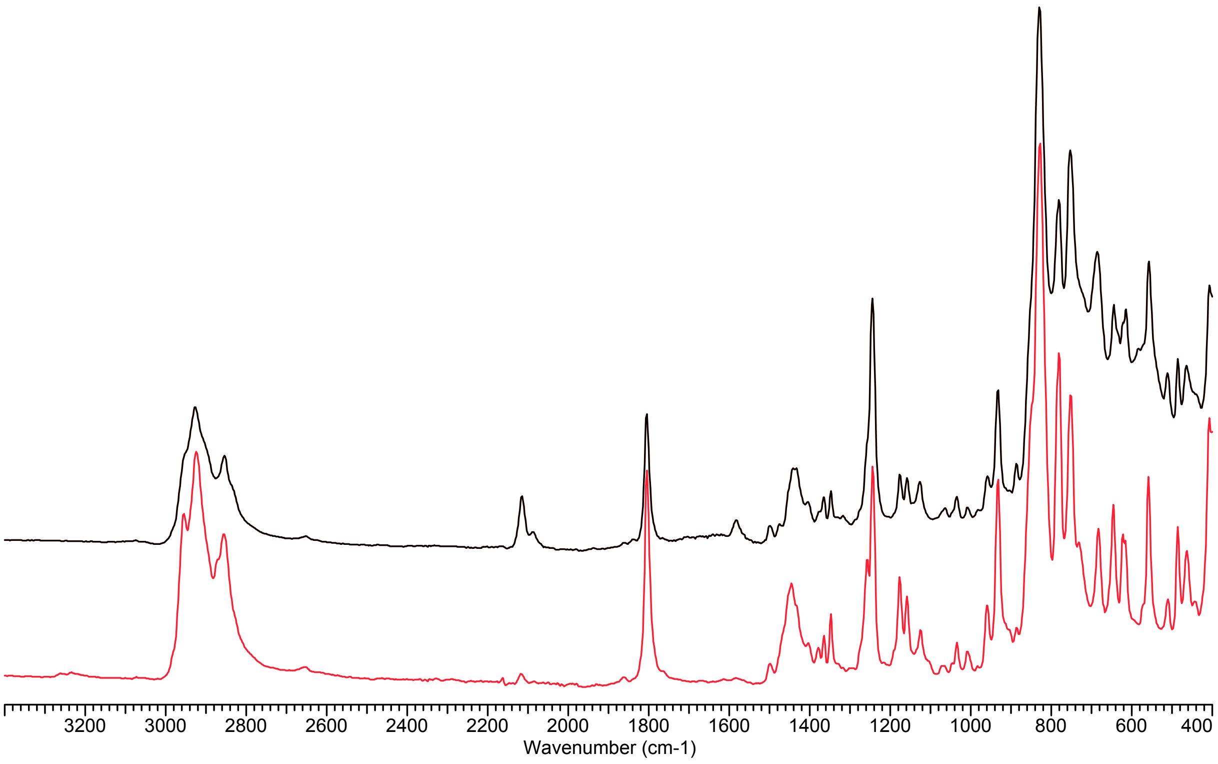

Figure S46: IR spectra of the intermediate species **7** immediately measured (red) and after 3 minutes exposure to air and ambient temperature (black). The black spectrum features a new characteristic vibration at 2115 cm^{-1} which might be assigned to the C1≡C2 stretch vibration of **4** which is formed due to the ambient temperature measurement.

7. Computational Details

All calculations were carried out with the Gaussian 09 package of molecular orbital programs.[6] In a first step we carried out an optimisation test with real-size molecule **2**, in this study we compared the Methods BP86,[12] B3LYP[12,13] and PBE1PBE[14] as well as the basis sets def2-TZVP,[15] {TZVP(C, H, Si);[16] LANL2DZ(Ti)[17]} and aug-cc-pvdz.[18] The main result is that pure density functional (DF) BP86 in combination with the LANL2DZ basis set and corresponding effective core potential (ECP) at Ti and the TZVP basis set on all other atoms (notation BP86/LANL2DZ/TZVP) is clearly the best combination for the metallacyclic systems, both in terms of performance and HF energy (see Table S9). Therefore, if not further mentioned the energies and discussed results were performed with this procedure. Vibrational frequencies were also computed, to include zero-point vibrational energies in thermodynamic parameters and to characterise all structures as minima on the potential energy surface. In addition, we used these results to assign the experimental IR and RAMAN spectra and to superimpose the experimental and calculated vibration spectra (see above). NBO analyses were performed using NBO 6.0.[19] QT-AIM and ELF calculations were performed using MultiWfn 3.5.[20]

7.1. Comparison of Different Methods and Basis sets.

Table S9: Comparison of Different Methods and Basissets.

Method	BP86	B3LYP	PBE1PBE
Basis set	def2TZVP	def2TZVP	def2TZVP
Complex **2**	HF= -2559.5008685<br>ZPE= 383.50824 (Kcal/Mol)<br>NImag=0<br>Htot= -2558.850669<br>Gtot= -2558.956402	HF= -2559.4045565<br>ZPE= 394.72178 (Kcal/Mol)<br>NImag=0<br>Htot= -2558.737339<br>Gtot= -2558.841994	HF= -2557.5462952<br>ZPE= 396.57926 (Kcal/Mol)<br>NImag=0<br>Htot= -2556.876393<br>Gtot= -2556.979888
CPU Time	2 d15 h 20 m 34.2 s	8 d 4 h 43 m 11.1 s	8 d 3 h 45 m 55.4 s
Method	BP86	B3LYP	PBE1PBE
Basis set	TZVP (C,H,Si)<br>LANL2DZ (Ti)	TZVP (C,H,Si)<br>LANL2DZ (Ti)	TZVP (C,H,Si)<br>LANL2DZ (Ti)
Complex **2**	HF= -1767.9906562<br>ZPE= 383.54489 (Kcal/Mol)<br>NImag=0<br>Htot= -1767.340175<br>Gtot= -1767.446884	HF= -1767.9472903<br>ZPE= 395.07474 (Kcal/Mol)<br>NImag=0<br>Htot= -1767.279437<br>Gtot= -1767.383899	HF= -1766.2766448<br>ZPE= 397.24288 (Kcal/Mol)<br>NImag=0<br>Htot= -1765.605673<br>Gtot= -1765.709098
CPU Time	0 d 22 h 38 m 49.7 s	1 d 19 h 13 m 19.8 s	2 d 1 h 8 m 45.8 s
Method	BP86	B3LYP	PBE1PBE
Basis set	aug-cc-pvdz	aug-cc-pvdz	aug-cc-pvdz
Complex **2**	No convergence criteria met during SCF Cycle even after 500 Steps	No convergence criteria met during SCF Cycle even after 500 Steps	No convergence criteria met during SCF Cycle even after 500 Steps
CPU Time	-	-	-

7.2. Thermochemistry

For basic thermochemistry, molecular structures were optimised using the pure density functional (DF) BP86 in combination with the LANL2DZ basis set and corresponding ECP at Ti and the TZVP basis set on all other atoms (notation BP86/LANL2DZ/TZVP). All optimised structures were confirmed as minima by frequency analyses.

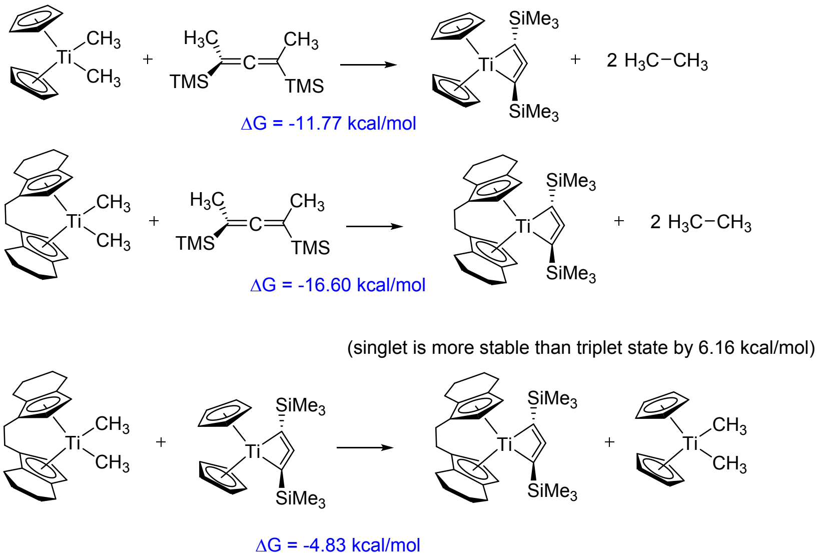

Figure S47: Calculated Gibbs free energies of isodesmic titanocene reactions**.**

Table S10: Summary of older Thermochemistry investigations data taken from reference 21.

Calculated Gibbs Free Energies (kcal·mol^{-1}) for the syntheses of 1-metallacyclobuta-2,3-dienes and metallacyclopenta-2,3,4-trienes.		
precursor	Cp_2TiMe_2	$Cp^*_2TiMe_2$
$Me(Me_3Si)C_3(SiMe_3)Me$	-11.77	-1.75[t]

[t] Energy given for the more stable triplet state.

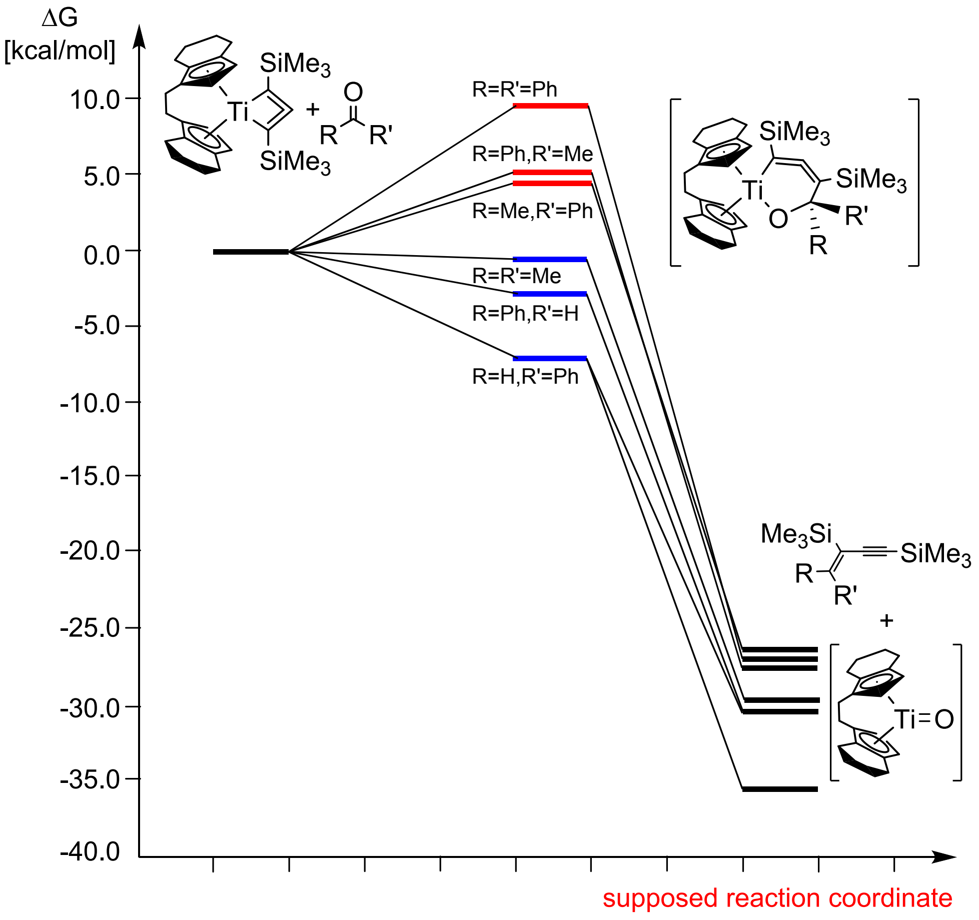

Figure S48: Representation of the thermodynamic Gibbs free energy alongside the supposed reaction pathway from complex **2** to enynes.

Table S11: Summary of thermodynamic parameters.

C2H6 Ethane	HF=-79,8519427<br>ZPE=45,52819<br>NImag=0	Htot=-79,774926<br>Gtot=-79,800838
Acetone	HF= -193.2237985<br>ZPE= 50.81801 (Kcal/Mol)<br>NImag=0	Htot= -193.136440<br>Gtot= -193.170881
Acethophenone	HF= -385.0174121<br>ZPE= 83.61406 (Kcal/Mol)<br>NImag=0	Htot= -384.875097<br>Gtot= -384.917243
Benzophenone	HF= -576.8079739<br>ZPE= 116.08201 (Kcal/Mol)<br>NImag=0	Htot= -576.610865<br>Gtot= -576.661001
Benzaldehyde	HF= -345.6852963<br>ZPE= 66.55884 (Kcal/Mol)<br>NImag=0	Htot= -345.571757<br>Gtot= -345.609941
Me(TMS)C=C=C(TMS)(Me)	HF=-1012,8327909<br>ZPE=192,25751<br>NImag=0	Htot=-1012,502676<br>Gtot=-1012,577370

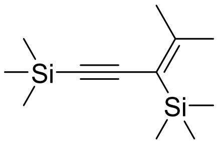	HF= -1050.9348361<br>ZPE= 195.68328 (Kcal/Mol)<br>NImag=0	Htot= -1050.598446<br>Gtot= -1050.676060
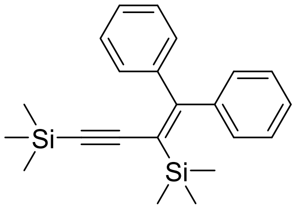	HF= -1434.5150429<br>ZPE= 260.27013 (Kcal/Mol)<br>NImag=0	Htot= -1434.069446<br>Gtot= -1434.162765
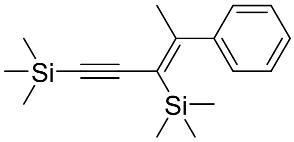	HF= -1242.7244853<br>ZPE= 228.02910 (Kcal/Mol)<br>NImag=0	Htot= -1242.333461<br>Gtot= -1242.419220
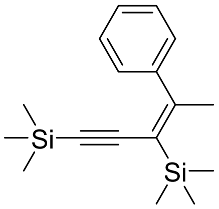	HF= -1242.723095<br>ZPE= 227.90959 (Kcal/Mol)<br>NImag=0	Htot= -1242.332141<br>Gtot= -1242.418703
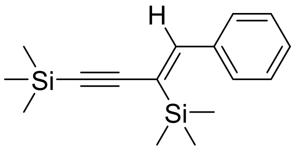	HF= -1203.3978355<br>ZPE= 211.10773 (Kcal/Mol)<br>NImag=0	Htot= -1203.035395<br>Gtot= -1203.117620
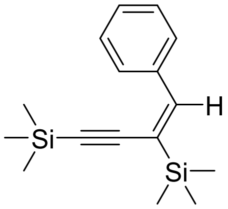	HF= -1203.4063125<br>ZPE= 211.26345 (Kcal/Mol)<br>NImag=0	Htot= -1203.043646<br>Gtot= -1203.126143
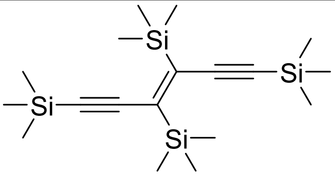	HF= -1865.944713<br>ZPE= 211.26345 (Kcal/Mol)<br>NImag=0	Htot= -1865.440132<br>Gtot= -1865.554355
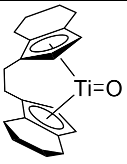	HF= -910.3218793<br>ZPE= 239.12409 (Kcal/Mol)<br>NImag=0	Htot= -909.919892<br>Gtot= -909.988009
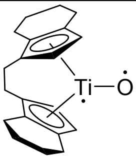	HF= -910.2686683<br>ZPE= 238.20775 (Kcal/Mol)<br>NImag=0	Htot= -909.867827<br>Gtot= -909.938284

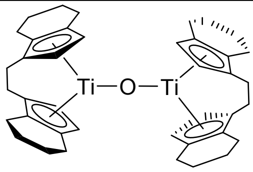<br>singlet	HF= -1745.3725286<br>ZPE= 476.95459 (Kcal/Mol)<br>NImag=0	Htot= -1744.571429<br>Gtot= -1744.681366
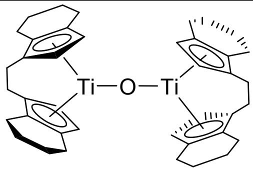<br>triplet	HF= -1745.3961018<br>ZPE= 477.36710 (Kcal/Mol)<br>NImag=0	Htot= -1744.594394<br>Gtot= -1744.704846
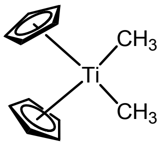	HF=-525,2039434<br>ZPE=145,00863<br>NImag=0	Htot=-524,957105<br>Gtot= -525,011891
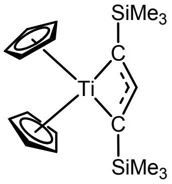	HF=-1378,3420582<br>ZPE=247,43795<br>NImag=0	Htot=-1377,917117<br>Gtot=-1378,006339
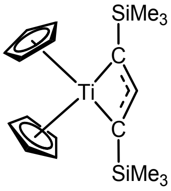	HF=-1378,3382555<br>ZPE= 246,58001<br>NImag=0	Htot= -1377,914017<br>Gtot= -1378,007893
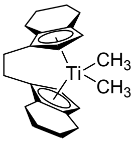	HF=-914.8469143<br>ZPE=281.21685<br>NImag=0	Htot=-914.375636<br>Gtot= -914,444743
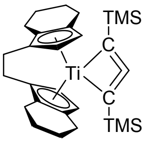	HF=-1767.9906562<br>ZPE=383.54489<br>NImag=0	Htot=-1767,340175<br>Gtot= -1767,446884
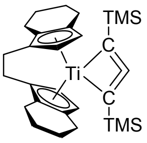<br>Singlet geometry, single point as triplet	HF= -1767.9679173<br>ZPE=383.34087 (Kcal/Mol)<br>NImag=0	Htot= -1767.317685<br>Gtot= -1767.426070
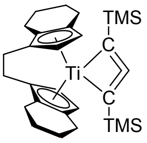	HF=-1767.9782131<br>ZPE=382.82091<br>NImag=0	Htot= -1767.328571<br>Gtot= -1767.437064

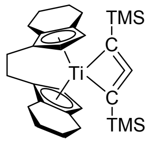<br>Triplet geometry, single point as singlet	HF=-1767.9805115<br>ZPE=382.58993 (Kcal/Mol)<br>NImag=0	Htot= -1767.330883<br>Gtot= -1767.439852
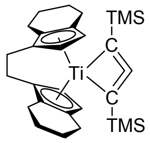<br>Singlet start geometry, BP86 opt freq<br>with guess=mix input	HF= -1767.9908489<br>ZPE= 383.91670 (Kcal/Mol)<br>NImag=0	Htot= -1767.339949<br>Gtot= -1767.445652
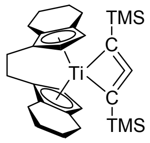<br>Singlet start geometry, UBP86 opt freq<br>with guess=mix input	HF= -1767.9908489<br>ZPE= 383.91669 (Kcal/Mol)<br>NImag=0	Htot= -1767.339949<br>Gtot= -1767.445652
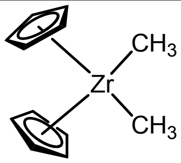	HF=-513,7210902<br>ZPE= 144,24004<br>NImag=0	Htot= -513,474949<br>Gtot= -513,532270
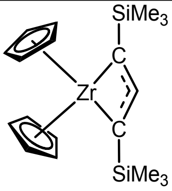	HF=-1366,8563606<br>ZPE=247,15003<br>NImag=0	Htot=-1366,431492<br>Gtot=-1366,522257
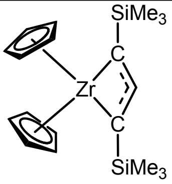	HF=-1366,8414822<br>ZPE= 246,51335<br>NImag=0	Htot= -1366,417185<br>Gtot= -1366,512687
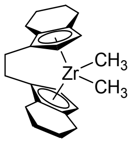	HF=-903.3606657<br>ZPE=279.98144<br>NImag=0	Htot=-902.889729<br>Gtot= -902,963903
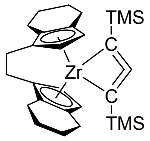	HF=-1756.5003188<br>ZPE=382.83983<br>NImag=0	Htot=-1755,850499<br>Gtot= -1755,958966
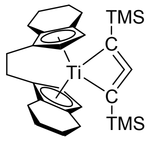	HF=-1756.4780548<br>ZPE=381.82002<br>NImag=0	Htot= -1755,829347<br>Gtot= -1755,941757

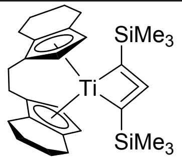 **2**	HF= -1767.9906562<br>ZPE= 383.54489 (Kcal/Mol)<br>NImag=0<br><br>C2 Symmetry	Htot= -1767.340175<br>Gtot= -1767.446884
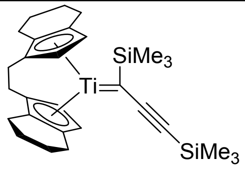<br>HF=-1767.95<br>Start geometry optimised as<br>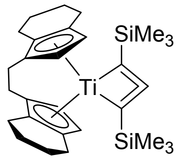 **2**	HF= -1767.9907818<br>ZPE= 383.93788 (Kcal/Mol)<br>NImag=0<br><br>C1 Symmetry	Htot= -1767.339917<br>Gtot= -1767.446318
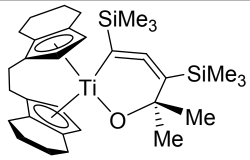	HF= -1961.2436841<br>ZPE= 437.24401 (Kcal/Mol)<br>NImag=0	Htot= -1960.502681<br>Gtot= -1960.618499
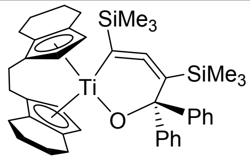	HF= -2344.8111197<br>ZPE= 500.91349 (Kcal/Mol)<br>NImag=0	Htot= -2343.961642<br>Gtot= -2344.094280
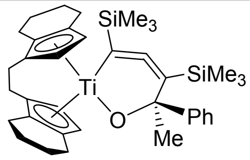	HF= -2153.0280928<br>ZPE= 469.23912 (Kcal/Mol)<br>NImag=0	Htot= -2152.232710<br>Gtot= -2152.356475
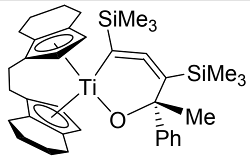	HF= -2153.02614<br>ZPE= 469.06816 (Kcal/Mol)<br>NImag=0	Htot= -2152.230925<br>Gtot= -2152.356133
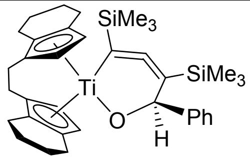 a	HF= -2113.7122202<br>ZPE= 452.18786 (Kcal/Mol)<br>NImag=0	Htot= -2112.945347<br>Gtot= -2113.068197
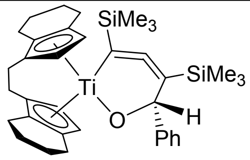 e	HF= -2113.7050992<br>ZPE= 452.38696 (Kcal/Mol)<br>NImag=0	Htot= -2112.938017<br>Gtot= -2113.059902

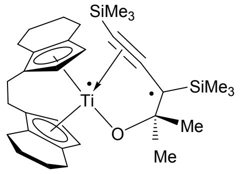	HF= -1961.2050267<br>ZPE= 436.26944 (Kcal/Mol)<br>NImag=0	Htot= -1960.465217<br>Gtot= -1960.582759
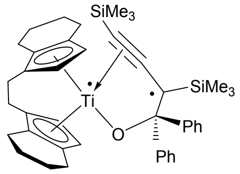	HF= -2344.7728195<br>ZPE= 500.41193 (Kcal/Mol)<br>NImag=0	Htot= -2343.924130<br>Gtot= -2344.056686
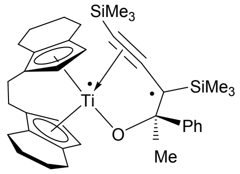	HF= -2152.9894179<br>ZPE= 467.91843 (Kcal/Mol)<br>NImag=0	Htot= -2152.195462<br>Gtot= -2152.322691
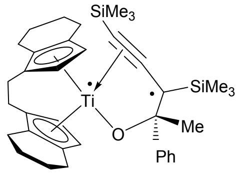	HF= -2152.993494<br>ZPE= 468.02333 (Kcal/Mol)<br>NImag=0	Htot= -2152.199549<br>Gtot= -2152.326093
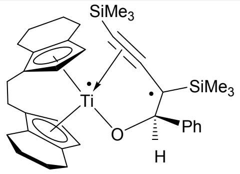	HF= -2113.6769363<br>ZPE= 450.78626 (Kcal/Mol)<br>NImag=0	Htot= -2112.911644<br>Gtot= -2113.037418
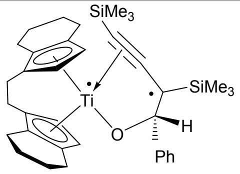	HF= -2113.6710247<br>ZPE= 451.15989 (Kcal/Mol)<br>NImag=0	Htot= -2112.905523<br>Gtot= -2113.029318

7.3. MO and DFT studies of *rac*-(ebthi)$TiC_3(SiMe_3)_2$ (2)

To obtain a better understanding of the bonding situation in titana-cyclobutadiene **2**, several single-point calculations were performed: firstly, the Kohn-Sham (KS) wave function was recalculated using the pure DF BP86 in conjunction with the def2-TZVP basis on all atoms; secondly, a hybrid DF was employed (B3LYP[12a,13]/def2-TZVP); and lastly, the canonical MOs were calculated at the HF/def2-TZVP level of theory. All (KS) wave functions were tested with respect to RHF/UHF or RKS/UKS instabilities, in order to analyse the biradical character of Ti complex **2**. While the KS wave function based on the pure DF (BP86) showed no instabilities, the hybrid DF (B3LYP) and HF solution exhibited a low-lying, “broken-symmetry” open-shell singlet state. This kind of behaviour is often observed if the biradical character is not too large,[22] since part of the non-dynamic correlation is treated by the exchange-correlation functional of the (pure) density functional. Mixing in exact exchange reduces the amount of non-dynamic correlation treated by the DF and thus the “broken-symmetry” solution becomes more stable.

In consequence, structures that were optimised using the BP86 functional are expected to show good agreement with experimental structures (as verified by comparison with structural data from single-crystal X-ray diffraction, *cf.* Table S12). The electronic energy, however, should be considered as a rough approximation due to incorrect treatment of the non-dynamic correlation.

Table S12: Comparison of experimental and calculated structural data of **2**.

	SC-XRD	BP86/LANL2DZ/TZVP		BS-UB3LYP/def2-TZVP	
Ti1–C1	2.2287(14)	2.250	+0.021	2.332	+0.103
Ti1–C3	2.2349(15)	2.250	+0.015	2.332	+0.097
C1–C2	1.303(2)	1.316	+0.013	1.298	−0.005
C2–C3	1.308(2)	1.316	+0.008	1.298	−0.010
C1–Si1	1.8370(15)	1.866	+0.029	1.846	+0.009
C3–Si2	1.8326(16)	1.866	+0.033	1.846	+0.013
Ti1–C1–C2	74.94(9)	70.39	−4.55	69.53	−5.41
Ti1–C3–C2	75.15(9)	70.39	−4.76	69.53	−5.62
Si1–C1–C2	136.71(12)	133.83	−2.88	138.04	+1.33
Si2–C3–C2	134.80(13)	133.83	−0.97	138.04	+3.24
Σ(∡C1)	352.7(3)	353.19	+0.49	357.32	+4.62
Σ(∡C3)	353.7(3)	353.19	−0.51	357.32	+3.62
C1–C2–C3	150.08(15)	150.37	+0.29	155.09	+5.01
Si1–C1–C3–Si2	68.0(2)	63.04	−4.96	41.55	−26.45

7.4. Biradical character

The "broken-symmetry" solution is not a true eigenfunction of the S^2 operator. In fact, it may be considered as a 50:50 mixture of the singlet and triplet state, if the overlap between the singly occupied orbitals and spin polarisation are small.[23,24,25] The actual singlet wave function can then be expressed in terms of a linear combination of *two* "broken-symmetry" wave functions

$$^1\Psi = \frac{1}{\sqrt{2}}\left(|\cdots\chi_+\bar{\chi}_-\rangle - |\cdots\bar{\chi}_+\chi_-\rangle\right)$$

where χ_+, χ_- are the singly occupied orbitals and the overline indicates *β* spin. Therefore, the open-shell singlet must be described by a multi-reference wave function.

In the "broken-symmetry" picture, the singly occupied orbitals χ_+ and χ_- are, in principle, localised orbitals formed by linear combinations of the (delocalised) canonical HOMO ϕ_H and LUMO ϕ_L:

$$\chi_\pm = \frac{1}{\sqrt{2}}(\phi_H \pm \phi_L)$$

Hence, the multi-reference wave function expressed in terms of the canonical MOs is given by

$$^1\Psi = c_1|\cdots\phi_H^2\rangle + c_2|\cdots\phi_L^2\rangle$$

where the expansion coefficients c_i are the square roots of the relative weight of each determinant. This type of multi-determinant open-shell singlet wave function can be obtained by the Complete Active Space (CAS) SCF method[21–29] and gives a qualitatively correct description of the electronic structure of a biradical. The biradical character can be evaluated as

$$\beta = \frac{2c_2^2}{c_1^2 + c_2^2}$$

where a value of $\beta = 1$ indicates a "perfect" biradical with two electrons in two degenerate orbitals.[24,26] Smaller values indicate an increasing energy gap between HOMO and LUMO, and $\beta \rightarrow 0$ indicates a closed-shell species.

Consequently, the smallest active space to properly describe a biradical is a CAS(2,2) calculation (*i.e.* two electrons in two orbitals). In case of compound **2**, we chose to include eight electrons in nine orbitals in the active space (comprising the formal π orbitals at the ligand and d-orbitals at Ti, *vide infra*), as these orbitals are energetically relatively closely spaced. The calculations show that the largest contributions to the multi-determinant wave function are the two determinants placing two electrons either in the formal HOMO (ϕ_4) or LUMO (ϕ_5, Figure S49; β = 28 %).

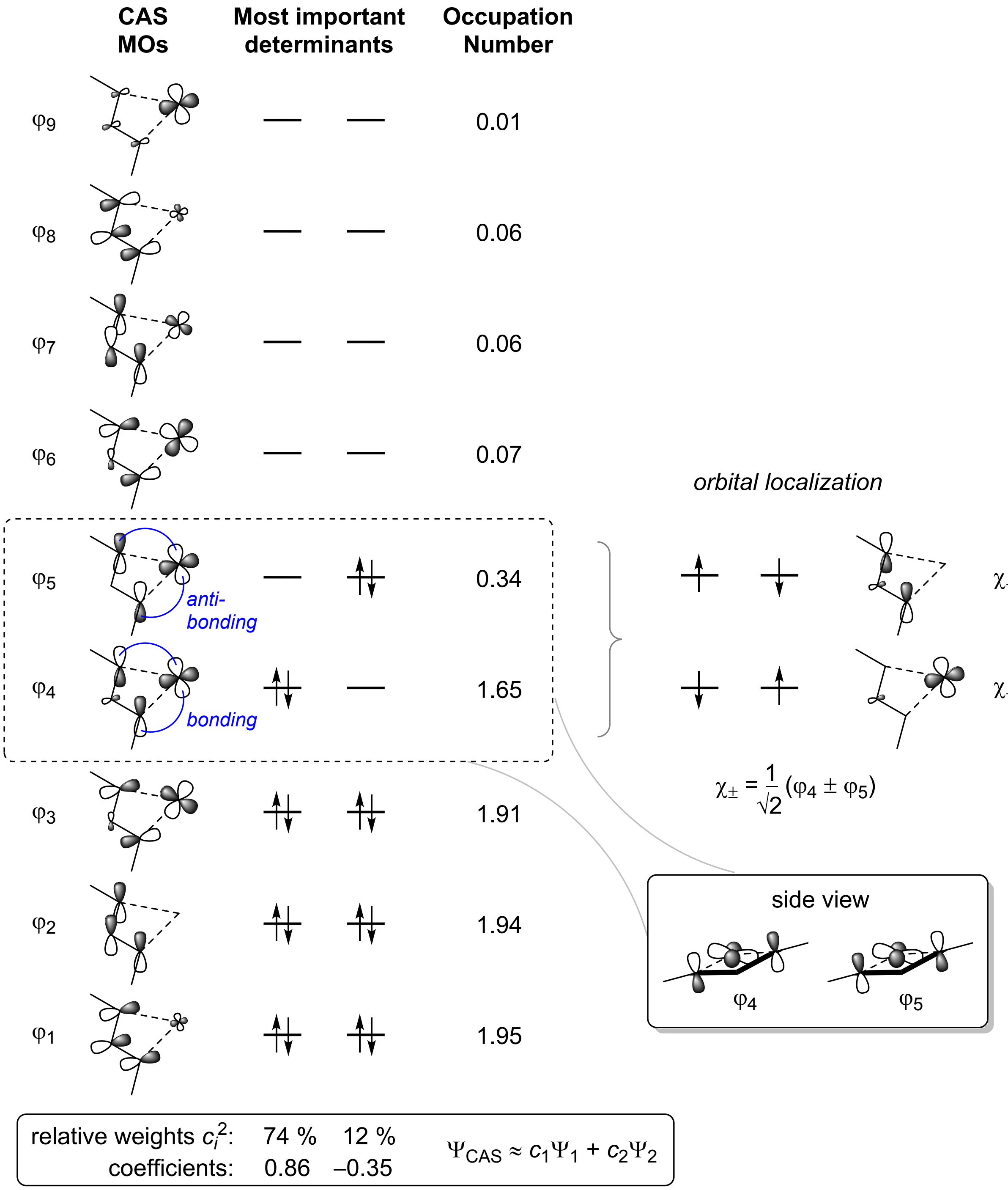

Figure S49: Schematic depiction of the active orbitals of a CAS(8,9) calculation. Only contributions to the wave function with relative weights > 1 % are shown. The orbital localisation scheme indicates that one of the radical centres is localised at Ti, while the other is delocalised across the C_3 backbone.

Hence, compound **2** can be regarded as a biradical. The singlet state is calculated to be the ground state ($\Delta E_{S\text{-}T}$ = −39.0 kJ/mol); i.e. the radical centres are antiferromagnetically coupled. The calculated exchange coupling constant[27] is

$$2J = E_S - E_T = -3260.1\ cm^{-1}$$

The radical centres are localised at Ti and on the C_3 backbone of the ligand (Figure S49, right). Therefore, the electronic structure can be understood as a complex between a formal Ti(III) fragment and an organic radical, whose “free” electrons are antiferromagnetically coupled. (This, by the way, is also indicated by the BS-B3LYP calculations; however, these results will not be discussed further as BS

calculations predict unphysical spin polarisation.) Therefore, complex **2** should be EPR silent in its ground state.

7.5. Lewis resonance scheme

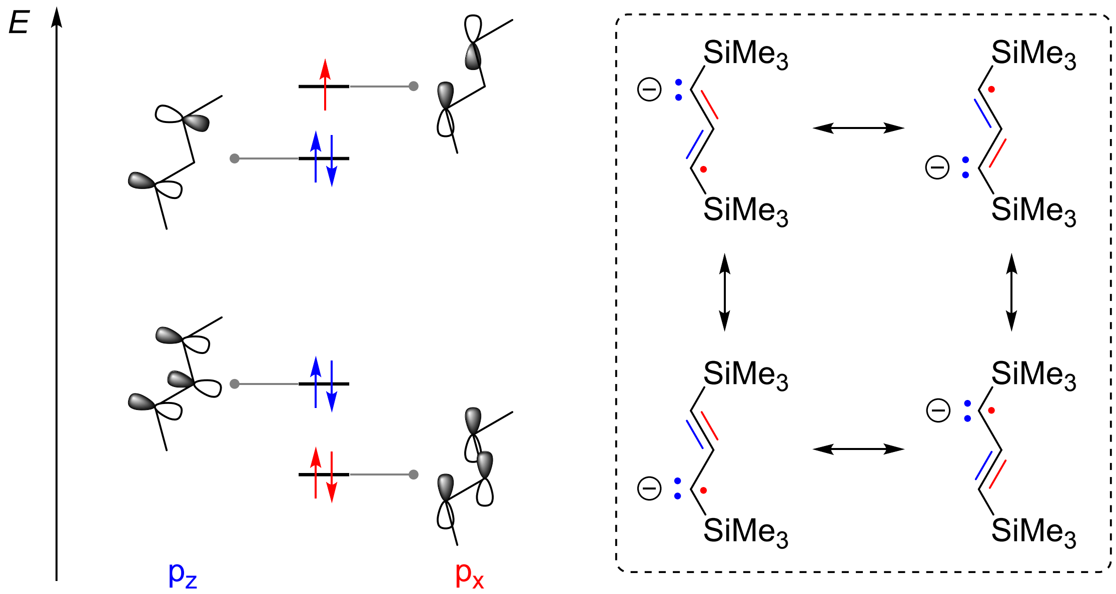

Figure S50: Left: Schematic MO diagram of the formal π-type orbitals of the ligand system. There is a 4e3c bond in the z plane (blue) and a 3e3c bond in the x plane (red). Right: Lewis resonance scheme. The electrons in p_z (p_x) orbitals are indicated in blue (red). Each π-bonding system is independently delocalized across the C_3 unit.

Analysis of the ligand-centred orbitals shows that there are two formal π bonding systems. One of them is *in-plane* with the TiC_3 ring system and acts as σ donor (ϕ_1, ϕ_3, ϕ_6, ϕ_8); the other is perpendicular to the ring and contains the delocalised radical centre (ϕ_2, ϕ_4, ϕ_5, ϕ_7). The ligand could be considered as a propadienylide anion, i.e. the one-electron reduced congener of propynylidene,[28] which is corroborated by the fact that the ligand-centred orbitals in the complex nicely correspond to the MOs of the isolated ligand system (Figure S50). Note that the electrons in both the formal π_x and π_z bonding systems are delocalized across the C_3 unit and that each of these π-bonding systems can be interpreted independently of the other, resulting in a variety of different Lewis resonance structures.

Therefore, the leading resonance structures of complex **2** are proposed as depicted in Scheme S2.

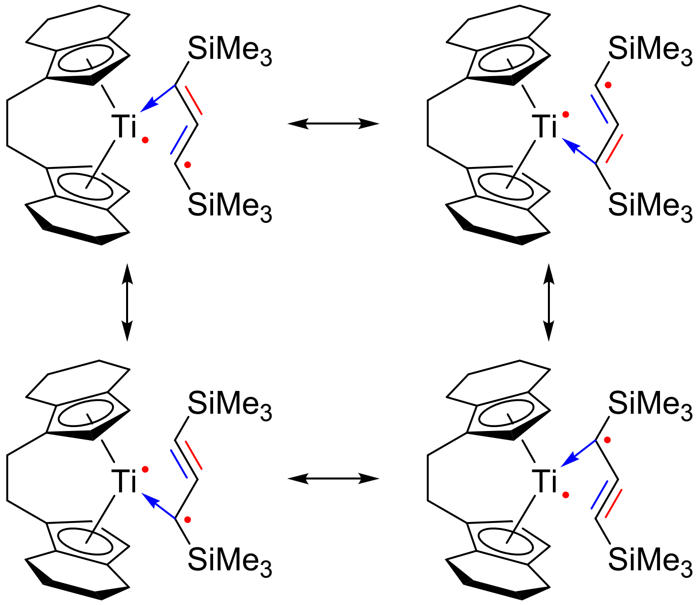

Scheme S2: Leading Lewis resonance structures of complex **2**. The electrons associated with the p_z and p_x orbitals are indicated in blue and red, respectively. Formal charges omitted for clarity.

7.6. NBO analysis

NBO analyses[19] of the BP86/def2-TZVP and CAS(8,9)/def2-TZVP densities led to similar results. The NBO routine found a double bond between both C1 and C2 as well as C2 and C3, in agreement with the Lewis structures in Scheme S2. It is worthy to note that both π-type NBOs are only occupied by approx. 1.6 electrons, indicating that the double bonds are delocalised. Furthermore, there are formally two Ti–C σ-bonds (Ti1–C1 and Ti1–C3) which are occupied by 1.5 electrons each. This can be attributed to both the delocalisation of the Ti–C bond (*vide supra*) as well as the biradical character, which is not well represented in the NBO picture.

The calculated natural charge of the $C_3(SiMe_3)_2$ ligand amounts to −0.39 *e* (CAS) or −0.64 *e* (BP86), which is in the expected range of a formally anionic ligand.

7.7. QT-AIM analysis

QT-AIM analysis[29] revealed two Ti–C “bond” paths (Ti1–C1 and Ti1–C3), in agreement with the Lewis resonance scheme (Scheme S2). Despite the short interatomic distance between Ti1 and C2, there is no strong bonding interaction between those atoms; on the contrary, a ring critical point is found near the centre of the TiC_3 ring system (i.e. there is a minimum in electron density within the ring plane). Moreover, the Laplacian of the electron density $\nabla^2 r$ indicates that the Ti–C bonds are strongly polarised towards the C atoms, in agreement with their description as dative bonds. (Figure S51).

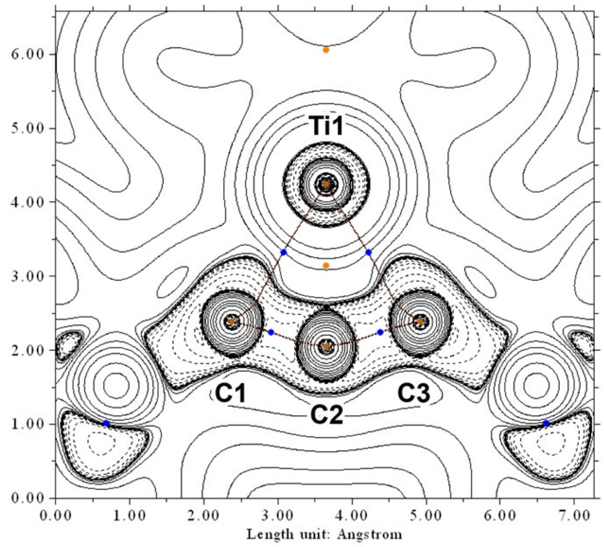

Figure S51: Contour plot of the Laplacian of the electron density $\nabla^2 r$ of Ti complex **1** in the TiC_3 ring plane. Dashed lines indicate negative (local charge concentration), solid lines indicate positive values (local charge depletion). The Laplacian plot is overlaid with the molecular graph from QT-AIM analysis. Brown lines indicate bonding paths, blue dots correspond to bond critical points, orange points indicate ring critical points. Density from CAS(8,9)/def2-TZVP calculation.

The densities obtained from CAS(8,9) and BP86 calculations are quite similar, indicating that the pure DFT method is suitable to approximately describe the electron density despite its single-determinant character (Figure S52)

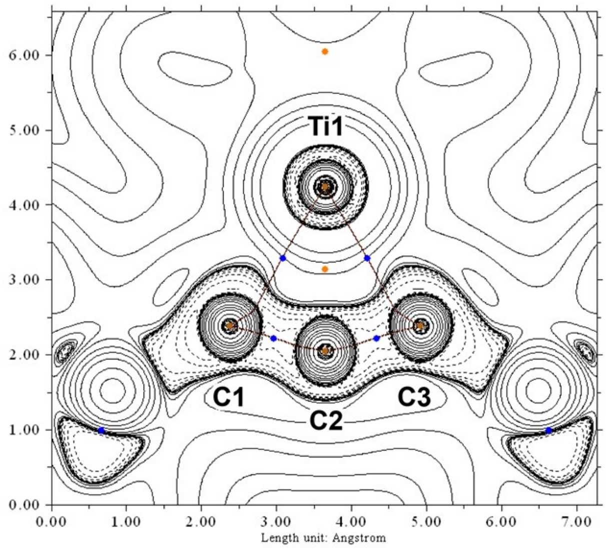

Figure S52: Same as Figure S51, but density taken from BP86/def2-TZVP calculation.

7.8. Electron Localisation Function

The results from QT-AIM analysis are corroborated by ELF analysis (Figure S53). There is no localised electron density in the valence region of C2 directed towards Ti1, whereas the bonding electrons between C1/C3 and Ti are localised in approx. the same region of space as indicated by the Laplacian of the electron density. It is worthy to note that there is no localised electron density around C2

pointing *away* from Ti1 either, i.e. there is no lone pair of electrons at the central carbon atom. Consequently, the electronic structure of the C_3 scaffold is different from that of structurally related bent allenes, such as so-called "carbodicarbenes" (Figure S54).[30]

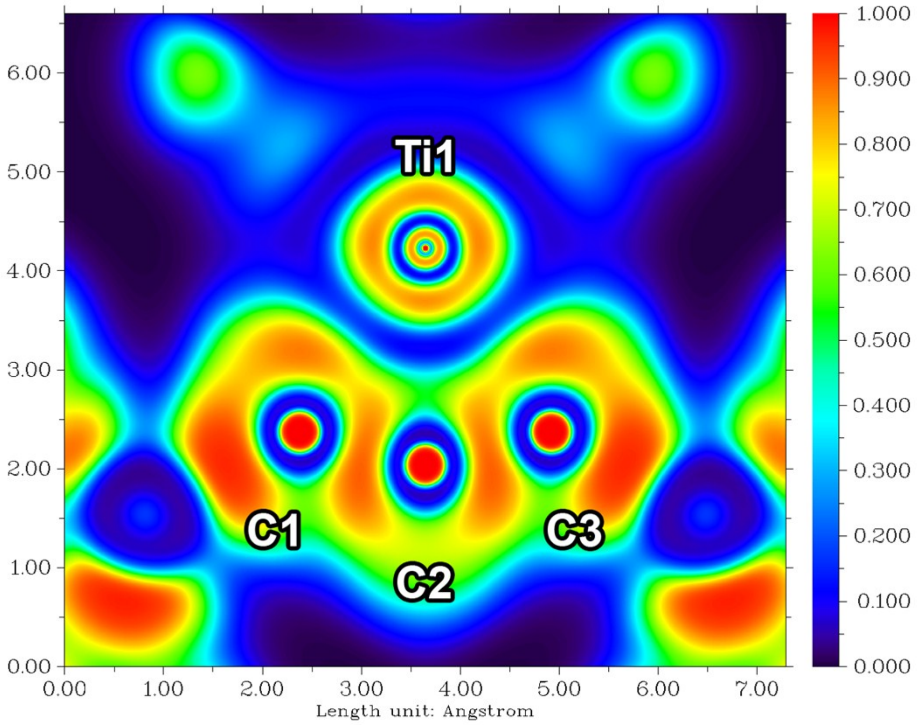

Figure S53: ELF plot of Ti complex **2** in the TiC_3 ring plane.

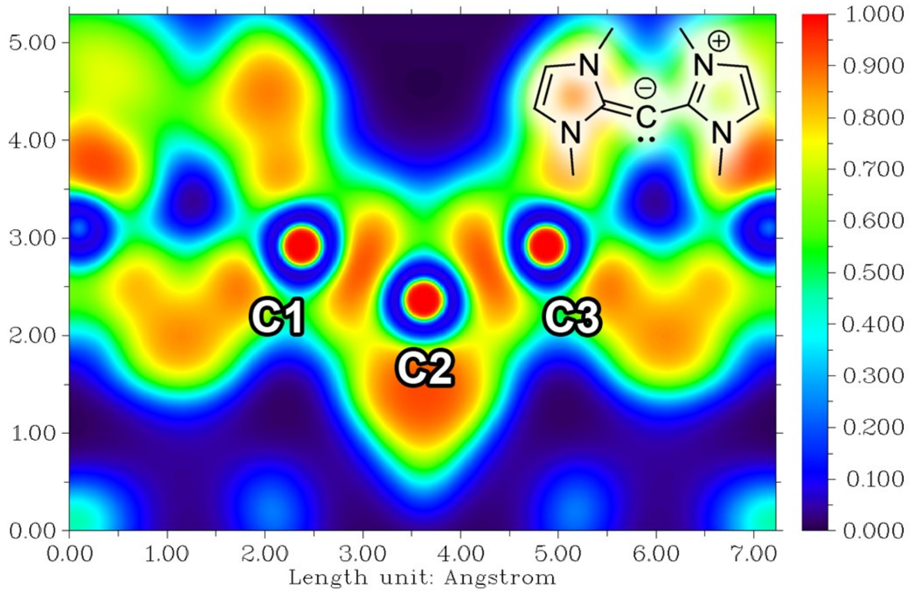

Figure S54: ELF plot of a "carbodicarbene" in the C_3 plane. The lone pair of electrons is clearly visible at C2.

7.9. CAS computations of $Cp_2TiC_3(SiMe_3)_2$ (2Cp) and $Cp^*_2TiC_3(SiMe_3)$ (2Cp*)

CAS(8,9)/def2-TZVP computations were carried out in an analogous manner for the closely related Ti complexes $Cp_2TiC_3(SiMe_3)_2$ (**2Cp**) and $Cp^*_2TiC_3(SiMe_3)$ (**2Cp***). A summary of the results is shown in Table S13.

Table S13: Results of CAS(8,9)/def2-TZVP single point calculations for **2**, **2Cp**, and **2Cp***.

Compound	β [%]	$\Delta E_{S\text{-}T}$ [kJ/mol]	$2J$ [cm^{-1}]	∑∡(C1/C3) [°]
**2** (EBTHI)	28	−39.0	−3260	353.2
**2Cp**	30	−36.2	−3025	357.1
**2Cp***	74	−7.4	−616	359.4

It should be pointed out that the singlet-triplet gap and therefore the biradical character greatly depend on the pyramidalisation of the carbon atoms C1 and C3 of the TiC_3 ring system. Since the coordination environment around C1/C3 is nearly planar in compound **2Cp*** (most likely due to steric reasons), it displays the highest biradical character. This trend is agreement with previous computations.[31]

8. Literature

[1] Reiß, F.; Reiß, M.; Spannenberg, A.; Jiao, H.; Baumann, W.; Arndt, P.; Rosenthal, U.; Beweries, T. *Chem. Eur. J.* **2018**, *24*, 5667-5674.

[2] Gottlieb, H. E.; Kotlyar, V.; Nudelman, A. *J. Org. Chem*. **1997**, *62*, 7512.

[3] Sheldrick, G. M. *Acta Cryst*. **2008**, *A64*, 112-122.

[4] Sheldrick, G. M. *Acta Cryst.* **2015**, *C71*, 3-8

[5] Diamond - Crystal and Molecular Structure Visualization, Crystal Impact - Dr. H. Putz & Dr. K. Brandenburg GbR, Kreuzherrenstr. 102, 53227 Bonn, Germany, http://www.crystalimpact.com/diamond.

[6] Gaussian 09, Revision E.01, M. J. Frisch, G. W. Trucks, H. B. Schlegel, G. E. Scuseria, M. A. Robb, J. R. Cheeseman, G. Scalmani, V. Barone, B. Mennucci, G. A. Petersson, H. Nakatsuji, M. Caricato, X. Li, H. P. Hratchian, A. F. Izmaylov, J. Bloino, G. Zheng, J. L. Sonnenberg, M. Hada, M. Ehara, K. Toyota, R. Fukuda, J. Hasegawa, M. Ishida, T. Nakajima, Y. Honda, O. Kitao, H. Nakai, T. Vreven, J. A. Montgomery, Jr., J. E. Peralta, F. Ogliaro, M. Bearpark, J. J. Heyd, E. Brothers, K. N. Kudin, V. N. Staroverov, T. Keith, R. Kobayashi, J. Normand, K. Raghavachari, A. Rendell, J. C. Burant, S. S. Iyengar, J. Tomasi, M. Cossi, N. Rega, J. M. Millam, M. Klene, J. E. Knox, J. B. Cross, V. Bakken, C. Adamo, J. Jaramillo, R. Gomperts, R. E. Stratmann, O. Yazyev, A. J. Austin, R. Cammi, C. Pomelli, J. W. Ochterski, R. L. Martin, K. Morokuma, V. G. Zakrzewski, G. A. Voth, P. Salvador, J. J. Dannenberg, S. Dapprich, A. D. Daniels, O. Farkas, J. B. Foresman, J. V. Ortiz, J. Cioslowski, and D. J. Fox, Gaussian, Inc., Wallingford CT, **2013**.

[7] Mccullough, L. G.; Listemann, M. L.; Schrock, R. R.; Churchill, M. R.; Ziller, J. W. *J. Am. Chem. Soc.* **1983**, *105*, 6729-6730.

[8] McCullough, L. G.; Schrock, R. R.; Dewan, J. C.; Murdzek, J. C. *J. Am. Chem. Soc.* **1985**, *107*, 5987-5998.

[9] Heppekausen, J.; Stade, R.; Kondoh, A.; Seidel, G.; Goddard, R.; Fürstner, A. *Chem. Eur. J.* **2012**, *18*, 10281-10299.

[10] March, J. *Advanced Organic Chemistry*, *2. Edition*, McGraw-Hill, Tokyo, **1977**.

[11] Pyykkö, P.; Atsumi, M. *Chem. Eur. J.* **2009**, *15*, 12770-12779

[12] a) Becke, A. D. *Phys. Rev. A* **1988**, *38*, 3098–3100; b) Perdew, J. P. *Phys. Rev. B* **1986**, *33*, 8822–8824.

[13] a) Vosko, S. H.; Wilk, L.; Nusair, M. *Can. J. Phys.* **1980**, *58*, 1200–1211; b) Lee, C.; Yang, W.; Parr, R. G. *Phys. Rev. B* **1988**, *37*, 785–789; c) Miehlich, B.; Savin, A.; Stoll, H.; Preuss, H. *Chem. Phys. Lett.* **1989**, *157*, 200–206; d) Becke, A. D. *J. Chem. Phys.* **1993**, *98*, 5648–5652.

[14] Adamo, C.; Barone, V. *J. Chem. Phys.* **1999,** *110*, 6158-6169.

[15] Weigend, F.; Ahlrichs, R. *Phys. Chem. Chem. Phys.* **2005**, *7*, 3297–3305.

[16] Schäfer, A.; Huber, C.; Ahlrichs, R. *J. Chem. Phys.* **1994**, *100*, 5829–5835.

[17] a) Hay, P. J.; Wadt, W. R. *J. Chem. Phys.* **1985**, *82*, 270–283; b) Hay, P. J.; Wadt, W. R. *J. Chem. Phys.* **1985**, *82*, 299–310.

[18] a) Kendall, R. A.; Dunning Jr., T. H.; Harrison, R. J. *J. Chem. Phys.* **1992**, *96*, 6796-6806; b) Woon, D. E.; Dunning Jr., T. H. *J. Chem. Phys.* **1993**, *98*, 1358-1371.

[19] a) E. D. Glendening, J. K. Badenhoop, A. E. Reed, J. E. Carpenter, J. A. Bohmann, C. M. Morales, C. R. Landis, F. Weinhold, 2013 (NBO 6.0); b) Carpenter, J. E.; Weinhold, F. *J. Mol. Struct.: THEOCHEM* **1988**, *169*, 41–62; c) Weinhold, F.; Carpenter, J. E. *The Structure of Small Molecules and Ions*, Plenum Press, **1988**; d) Weinhold, F.; Landis, C. R. *Valency and Bonding. A Natural*

Bond Orbital Donor-Acceptor Perspective, Cambridge University Press, **2005**.

[20] Lu, T.; Chen, F. *J. Comput. Chem.* **2012**, *33*, 580–592.

[21] Reiß, F.; Reiß, M.; Spannenberg, A.; Jiao, H.; Hollmann, D.; Arndt, P.; Rosenthal, U.; Beweries, T. *Chem. Eur. J.* **2017**, *23*, 14158-14162.

[22] Hegarty, D.; Robb, M. A. *Mol. Phys.* **1979**, *38*, 1795–1812.

[23] Cramer, C. J. *Essentials of Computational Chemistry: Theories and Models*, John Wiley & Sons, Ltd, Chichester, UK, **2004**.

[24] Salem, L.; Rowland, C. *Angew. Chem. Int. Ed. Engl.* **1972**, *11*, 92–111.

[25] Malrieu, J.-P.; Trinquier, G. *J. Phys. Chem. A* **2012**, *116*, 8226–8237.

[26] Miliordos, E.; Ruedenberg, K.; Xantheas, S. S. *Angew. Chem. Int. Ed.* **2013**, *52*, 5736–5739.

[27] a) Noodleman, L. *J. Chem. Phys.* **1981**, *74*, 5737–5743; b) Herebian, D.; Wieghardt, K. E.; Neese, F. *J. Am. Chem. Soc.* **2003**, *125*, 10997–11005; c) Abe, M. *Chem. Rev.* **2013**, *113*, 7011–7088.

[28] a) Seburg, R. A.; McMahon, R. J. *Angew. Chem. Int. Ed. Engl.* **1995**, *34*, 2009–2012; b) Seburg, R. A.; Patterson, E. V.; McMahon, R. J. *J. Am. Chem. Soc.* **2009**, *131*, 9442–9455.

[29] a) Bader, R. F. W. *Acc. Chem. Res.* **1985**, *18*, 9–15; b) Bader, R. F. W. *Chem. Rev.* **1991**, *91*, 893–928; c) Bader, R. F. W. *Atoms in Molecules: A Quantum Theory*, Oxford University Press, **1994**; d) Bader, R. F. W. *Monatsh. Chem.* **2005**, *136*, 819–854.

[30] a) Tonner, R.; Frenking, G. *Angew. Chem. Int. Ed.* **2007**, *46*, 8695–8698; b) Dyker, C. A.; Lavallo, V.; Donnadieu, B.; Bertrand, G. *Angew. Chem. Int. Ed.* **2008**, *47*, 3206–3209.

[31] Roy, S.; Jemmis, E. D.; Schulz, A.; Beweries, T.; Rosenthal, U. *Angew. Chem. Int. Ed.* **2012**, *51*, 5347–5350.
